# Structural Characteristics and Recent Advances in Thermoelectric Binary Indium Chalcogenides

**DOI:** 10.34133/research.0727

**Published:** 2025-06-10

**Authors:** Yasong Wu, Binjie Zhou, Lu Liu, Shengnan Dai, Lirong Song, Jiong Yang

**Affiliations:** Materials Genome Institute, Shanghai Engineering Research Center for Integrated Circuits and Advanced Display Materials, Shanghai University, Shanghai 200444, China.

## Abstract

Thermoelectric (TE) materials have garnered widespread research interest owing to their capability for direct heat-to-electricity conversion. Binary indium-based chalcogenides (In–X, X = Te, Se, S) stand out in inorganic materials by virtue of their relatively low thermal conductivity. For example, In_4_Se_2.35_ shows a low thermal conductivity of 0.74 W m^−1^ K^−1^ and an impressive *zT* value of 1.48 along the *b*–*c* plane at 705 K, as a result of structural anisotropy. Here, we review the structural features and recent research progress in the TE field for In–X materials. It begins by presenting the characteristics of crystal structure, electronic band structure, and phonon dispersion, aiming to microscopically understand the similarity/dissimilarity among these In–X compounds, notably the role of unconventional bonds (such as In–In) in modulating the band structures and lattice vibrations. Furthermore, TE optimization strategies of such materials were classified and discussed, including defect engineering, crystal orientation engineering, nanostructuring, and grain size engineering. The final section provides an overview of recent progress in optimizing TE properties of indium tellurides, indium selenides, and indium sulfides. An outlook is also presented on the major challenges and opportunities associated with these material systems for future TE applications. This Review is expected to provide critical insights into the development of new strategies to design binary indium-based chalcogenides as promising TE materials in the future.

## Introduction

Efficient and clean energy is increasingly in demand owing to the growing energy and environmental crisis [1,2]. To tackle the issue of energy supply shortage and prevent further environmental degradation, it is crucial to develop renewable energy devices. One promising solution is the utilization of thermoelectric (TE) materials, which can directly convert thermal energy into electricity [3–5]. TE energy converters have gained substantial interest due to their advantages, for example, no mechanical moving parts, reliability, quiet operation, and environmental friendliness [6,7]. As a result, TE conversion technology has promising applications in aerospace, biomedicine, integrated circuits, etc. [8–11]. However, despite the potential advantages of TE technology, the current conversion efficiency of TE devices remains low, limiting their commercial applications [12]. Therefore, researchers are continuously exploring and developing new high-performance TE materials to attain high conversion efficiency and broaden the application of TE devices.

The efficiency of TEs to convert thermal energy into electricity is crucially indicated by the dimensionless figure of merit, *zT*, which is expressed by the following formula [13,14]:

$$\mathrm{zT}=\frac{S^{2}\sigma T}{\kappa_{e}+\kappa_{L}}$$ (1)

where *S*, *σ*, *κ*_e_, *κ*_L_, and *T* are the Seebeck coefficient, electrical conductivity, electronic thermal conductivity, lattice thermal conductivity, and absolute temperature, respectively. Nevertheless, attaining a high *zT* value is intricate owing to the interplay between these TE parameters. Boosting the *zT* value necessitates the strategic decoupling of electrical and thermal transport properties [15], a substantial research hurdle in the field of TEs [16].

**Fig. 1.**
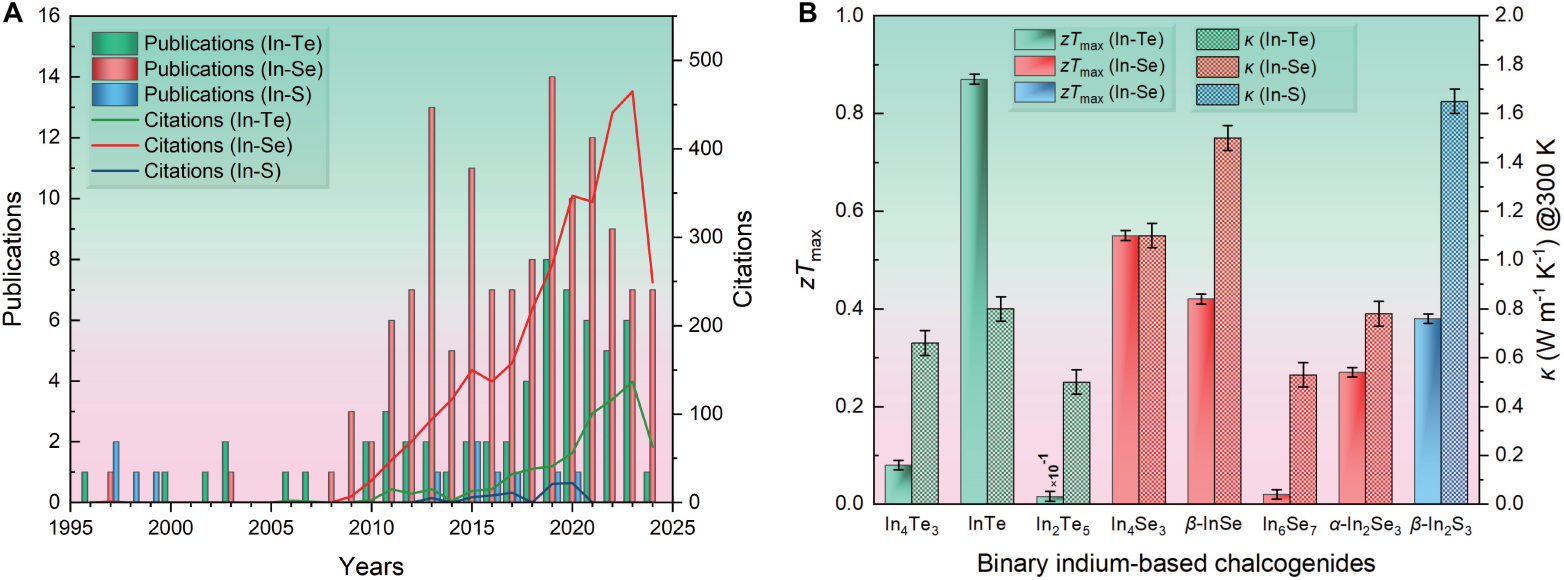
(A) Timeline of publications and citations for In–X (X = Te, Se, S) material systems in the TE field (data retrieved from Web of Science). (B) Reported total thermal conductivity at 300 K (with ±1% error bars) and *zT*_max_ values (with ±5% error bars) for pure binary indium-based chalcogenides [53,77,89,102,103,106,124,127].

Currently, inorganic semiconductor materials such as Bi_2_Te_3_ [17–19], PbTe [20–22], GeTe [23–25], and SiGe [26–28] are showing great promise in the field of TEs. Among them, binary indium-based chalcogenides (e.g., In_4_Se_3_ [29] and InTe [30]) have earned substantial attention because of their unique structure and impressive TE performance. Indium exhibits versatility and flexibility in forming binary chalcogenides, enabling the formation of various compounds with different stoichiometric ratios, resulting in a complex material system. Indium tellurides include In_4_Te_3_, InTe, In_3_Te_4_, In_2_Te_3_, In_2_Te_5_, etc. Indium selenides contain In_4_Se_3_, InSe, In_6_Se_7_, In_3_Se_4_, In_2_Se_3_, etc. Indium sulfides comprise InS, In_6_S_7_, In_3_S_4_, and In_2_S_3_. In binary chalcogenides, indium can bond to other atoms via ionic or covalent bonds, and generally tend to exhibit mixed valence states. For example, InTe, i.e., In^+^In^3+^Te_2_, contains both In^1+^ and In^3+^ cations. Owing to exceptional nonlinear effects, high damage threshold, remarkable photoresponsivity, and optimal band gap, InX compounds (X = Te, Se, S) are extensively applied in fields such as optoelectronic devices, nonlinear optics, and ultrafast lasers [31–35]. Nonetheless, binary indium-based chalcogenides, especially In–Te and In–Se systems, have attracted incremental consideration in the TE research field (Fig. 1A), owing to their intrinsically low thermal conductivity of <2 W m^−1^ K^−1^ at 300 K. Moreover, several pure In–X materials (e.g., InTe and In_4_Se_3_) exhibit the maximum *zT* (*zT*_max_) value higher than 0.5 (see Fig. 1B).

This review offers a thorough overview of recent advancements in TE research on binary indium-based chalcogenides. We first focus on presenting and comparing the crystal structures for those binary indium-based chalcogenides that have the same In-to-chalcogen ratio at ambient conditions, e.g., InTe versus InSe versus InS. Afterward, the band structures and phonon dispersions are analyzed to better understand the role of unconventional bonds present in certain In–X compounds. Moreover, this review also analyzes and summarizes the current experimental TE optimization strategies of these compounds, including defect engineering, crystal orientation engineering, nanostructuring, and grain size engineering. Additionally, the review discusses the existing challenges and future development of binary indium-based chalcogenides, with the expectation of providing some new insights on how to further enhance their TE properties and promote their large-scale application. Overall, this Review serves as a valuable reference for those working in the TE field and offers a thorough overview of the progress and potential of binary indium-based chalcogenides as TEs.

## Crystal Structure

### In_4_Te_3_ and In_4_Se_3_

**Fig. 2.**
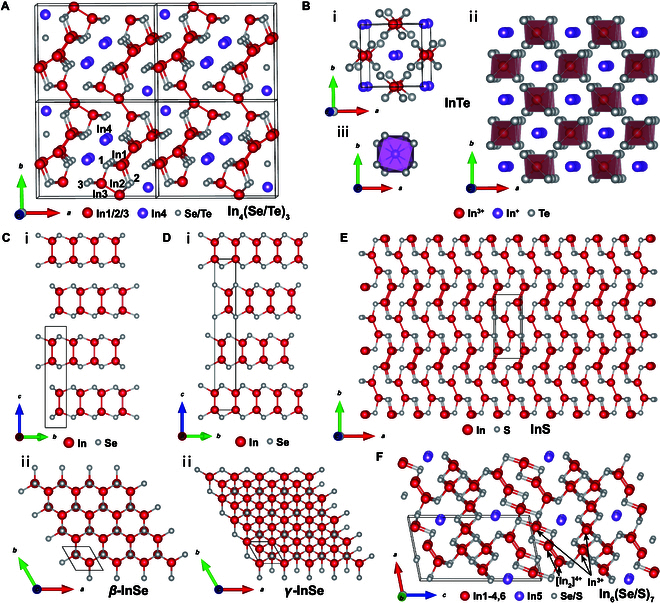
(A) Crystal structure of In_4_(Se/Te)_3_. (B) Crystal structure of InTe. (i) Single-unit cell, (ii) diagram of the InTe structure with a covalent chain of [In^3+^Te^2−^_4/2_]^−^ along the *c* axis, and (iii) the In^1+^ centered distorted square antiprismatic configuration of Te atoms. (C) (i) Side view and (ii) top view of *β*-InSe. (D) (i) Side view and (ii) top view of *γ*-InSe. (E) Crystal structure of InS. (F) Crystal structure of In_6_(Se/S)_7_.

The In_4_Se_3_ compound crystallizes in an orthorhombic system with the space group of *Pnnm* (no. 58), as determined by Hogg et al. [36]. The lattice parameters are *a* = 15.297 Å, *b* = 12.308 Å, and *c* = 4.081 Å. In_4_Se_3_ exhibits layered structure with distorted layers of nonplanar surfaces connected by weak interactions. The structure is a result of the bonding between atoms with mixed valence states, specifically In_4_Se_3_ = [In]^+^[In_3_]^5+^[Se^2−^]_3_ [37]. Referring to Fig. 2A, each quasi-2-dimensional In/Se layer consists of one-dimensional In/Se chains, and adjacent layers are stacked along the *a* axis through van der Waals interactions. The one-dimensional In/Se chain comprises trinuclear [(In_3_)^5+^(Se_3_)^6−^] clusters [38], where In1, In2, and In3 atoms form a quasi-one-dimensional chain with metallic bonding interactions. These In atoms are covalently bonded respectively to 3 differently positioned Se atoms (Se1 to Se3) in the *b*–*c* plane [38]. In1 occupies an edge position in the (In_3_)^5+^ chain, tetrahedrally surrounded by 3 selenium atoms (2 Se1 and 1 Se2) as well as 1 In2. In2 is positioned at the center of the (In_3_)^5+^ chain, encircled by 2 Se2 atoms, 1 In1, and 1 In3. Similar to In1, In3 is in the position of the chain termini and exhibits a tetrahedral coordination with 3 selenium atoms (2 Se3 and 1 Se1) and 1 In2. The In4 atom is located in the interlayer region, where there is a notable density of valence electrons [39,40]. The nearest indium counterpart, another In4, is situated at a notably substantial distance, exceeding 3 Å. Its distance to selenium atom is also relatively elongated (the shortest with Se3, approximately 3 Å). A Peierls distortion of the one-dimensional In/Se chain, which is caused by the charge density wave (CDW) along the *b*–*c* plane [40,41], can reduce the thermal conductivity in the *b*–*c* plane [42]. The relatively low band gap of In_4_Se_3_ compared to other In–Se compounds is mainly attributed to its unique crystal structure with the In–In bonding feature [41]. In_4_Te_3_ shows a similar crystal structure (orthorhombic *Pnnm* space group), with lattice parameters *a* = 15.730 Å, *b* = 12.784 Å, and *c* = 4.434 Å [43].

### InX (X = Te, Se, S)

InTe exhibits a TlSe-type structure under ambient conditions, belonging to the tetragonal system (space group *I*4/*mcm*). Its lattice parameters are *a* = *b* = 8.454(2) Å and *c* = 7.152(6) Å [44]. The mixed-valence formula of InTe, In^1+^[In^3+^Te^2−^_4/2_]^−^, suggested that In atoms occupy 2 different positions within the structure [45] (Fig. 2B), and the distances between indium atoms are exceedingly large, i.e., the nearest distances of In^+^-In^+^, In^3+^-In^3+^, and In^+^-In^3+^ are approximately 3.576, 3.576, and 4.227 Å, respectively. The In^3+^ cation is tetrahedrally coordinated to the Te^2−^ anions and forms an (InTe_2_)^−^ chain along the *c* axis [Fig. 2B(ii)]. The In^1+^ cation with 5s^2^ lone pair electrons is located in the center of a cage-like framework formed by the 8 surrounding Te atoms arranged in a square antiprismatic geometry [46], as shown in Fig. 2B(iii).

InSe, unlike InTe, can crystallize into both hexagonal and rhombohedral layered crystal structures, i.e., *β*-InSe and *γ*-InSe phases, respectively [41], as shown in Fig. 2C and D. *β*-InSe has lattice parameters of *a* = 4.005 Å and *c* = 16.640 Å, with the space group *P*6_3_/*mmc* [47,48]. *γ*-InSe crystallizes in the space group *R*3*m*, with lattice parameters *a* = 4.0046 Å and *c* = 24.960 Å [41]. As shown in Fig. 2C(i) and Fig. 2D(i), both structures are triangular biconical, which results in similar band gaps (will be further shown in the third section). The layers in *β*-InSe exhibit an ABAB stacking pattern, while in *γ*-InSe, the stacking pattern is ABCABC. Every layer of InSe is constituted by a network of covalently bonded Se–In–In–Se, with weaker van der Waals bonding between the layers [33]. Within *γ*-InSe, the In–In bonds are slightly shorter. Each indium atom has a tetrahedral configuration that is similar to the In1 and In3 atoms in In_4_Se_3_.

In 2000, Hollingsworth et al. [49] identified 2 different crystalline phases of InS compounds: a more-stable network structure and a higher-energy (metastable) layered structure. These phases consist of the S–In–In–S basic structural units [35]. Later in 2014, Kushwaha et al. [50] demonstrated that the most stable crystalline phase was recognized to show a network structure belonging to an orthorhombic crystal system (space group *Pnnm*) (Fig. 2E), by growing InS single crystals using the In flux method. The lattice parameters were *a* = 4.4506(2) Å, *b* = 10.6503(4) Å, and *c* = 3.9455(2) Å. Notably, the minimum distances between In–In and S–S atoms (interlayer) were determined to be 2.806 Å and 3.088 Å, respectively.

### In_6_Se_7_ and In_6_S_7_

In_6_Se_7_ has a monoclinic structure with a space group of *P*2_1_/*m* [51]. The unit cell parameters are *a* = 9.433 Å, *b* = 4.064 Å, *c* = 17.663 Å, and *β* = 100.92°. The compound In_6_Se_7_ consists of In ions in various valence states (+1, +2, and +3) and can be represented as In^+^[In_2_]^4+^(In^3+^)_3_(Se^2−^)_7_ [52]. It seems to contain the characteristics of the In atom found in the InSe (triangular biconical), In_4_Se_3_ (one-dimensional In^+^), and In_3_Se_4_ (octahedral In) structures. The [In_2_]^4+^ and In^3+^ ions are distributed over 2 and 3 distinct lattice sites [53], respectively, as shown in Fig. 2F. In_6_S_7_ also shows the monoclinic structure (space group *P*2_1_/*m*). Two formula units are contained within the unit cell, and all In and S atoms are located at the special position 2e [54]. The cell parameters are *a* = 9.088 Å, *b* = 3.887 Å, *c* = 17.166 Å, and *β* =101.92° [55].

### In_3_X_4_ (X = Te, Se, S)

**Fig. 3.**
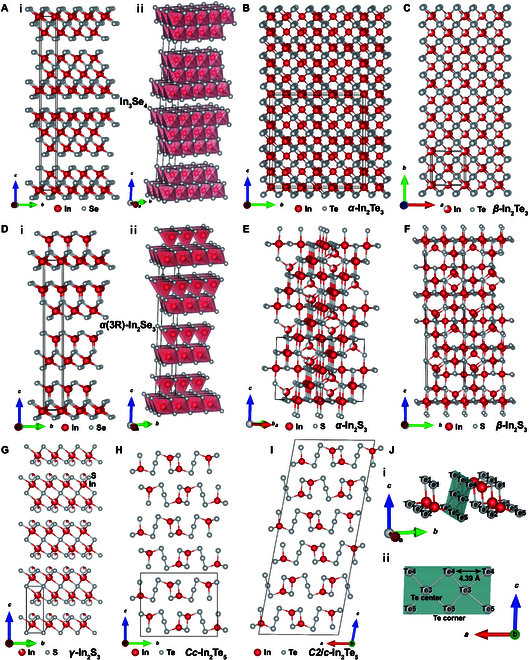
(A) (i) Crystal structure of In_3_Se_4_ and (ii) polyhedral view of the octahedral arrangement of In atoms with their surrounding Se atoms. Crystal structures of (B) *α*-In_2_Te_3_ and (C) *β*-In_2_Te_3_. (D) (i) Crystal structure of *α*(3R)-In_2_Se_3_ and (ii) polyhedral view of the tetrahedral/octahedral arrangement of In atoms with their surrounding Se atoms. Crystal structures of (E) *α*-In_2_S_3_, (F) *β*-In_2_S_3_, and (G) *γ*-In_2_S_3_. Crystal structures of (H) *Cc* and (I) *C*2/*c* phases of In_2_Te_5_, and (J) (i) a close-up view of the layered structure, with the planar-coordinated Te structural unit emphasized, and (ii) view with only Te atom rows. Reproduced with permission [76]. Copyright 2020 American Chemical Society.

In_3_Se_4_ has a layered rhombohedral crystal structure with a space group of *R*$\bar{3}$*m*. Its lattice parameters are *a* = 3.964 ± 0.002 Å and *c* = 39.59 ± 0.02 Å [56]. The crystal structure of In_3_Se_4_ is characterized by the stacking of Se–In–Se–In–Se–In–Se layers along the *c* axis [Fig. 3A(i)]. The In atoms are coordinated in an octahedral geometry, surrounded by 6 neighboring Se atoms that form the vertices of the octahedron [57] [Fig. 3A(ii)]. This structure of In_3_Se_4_ resembles the one described for In_3_Te_4_ by Geller et al. [58] [space group *R*$\bar{3}$*m*, lattice parameters *a* = *b* = 4.27(1) Å and *c* = 40.90(10) Å]. But according to Karakostas et al. [59], In_3_Te_4_ crystallizes in a tetragonal system with lattice parameters *a* = 6.173 Å and *c* = 12.438 Å. There is limited information available on the structural studies of both In_3_Te_4_ and In_3_S_4_. Zavrazhnov et al. [60] reported that the crystal structure of In_3_S_4_ adopts a cubic system (point group of *m*3*m*) with an isotropic lattice parameter of 10.736_1_ Å.

### In_2_X_3_ (X = Te, Se, S)

At atmospheric pressure, In_2_Te_3_ exists in 2 polymorphic forms: *α*-In_2_Te_3_ (stable at lower temperatures) and *β*-In_2_Te_3_ (the high-temperature phase) [10,61]. *α*-In_2_Te_3_ has a defective fluorite structure, while *β*-In_2_Te_3_ has a defective zincblende structure (Fig. 3B and C). The transition from *α*-In_2_Te_3_ to *β*-In_2_Te_3_ occurs at temperatures above 733 K. Both *α*-In_2_Te_3_ and *β*-In_2_Te_3_ possess face-centered cubic (FCC) lattice (space group *F*$\bar{4}$3*m*), with lattice parameters of *a* = *b* = *c* = 18.50 Å and *a* = *b* = *c* = 6.16 Å [62], respectively. Due to the presence of vacancies in one-third of the cationic sites, the defect concentration in In_2_Te_3_ reaches 10^21^ cm^−3^. In *α*-In_2_Te_3_, these vacancies are orderly distributed, whereas in *β*-In_2_Te_3_, they are randomly distributed [63]. At room temperature, each indium atom in the *α*-In_2_Te_3_ structure is exclusively bonded to 4 Te atoms, arranged in a tetrahedral configuration.

In_2_Se_3_ compounds also exhibit different crystalline phases at different temperatures. Eight distinct crystalline phases have been reported, comprising 3 predominant phases (*α*, *β*, and *γ*) and 5 less frequently observed phases (*δ*, *κ*, *α′*, *β′*, and *γ′*) [64–66]. According to the stacking order, the *α* phase has 2 stacking modes, hexagonal (2H) and rhombohedral (3R), while the *β* phase exists in 3 stacking modes, i.e., triangular (1T), 2H, and 3R. At room temperature, the stable structures of In_2_Se_3_ are *α*(2H) and *α*(3R). The *α*(2H) phase of In_2_Se_3_ crystallizes in the *P*6_3_/*mmc* space group, characterized by lattice parameters *a* = 4.025 Å and *c* = 19.235 Å [47,67]. In contrast, the *α*(3R) polymorph adopts the *R*3*m* space group with the lattice parameters of *a* = 4.026 Å and *c* = 28.750 Å [68]. Both *α*(3R)-In_2_Se_3_ and *α*(2H)-In_2_Se_3_ exhibit a layered structure composed of quintuple Se–In–Se–In–Se layer blocks arranged in an ABBCA sequence, with van der Waals forces bonding the interlayers [69]. Each layer comprises both tetrahedrally and octahedrally coordinated indium atoms, as illustrated in Fig. 3D. The 2H and 3R structures show different stacking arrangements. Unlike the “zigzag” pattern characteristic of the 2H structure, the layers in the 3R structure are distinguished solely by a translation in the *ab* plane [68]. The exploration of 3R structure began with Osamura et al. in 1966 [70], who first proposed a model featuring InSe_4_ tetrahedra and InSe_6_ octahedra, but with questionable details of interatomic distances. Later, another structure with In atoms coordinated solely in tetrahedral arrangements was suggested by Ye et al. [71], but with highly questionable singly coordinated Se atoms. In 2015, Debbichi et al. [72] introduced a new model [Fig. 3D(i)] through quantum-chemical calculations, which was finally confirmed by Zhou et al. [73] in 2017 through electron microscopy. Compared to purely octahedral coordination, the mixed coordination in 3R is more conducive to the stability for both covalent and ionic contributions [68].

In_2_S_3_ exhibits 3 different crystalline phases: *α*-type (cubic structure between 717 and 1,049 K), *β*-type (tetragonal structure below 717 K), and *γ*-type (trigonal structure above 1,049 K) [74] (Fig. 3E to G). The low-temperature phase *β*-In_2_S_3_ can be described with a defective spinel-like structure with orderly arranged indium vacancies. It consists of octahedral and tetrahedral In atoms, bonded exclusively to S atoms. The space group is *I*4_1_/*amd* (no. 141), and the lattice parameters are *a* = 7.6231(4) Å and *c* = 32.358(3) Å. The space group of *α*-In_2_S_3_ is *Fd*$\bar{3}$*m* (no. 227), with a lattice parameter of 10.8315(2) Å. *α*-In_2_S_3_ shows a random distribution of indium vacancies over all tetrahedral sites. The high-temperature phase *γ*-In_2_S_3_ adopts a layered structure with the space group of *P*$\bar{3}$*m*1 (no. 164) and lattice parameters of *a* = 3.8656(2) Å and *c* = 9.1569(5) Å. In *γ*-In_2_S_3_, the S–In–S–In–S slabs consist of close-packed sulfur layers, with indium atoms having octahedral coordination.

### In_2_Te_5_

In_2_Te_5_ has a layered structure with zigzag layers (as shown in Fig. 3J), which are formed by edge-sharing In–Te tetrahedra and connected Te–Te bonds [75]. There are 2 distinct crystalline forms of In_2_Te_5_, characterized by space groups *Cc* and *C*2/*c* [76], respectively. These 2 forms differ in their stacking sequence (Fig. 3H and I). The structure with space group *Cc* has lattice parameters of *a* = 4.39 Å, *b* = 16.39 Å, and *c* = 13.52 Å, and the structure belonging to the space group *C*2/*c* exhibits lattice parameters of *a* = 16.66 Å, *b* = 4.36 Å, and *c* = 41.34 Å [77].

For binary indium-based chalcogenides, a comprehensive summary of the various materials typically involves the conventional In–X bonding, which serves as the cornerstone of these structures. Nevertheless, we have noted that most indium-based chalcogenides feature unique structural motifs, such as In–In bonding, Se–Se bonding, planar-coordinated Te–Te bonding, and In3 chain. Additionally, the mixed valence states of indium, for example, In^+^, In^2+^, and In^3+^, importantly contribute to the structural stability, electrical transport, and thermal transport properties of these materials. The interplay between different types of bonding and valence states may give rise to the diverse and complex behaviors observed in these materials, making them a subject of great interest for further research and application.

## Electronic and Phonon Band Structures

To investigate the role of the aforementioned unconventional bonds, a consistent computational approach was adopted for the calculation of band structures, phonon dispersions, and related orbital and vibrational properties of these binary InX compounds. The Vienna ab initio simulation package [78] was utilized for all ab initio calculations based on density functional theory, employing the projector-augmented wave method [79]. The revised Perdew–Burke–Ernzerhof for solids (PBEsol) functional [80] was employed. Considering the layered crystal structure in most systems, a van der Waals correction was implemented using the DFT-D3 method [81]. The cutoff of plane-wave energy was 520 eV. The energy convergence criterion used for electronic band structure was 10^−4^ eV. Structural optimization was conducted until residual forces on all atoms fell below 0.01 eV/Å, ensuring equilibrium geometries for both lattice constants and atomic coordinates. The electronic structures were analyzed employing the modified Becke–Johnson method (mBJ) [82,83] to more accurately evaluate the band gaps. Bonding analysis was performed via the band-resolved projected crystal orbital Hamilton population (pCOHP) method [84], using the Local Orbital Basis Suite Towards Electronic-Structure Reconstruction package [85]. For lattice dynamics, phonon dispersions were computed via the PBEsol functional within the PHONOPY code [86], employing stringent convergence thresholds of 5 × 10^−8^ eV for energies and 10^−5^ eV/Å for atomic forces. The VibCrystal tool was utilized to visualize the lattice vibrations [87].

### Electronic band structure

The space groups and lattice constants (with calculated results presented in unit cell form for direct comparison with experimental data) of these calculated room-temperature phases are listed, and the computed band gaps are compared with experimental data in Table. Computational results exhibit good consistency with experimental band gap values across most systems. Additionally, Heyd–Scuseria–Ernzerhof (HSE06) [88] hybrid functionals were implemented for band structure analysis for some systems (as shown in Figs. S1 to S3). For certain systems, such as *α*(3R)-In_2_Se_3_, the band gap obtained using the mBJ method demonstrates better alignment with experimental values than that using the HSE06 functional (Fig. S2). Considering the computational cost and accuracy for systems with more than 20 atoms, the results derived from the mBJ approach were ultimately adopted. The specific band structures will be elucidated in Indium tellurides, Indium selenides, and Indium sulfides. Concurrently, we focus on the comparative bonding analysis of indium selenides as a representative example.

**Table.** Calculated lattice parameters and band gaps of binary indium-based chalcogenides

Type	Formula	*N* atoms in primitive cell	Crystal structure	Calculated lattice parameters (Å)			Band gap (eV)	
				*a*	*b*	*c*	This work	Experiment
Indium telluride	In_4_Te_3_	28	*Pnnm* (no. 58)	12.050	14.161	4.411	0.285	0.29 [89]
	InTe	8	*I*4*/mcm* (no. 140)	7.593	7.593	6.931	0	0.22 [92]
	*α*-In_2_Te_3_	45	*F*$\bar{4}$3*m* (no. 216)	17.521	17.521	17.521	0.318	-
	In_2_Te_5_	14	*Cc* (no. 9)	4.094	15.087	12.590	0.288	-
	In_2_Te_5_	42	*C*2*/c* (no. 15)	15.151	4.138	37.607	0.399	0.21–0.27 [77,160]
Indium selenide	In_4_Se_3_	28	*Pnnm* (no. 58)	11.688	13.985	4.022	0.221	0.42 [89]
	*β*-InSe	8	*P*6_3_*/mmc* (no. 194)	3.816	3.816	15.865	1.269	1.2 [106]
	*γ*-InSe	4	*R*3*m* (no. 160)	3.871	3.871	23.111	1.222	-
	In_6_Se_7_	26	*P*2_1_/*m* (no. 11)	9.031	3.876	16.642	0	0.54 [161]
	*α*(3R)-In_2_Se_3_	5	*R*3*m* (no. 160)	3.937	3.937	26.447	1.450	1.42 [124]
Indium sulfide	InS	8	*Pnnm* (no. 58)	3.834	10.730	3.829	0.421	1.9 [31]
	In_6_S_7_	26	*P*2_1_/*m* (no. 11)	8.746	3.730	16.087	0.031	0.64–0.75 [162]
	*β*-In_2_S_3_	40	*I*4_1_/*amd* (no. 141)	7.498	7.498	30.172	2.346	2.0–2.2 [163]

#### Indium tellurides

**Fig. 4.**
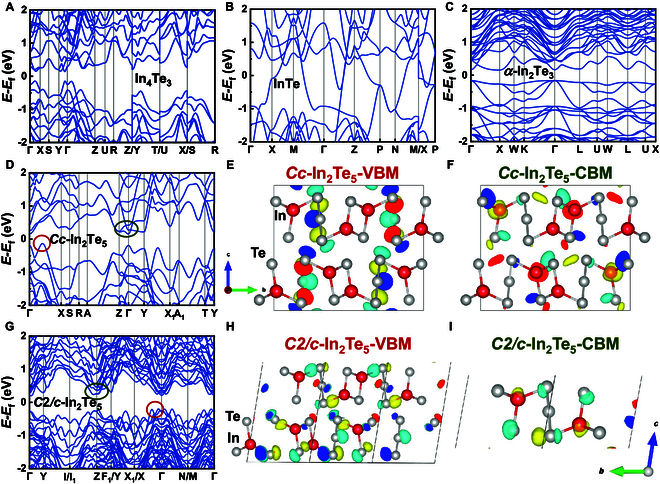
Calculated band structures of (A) In_4_Te_3_, (B) InTe, (C) *α*-In_2_Te_3_, (D) *Cc*, and (G) *C*2/*c* phases of In_2_Te_5_, and the Fermi levels are set to zero. The corresponding wavefunctions of (E) *Cc*-In_2_Te_5_ at VBM, (F) *Cc*-In_2_Te_5_ at CBM, (H) *C2/c*-In_2_Te_5_ at VBM, and (I) *C2/c*-In_2_Te_5_ at CBM. Colored circles mark the positions of wavefunctions.

Figure 4 displays the calculated band structures of In_4_Te_3_, InTe, *α*-In_2_Te_3_, *Cc*, and *C*2/*c* phases of In_2_Te_5_, all computed using the mBJ potential based on primitive cells. Selected wavefunctions associated with the special bonding systems under investigation are also included. Among the 5 indium telluride compounds, 4 (excluding InTe) exhibit indirect band gaps ranging from 0.2 to 0.3 eV.

For In_4_Te_3_, our calculation yielded an indirect band gap of 0.285 eV, close to the experimental data (0.29 eV [89]). The valence band maximum (VBM) occurs along the **Γ**–**X** direction, while the conduction band minimum (CBM) is along the **Y**–**Γ** direction. Observations reveal the formation of a camel’s back-like structure near the **Γ** point, in close proximity to the VBM. This feature forms a pocket-like configuration. Each pocket-like structure exhibit pronounced contributions to the power factor (PF) [90]. More pockets in proximity to the Fermi surface are advantageous for enhancing the Seebeck coefficient, as the enhanced density of states (DOS) near the Fermi level [91].

For InTe, it does not exhibit a band gap, which differs from the small band gaps (0.06 to 0.3 eV) reported in other studies [30,32,92]. This discrepancy may be due to the incorporation of van der Waals correction that leads to a reduced lattice constant, and InTe does not exhibit the pronounced layered properties. After excluding the effects of van der Waals interaction, we employed the advanced r^2^SCAN functional [93] to relax the structure and calculate the electronic structure of InTe, revealing a bandgap of approximately 0.16 eV (see Fig. S4). The VBM resides at the **M** point (−0.5, 0.5, 0.5), while the CBM is located along the **X**–**P** path, in agreement with previously reported results [30,45].

For *α*-In_2_Te_3_, the VBM is along the **K**–**Γ** direction, while the CBM is located at **X** (0.5, 0, 0.5) point, with an indirect band gap of 0.318 eV. The valence band edge exhibits little dispersion, leading to a large effective mass that impedes electrical transport. Reports on this phase are limited.

For *Cc*-In_2_Te_5_, the VBM is along the **Γ**–**X** direction (as indicated by the red marking in Fig. 4D), while the CBM is located at **Γ**, with an indirect band gap of 0.288 eV (dark green circle). For *C*2*/c*-In_2_Te_5_, both the VBM and CBM are not located at the high symmetry point, with an indirect band gap of 0.399 eV. It is worth noting that both phases exhibit notable band edge dispersion, particularly the M-shaped VBM in the *C*2*/c* phase, which is conducive to the electrical transport properties. Analysis of the wavefunctions at the band edge (Fig. 4E, F, H, and I) reveals highly similar compositions in these 2 phases. Notably, both show significant contributions from planar-coordinated Te (*p*-orbital) at VBM, while the CBM is mainly composed of conventional In (*s*-like orbital) and Te–*p* orbital states.

#### Indium selenides

**Fig. 5.**
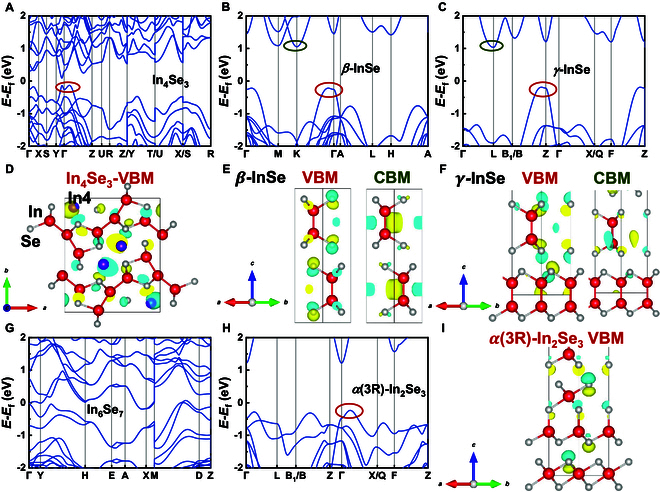
Calculated band structures of (A) In_4_Se_3_, (B) *β*-InSe, (C) *γ*-InSe, (G) In_6_Se_7_, and (H) *α*(3R)-In_2_Se_3_, and the Fermi levels are set to zero. The corresponding wavefunctions of (D) In_4_Se_3_ at VBM, (E) *β*-InSe at VBM and CBM, (F) *γ*-InSe at VBM and CBM, and (I) *α*(3R)-In_2_Se_3_ at VBM. Colored circles mark the positions of wavefunctions.

Indium selenides are a class of materials with varying compositions and crystal structures, significantly impacting their physical properties. Elucidating the electronic structural nuances of indium selenides may facilitate the exploration of potential methodologies for the enhancement of their TE performance. The calculated band structures and partial wavefunctions of In–Se compounds are shown in Fig. 5.

Similar to In_4_Te_3_, In_4_Se_3_ also has shaper camel’ s back-like VBM, and CBM is along the **Y**–**Γ** direction, exhibiting an indirect band gap of 0.221 eV. As shown in Fig. 5D, wavefunction analysis reveals that the VBM of In_4_Se_3_ primarily consists of In–*s* orbitals (particularly from the isolated In4 atoms, represented by purple spheres in the figure) and Se–*p* orbitals, consistent with the findings reported by Losovyj et al. [94]. For InSe, *β* and *γ* phases show comparable indirect band gaps. Despite belonging to distinct space groups and having different high-symmetry points, the valence and conduction band edges of these compounds exhibit analogous shapes. The wavefunction distributions (Fig. 5E and F) further demonstrate similar band-edge compositions between these 2 phases. The VBM in both cases predominantly arises from Se–*p* orbitals with potential interlayer Se interactions, while the CBM likely originates from In–In bonding interactions combined with Se–*p* orbital contributions. These features will be further analyzed through subsequent pCOHP calculations. This similarity arises from the identical structural motifs within their individual layers, differentiated solely by the stacking sequences, as previously described in the structural context.

In_6_Se_7_ has no band gap according to our calculation in both mBJ and HSE06 potential (see Fig. S2D). For *α*(3R)-In_2_Se_3_, its VBM is located along **Γ**–**X**, with the CBM situated at **Γ** point, featuring an indirect gap of 1.450 eV. As illustrated in Fig. 5I, the VBM of *α*(3R)-In_2_Se_3_ comprises Se–*p* orbital characteristics. It has the same space group as *γ*-InSe, which is equivalent to adding an additional Se layer on the basis of the latter. Compared with *γ*-InSe, the band structure has undergone changes, particularly along the segments **L**–**B**_1_|**B**–**Z** and **Γ**–**X**|**Q**–**F**, resulting in the emergence of a multi-valley valence band edge. This may be attributed to the variations in the unusual Se–Se and In–In bonding within the material, which will be specifically analyzed later.

**Fig. 6.**
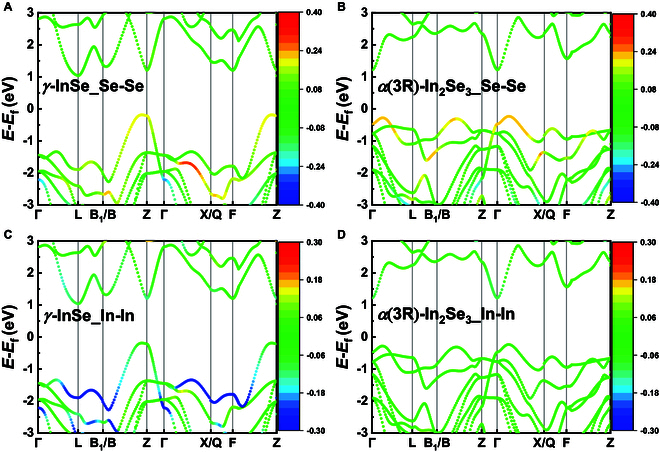
Band-resolved pCOHP of (A) Se–Se in *γ*-InSe, (B) Se–Se in *α*(3R)-In_2_Se_3_, (C) In–In in *γ*-InSe, and (D) In–In in *α*(3R)-In_2_Se_3_. The Fermi level is set to zero. Red represents anti-bonding, and blue represents bonding.

To further elucidate the influence of bonding beyond the conventional indium–chalcogenide interactions within binary indium chalcogenides, a case study of indium selenide compounds was carried out. Specifically, *γ*-InSe and *α*(3R)-In_2_Se_3_ were selected due to their appropriate system size for chemical bonding analysis. The results of band-resolved pCOHP calculations are shown in Fig. 6, where red and blue represent anti-bonding and bonding, respectively. According to Fig. 6A and B, the VBM of both *γ*-InSe and *α*(3R)-In_2_Se_3_ exhibits anti-bonding interactions stemming from interlayer Se–Se interactions (consistent with the results of the VBM orbital in Fig. 5F and I), with the latter exhibiting a more pronounced effect. The multi-valley feature of the VBM in In_2_Se_3_ is likely attributed to the shortened interlayer Se–Se bond length (3.176 Å in the In_2_Se_3_ phase, as opposed to 3.262 Å in the InSe phase). The anti-bonding interaction of Se–Se increases the band energy along **Γ**–**L**, **B**–**Z**, and **Γ**–**X**, which leads to a more effective convergence of the band at the VBM [95]. In addition to the Se–Se interactions, our investigation reveals that the In–In bonding within InSe also exerts certain effects in valance band, characterized by the bonding interactions that arise from the proximity of In–In atoms (Fig. 6C). In contrast, within the In_2_Se_3_ phase, the absence of neighboring In–In layers excludes the presence of such interaction (see almost green area in Fig. 6D). Consistent with the previous wavefunction result, the cyan lines at the **L** point near 1 eV in Fig. 6C also validate the minimal In–In bonding interactions at the CBM of InSe.

#### Indium sulfides

**Fig. 7.**
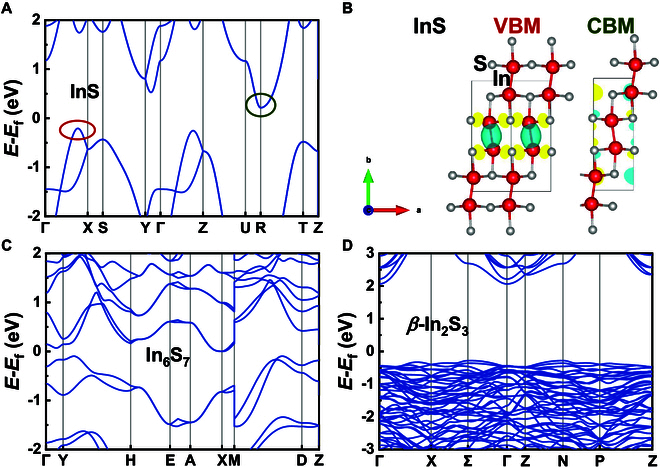
Calculated band structures of (A) InS, (C) In_6_S_7_, and (D) *β*-In_2_S_3_, and the Fermi levels are set to zero. The corresponding wavefunctions of (B) InS at VBM and CBM. Colored circles mark the positions of wavefunctions.

The calculated band structures of InS, In_6_S_7_, and *β*-In_2_S_3_ in the mBJ potential are shown in Fig. 7. For InS, the VBM lies along the **Γ**–**X** direction, while the CBM is located at the **R** point (0.5, 0.5, 0.5), with an indirect band gap of 0.421 eV. As shown in Fig. 7B, the orbital compositions at the band edge of InS show clear features: The VBM is dominated by bonding interactions between In atoms and dumbbell-shaped orbitals localized on S atoms, while the CBM encompasses spherical In orbitals combined with contributions from S–*p* orbitals. For In_6_S_7_, both the VBM and CBM are located away from high-symmetry points. It exhibits a narrow indirect band gap of 0.031 eV, and its band structure is similar to that of In_6_Se_7_. In contrast, *β*-In_2_S_3_ reveals an indirect gap of 2.346 eV, with dispersionless VBM and a **Γ**-point CBM. Owing to its suitable wide band gap, *β*-In_2_S_3_ thin films have been utilized as an efficient electron transport layer for perovskite solar cells [96].

### Phonon band structure

**Fig. 8.**
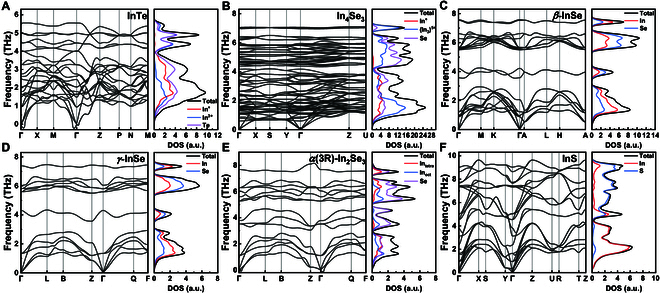
Calculated phonon band structures with total and partial phonon DOS of (A) InTe, (B) In_4_Se_3_, (C) *β*-InSe, (D) *γ*-InSe, (E) *α*(3R)-In_2_Se_3_, and (F) InS.

The computational phonon dispersions with total and partial phonon DOS for several systems, chosen for their fewer atoms in the primitive cells, are shown in Fig. 8. Note that InTe exhibits imaginary frequencies (~−0.2 THz) near the **Γ** point (see Fig. 8A), which is consistent with the computational results of other studies [97], unless under a pressure of 3 GPa [32,98]. The In^3+^ and Te atoms are the primary contributing factors of the low-frequency phonons. It is noteworthy that the special In^+^ in InTe (red line in Fig. 8A) is active in the mid-frequency optical branch (with frequencies of 1.78 and 2.04 THz, and the specific vibrational modes at **Γ** point can be seen in Fig. S5). Little interaction of In^+^ with other atoms was observed, which indicates the weakly bound “rattling” In^+^ atoms in the structure. This characteristic is expected to increase the anharmonicity of the system, thereby enhancing the Grüneisen parameter, which effectively reduces the lattice thermal conductivity [32,97].

Regarding In_4_Se_3_, its small phonon group velocity can be estimated from the flat dispersion of the phonon dispersion (see Fig. 8B). Typically, the lattice thermal conductivity is predominantly attributed to the acoustic branch. The relatively small slope of the acoustic phonon branch in this system suggests a low phonon group velocity, which serves as an indicator of its low lattice thermal conductivity. Similar to InTe, isolated In^+^ atom is found in In_4_Se_3_ (red line in Fig. 8B) and is active in the low- to mid-frequency region of the phonon dispersion, e.g., 2.08 THz at **Γ** point (Fig. S6). Furthermore, our analysis reveals the presence of In–In interactions within the high-frequency optical branch. Notably, the highest optical mode at **Γ** point, with a frequency of 7.04 THz, demonstrates a relative motion confined to the (In_3_)^5+^ chain (Fig. S6). The high-frequency stretching and low-frequency collective motion of the (In_3_)^5+^ chain confirm the strong chemical bonding between the In–In atoms in the (In_3_)^5+^ chain [99,100].

Due to the difference in their layer stacking, the phonon dispersion of *β*-InSe and *γ*-InSe exhibits distinct features (see Fig. 8C and D); however, certain characteristics, such as the gap between phonon branches, remain similar. In contrast to the bulk InTe, both InSe materials display distinct layered vibrational modes at **Γ** point, encompassing intra- and interlayered motion. In *β*-InSe, the low-frequency optical phonons correspond to motions within the *ab* plane (Fig. S7). For example, in the phonon mode with a frequency of 0.96 THz, the Se–In–In–Se layers undergo relative shear motion against each another, resembling 2 interlayer slips. Such mode, with energies in close proximity to acoustic phonons, can interact strongly with them. This interaction leads to strong phonon–phonon scattering and an enhancement of anharmonicity, thereby resulting in a low thermal conductivity [101]. In the slightly higher mode at 1.23 THz, every 2 layers of In–Se move in opposite directions within the *ab* plane, corresponding to intralayer shear. At 3.54 THz, every 2 layers of In–Se move in opposite directions along the *c* axis. In the highest optical mode (7.52 THz), adjacent atomic layers (whether In or Se layers) vibrate toward each other along the *c* axis. Similarly, in the *γ* phase, the low-frequency region (1.34 THz) exhibits intralayer sliding at **Γ** point, while the mid-frequency region (4.3 THz) and the highest mode (7.38 THz) involve vibrations along the *c* axis (Fig. S8).

In *α*(3R)-In_2_Se_3_, a tiny imaginary mode appears around the zone center (see Fig. 8E). This mode cannot be related to any structural transition, as its frequency is found to be negligible. All phonons at **Γ** point below 3.6 THz correspond to motions within the *ab* plane (Fig. S9). Specifically, the phonon at 3.52 THz, which exhibits relatively flat dispersion, represents the vibration of the octahedral In–Se atoms (also blue line in DOS of Fig. 8E). The branch at 3.78 THz encompasses the relative motion between tetrahedral Se and octahedral Se. The concerted motion of these 2 types of Se–Se interactions occurs at 5.60 THz. The highest optical mode at **Γ** point (7.68 THz) is the reverse motion between 2 neighboring atomic layers along the *c* axis, except octahedral In. This is also reflected in the phonon DOS, where octahedral In shows no contribution at high frequencies.

As for InS (Fig. 8F and Fig. S10), phonons at **Γ** point below 2.25 THz are associated with motions confined to the *ab* plane, while those between 2.37 and 3.90 THz correspond to breathing-mode vibrations along the *c* axis, such as the 3.13-THz mode shown in Fig. S10. The pendular motion of the S atoms at **Γ** point predominantly governs the phonon modes between 4.50 and 7.50 THz, including the 7.34-THz mode shown in Fig. S10.

In summary, the presence of unconventional bonds (e.g., In–In, Te–Te, and Se–Se) in In–X compounds may influence the electronic structure near the band edges. Phonon dispersions, DOS, and their corresponding vibrational modes further highlight the effects of unconventional bonds on the lattice vibrations. Meanwhile, the isolated In^+^ atom vibrating at the low-to-mid frequency enhances the anharmonicity of the system, and the softened low-frequency optical branch, with energies similar to those of acoustic phonons, increases phonon–phonon scattering. These features both serve as indications of low thermal conductivity. However, the underlying mechanisms contributing to the low thermal conductivity of these materials still require further investigation. Our computational results provide microscopic insights into the role of unconventional bonds in shaping the electronic and phononic transport characteristics of In–X compounds.

## Experimental Strategies for Optimizing TE Properties

**Fig. 9.**
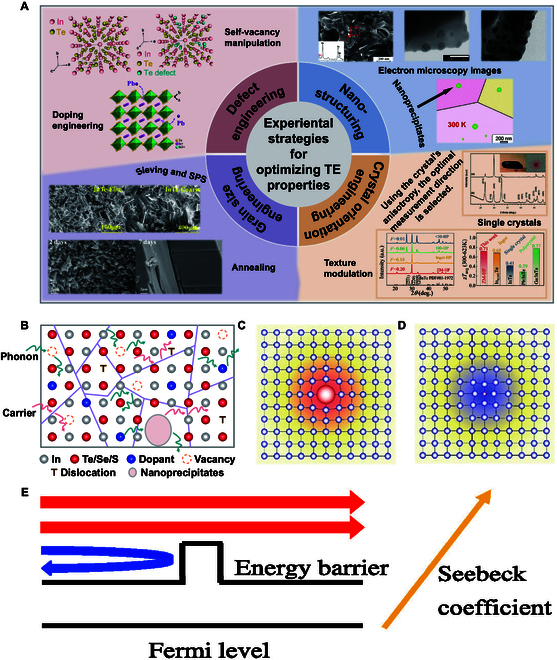
(A) Experimental strategies for optimizing TE properties through defect engineering, crystal orientation engineering, and nanostructure and grain size engineering. Upper-left figure: Reproduced with permission [154]. Copyright 2020 American Chemical Society. Reproduced with permission [105]. Copyright 2023 by Zhou et al. Lower-left figure: Reproduced with permission [105]. Copyright 2023 by Zhou et al. Reproduced with permission [92]. Copyright 2020 Elsevier B.V. Upper-right figure: Reproduced with permission [117]. Copyright 2013 The American Ceramic Society. Reproduced with permission [103]. Copyright 2013 Wiley-VCH Verlag GmbH & Co. KGaA, Weinheim. Reproduced with permission [129]. Copyright 2019 the Royal Society of Chemistry. Lower-right figure: Reproduced with permission [108]. Copyright 2021 the Royal Society of Chemistry. Reproduced with permission [128]. Copyright 2023 the Royal Society of Chemistry. (B) Schematic representation of the behavior of carriers and phonons. Reproduced with permission [155]. Copyright Science China Press 2024. (C and D) Schematic representation of local distortions caused by solid solution of solute atoms with different radii. Reproduced with permission [156]. Copyright 2023 Wiley-VCH GmbH. (E) Schematic representation of the energy filtering effect. Reproduced with permission [157]. Copyright 2021 Chinese Chemical Society.

At the experimental level, we have summarized the TE optimization strategies employed in recent years for binary indium-based chalcogenides. These approaches can be classified into 4 categories: defect engineering, crystal orientation engineering, nanostructuring, and grain size engineering, as shown in Fig. 9A. These strategies have effectively improved thermal transport, electrical transport, or both simultaneously, leading to a significant enhancement in the *zT* value. For thermal transport, point defects (e.g., vacancies [32,42] and impurity atoms [102]), line defects (dislocations [30]), and nanoprecipitates [103,104] can effectively scatter phonons, thereby reducing the lattice thermal conductivity (Fig. 9B). Doping, a common approach to generate point defects, enhances phonon scattering through lattice distortions caused by solute atoms with mismatched ionic radii (Fig. 9C and D). For electrical transport, the carrier concentration can be controlled by adjusting the doping level. For example, Zhou et al. [105] reduced the carrier concentration by introducing the donor impurity Pb at the In^+^ site in p-type InTe materials. In contrast, Zhai et al. [106] increased the carrier concentration by doping Sn in *β*-InSe, which also resulted in a narrowed band gap. Additionally, doping can induce energy filtering effects (Fig. 9E). For instance, Luo et al. [107] found that the carrier concentration in Pb/Cu co-doped samples was lower than that in Pb single-doped samples, while the carrier mobility exhibited an opposite trend. This behavior was primarily attributed to the higher work function of Cu (4.65 eV) compared to In_4_Se_3_ (4.30 eV), forming an energy barrier that filters out low-energy electrons and resulting in a higher *S* at elevated temperatures in the co-doped samples. In addition, the temperature dependence of electrical conductivity can be tuned by increasing the grain size [30,92], and leveraging the intrinsic anisotropy of the material may optimize thermal or electrical properties [42,108], thereby enhancing TE performance. These methods, either individually or in combination, have been successfully implemented in indium-based chalcogenide compounds. Accordingly, this section reviews recent experimental advances in these modulation strategies and examines the underlying physical mechanisms.

### Defect engineering

#### Self-vacancy manipulation

In order to optimize the TE performance, self-vacancy is widely adopted as an effective strategy in InTe and In_4_Se_3_ systems. For p- and n-type semiconductors, self-vacancy has the unique advantage of tailoring the carrier concentration and phonon scattering while minimizing the impact on crystal structure.

**Fig. 10.**
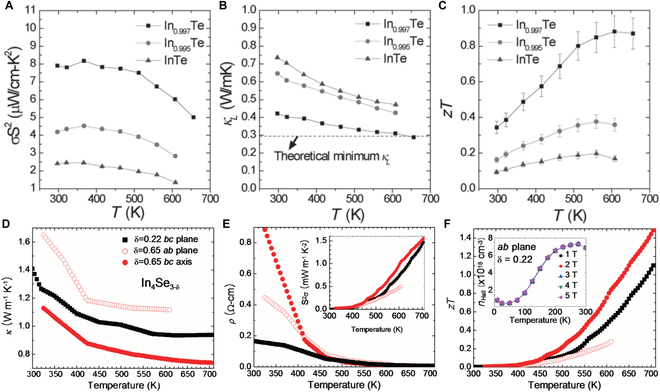
Temperature-dependent (A) PF, (B) *κ*_L_, and (C) *zT* (with 10% error bars) of In_1−*x*_Te samples. Reproduced with permission [32]. Copyright 2016 Wiley-VCH Verlag GmbH & Co. KGaA, Weinheim. TE properties of Se-deficient In_4_Se_3-_*_δ_* crystals. (D) Temperature-dependent thermal conductivity and (E) temperature-dependent electrical resistivity and PF (inset). (F) Temperature-dependent *zT* and Hall carrier concentration *n*_Hall_ (inset). Reproduced with permission [42]. Copyright 2009 Macmillan Publishers Limited.

For p-type InTe, Jana et al. [32] successfully enhanced the PF (PF = *σS^2^*) and reduced the lattice thermal conductivity by creating In deficiency (Fig. 10A to C). As a result, the *zT* value of the In_0.997_Te sample was significantly enhanced, peaking at approximately 0.9 at 600 K. This represents a notable improvement compared to that of undoped InTe. The increase in the PF was mainly driven by the improved carrier mobility and carrier concentration. The large reduction in lattice thermal conductivity was expected to arise from phonon scattering caused by vacancy-type point defects. Similarly, the researchers also experimented with Te deficiency [109]. The *zT* values of InTe_1−*δ*_ were substantially enhanced within the mid-temperature range (*T* ≤ 500 K), due to a decrease in lattice thermal conductivity and an increase in the PF.

The introduction of Se deficiency into n-type In_4_Se_3_ has been a well-established strategy. In 2009, Rhyee et al. [42] successfully synthesized 2 Se-deficient crystals (In_4_Se_2.78_ and In_4_Se_2.35_). The In_4_Se_2.35_ crystal exhibited an unusually high *zT* value of 1.48 along the *b*–*c* plane at 705 K, surpassing the *zT* value of 1.1 in the In_4_Se_2.78_ crystal along the same plane at the same temperature. This *zT* enhancement primarily resulted from greater Se deficiency in the In_4_Se_2.35_ crystal, which amplified phonon scattering, thereby leading to a significant reduction in thermal conductivity (see Fig. 10D to F). Later, Rhyee et al. [110] reported that increasing the Se deficiency (*x*) of In_4_Se_3−*x*_ from 0.02 to 0.05 resulted in an enhancement of *zT* from ~0.40 to 0.63 at 710 K, as there is a reduction in the band gap and an increase in the PF. Moreover, the thermal conductivity was lowered due to the disordered phonon scattering caused by the Se-deficient sites. In addition, Alsharafi et al. [111] found that increasing the Se deficiency in the Pb-doped In_4_Se_3_ polycrystalline samples (In_4_Pb_0.01_Se_3−*x*_, *x* = 0 to 0.1) increased the Hall carrier concentration, which consequently lowered the electrical resistivity. Furthermore, the lattice thermal conductivity was also reduced. Ultimately, the sample with *x* = 0.07 exhibited a peak *zT* of 0.95 at 690 K, outperforming the sample without Se vacancies (*x* = 0) by approximately 15% (*zT* = 0.83).

#### Doping engineering

Doping engineering has become a key approach for optimizing the TE properties. By adopting the doping strategy, the electrical conductivity and Seebeck coefficient of a material can be improved due to the optimization of carrier concentration, and the thermal conductivity can also be reduced because of increasing the number of scattering centers for phonons.

**Fig. 11.**
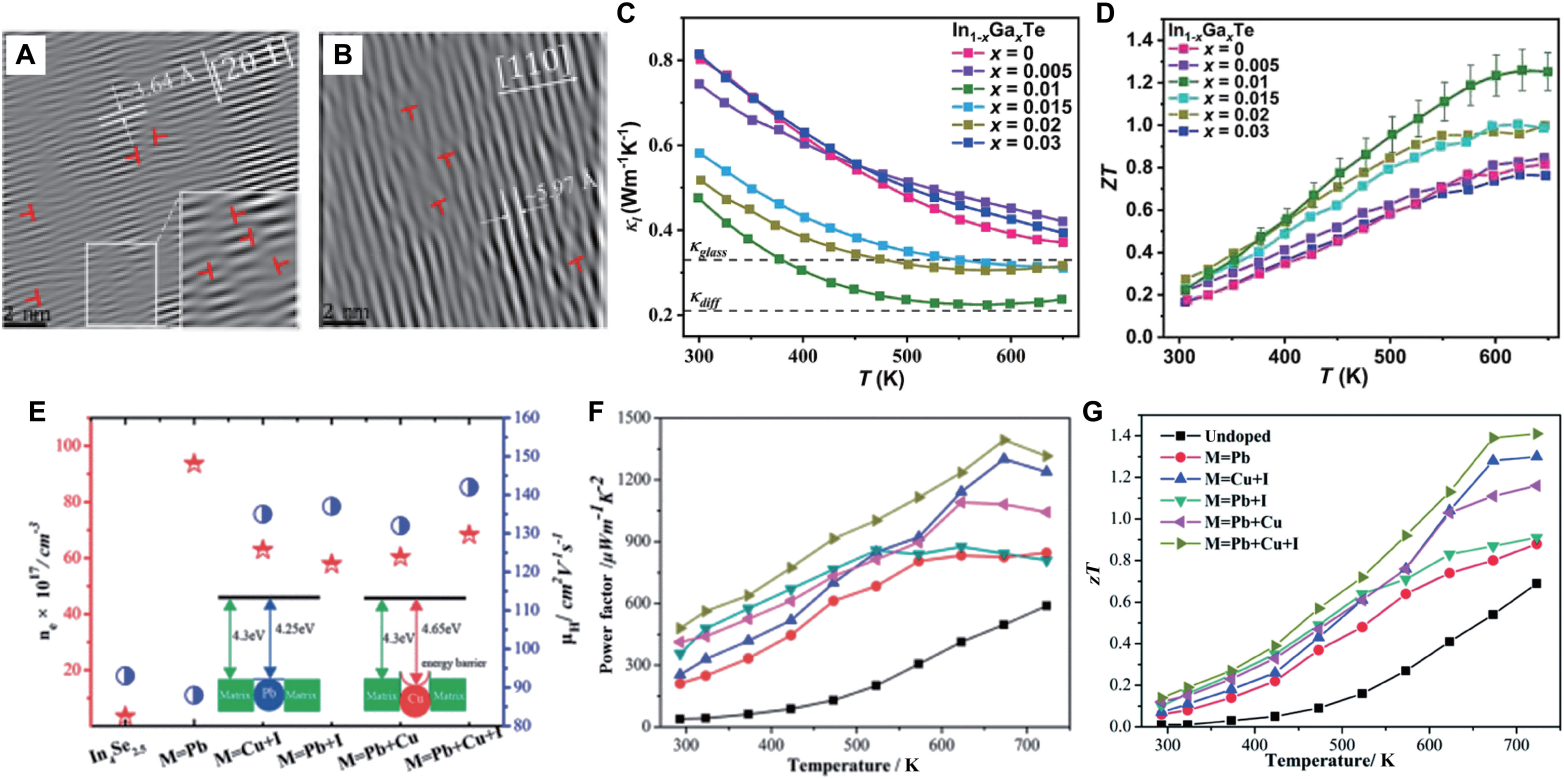
(A and B) Inverse fast Fourier transform patterns of high-resolution TEM lattice images of the ($\bar{2}$0$\bar{1}$) and (110) planes for the In_0.99_Ga_0.01_Te sample. Temperature-dependent (C) *κ*_L_ and (D) *zT* of In_1−*x*_Ga*_x_*Te samples. Reproduced with permission [30]. Copyright 2022 Wiley-VCH GmbH. (E) Carrier concentration and Hall mobility at room temperature, and an inset illustrating the schematic diagram of the energy filtering mechanism. Temperature-dependent (F) PF and (G) *zT* for the undoped/doped In_4_Se_2.5_. Reproduced with permission [107]. Copyright 2022 the Royal Society of Chemistry.

Misra et al. [102] reported that the atomic disorder was induced in the structure by doping Pb into InTe, which increased phonon scattering and decreased the lattice thermal conductivity to 0.22 W m^−1^ K^−1^. Consequently, In_0.999_Pb_0.001_Te achieves a peak *zT* of 1.05 at 790 K. Nevertheless, the use of Pb element raises concerns about environmental and health hazards. Therefore, researchers have been exploring for less toxic alternatives like Cu and Na [112], but generally achieving slightly poorer performance than Pb doping. Until 2022, Li et al. [30] significantly improved the TE properties of InTe through the synergistic optimization of electrical and thermal transport properties. They found that through Ga doping in InTe, the grain boundary scattering (GBS) was purified and weak phonon–electron coupling was induced, thereby improving carrier concentration and mobility. Thus, the PF value was increased to 8.9 μW cm^−1^ K^−2^ at 500 K. Transmission electron microscopy (TEM) characterization results revealed dense in-grain dislocation arrays along with multiple lattice domains with pronounced structural distortions [30] (Fig. 11A and B), which effectively enhanced the scattering of intermediate-frequency phonons, which reduced the lattice thermal conductivity (Fig. 11C). As a result, the In_0.99_Ga_0.01_Te sample achieved a peak *zT* value of 1.2 at 648 K and an average *zT* of 0.8 over the temperature range 300 to 650 K [30], outperforming all other known InTe-based materials (Fig. 11D). Other doping elements such as Bi, Ag, Mn, Sn, and Sb also have been investigated by Song et al. [45] for InTe. Among these doped InTe samples, In_0.99_Sn_0.01_Te exhibited the highest *zT* value of 0.64 at 725 K, corresponding to a >50% improvement over undoped InTe. The TE performance enhancement of these doped InTe samples is primarily due to the reduction in thermal conductivity.

Luo et al. [107] introduced multiple doping of Pb, I, and Cu in In_4_Se_2.5_, which improved the electrical conductivity through simultaneous improvement in carrier concentration and Hall mobility (Fig. 11E). As a result, the highest values of PF and *zT* were achieved in the (Pb + Cu + I)-doped In_4_Se_2.5_ polycrystalline sample from 300 to 723 K (*zT* = 1.4 at 723 K) (Fig. 11F and G). Co-doping of Pb and Sn in In_4_Se_3_ also leads to a larger peak *zT* of 1.4 at 733 K, mainly because Pb and Sn act as effective electron donors, which significantly enhance the carrier concentration by an order of magnitude compared to the undoped sample [103]. The PF value of In_4_Pb_0.01_Sn*_y_*Se_3_ (*y* = 0.03 or *y* = 0.04) is ∼10 μW cm^−1^ K^−2^ at 733 K, representing an enhancement of nearly 80% compared to undoped In_4_Se_3_ [103]. He et al. [113] found that Yb doping at the In site of In_4_Se_3_ leads to a lower thermal conductivity, resulting in a *zT* value of 0.81 at 703 K for the In_3.97_Yb_0.03_Se_3_ and In_3.95_Yb_0.05_Se_3_ samples, which is an improvement of approximately 30% compared to pure In_4_Se_3_ [113]. In addition, other dopants, e.g., Ag [37], Sn [40], CuI [114], and Cl [115] in In_4_Se_3−*x*_, and Sn [116] and Cu [117], have also been demonstrated to improve the TE performance of In_4_Se_3_.

**Fig. 12.**
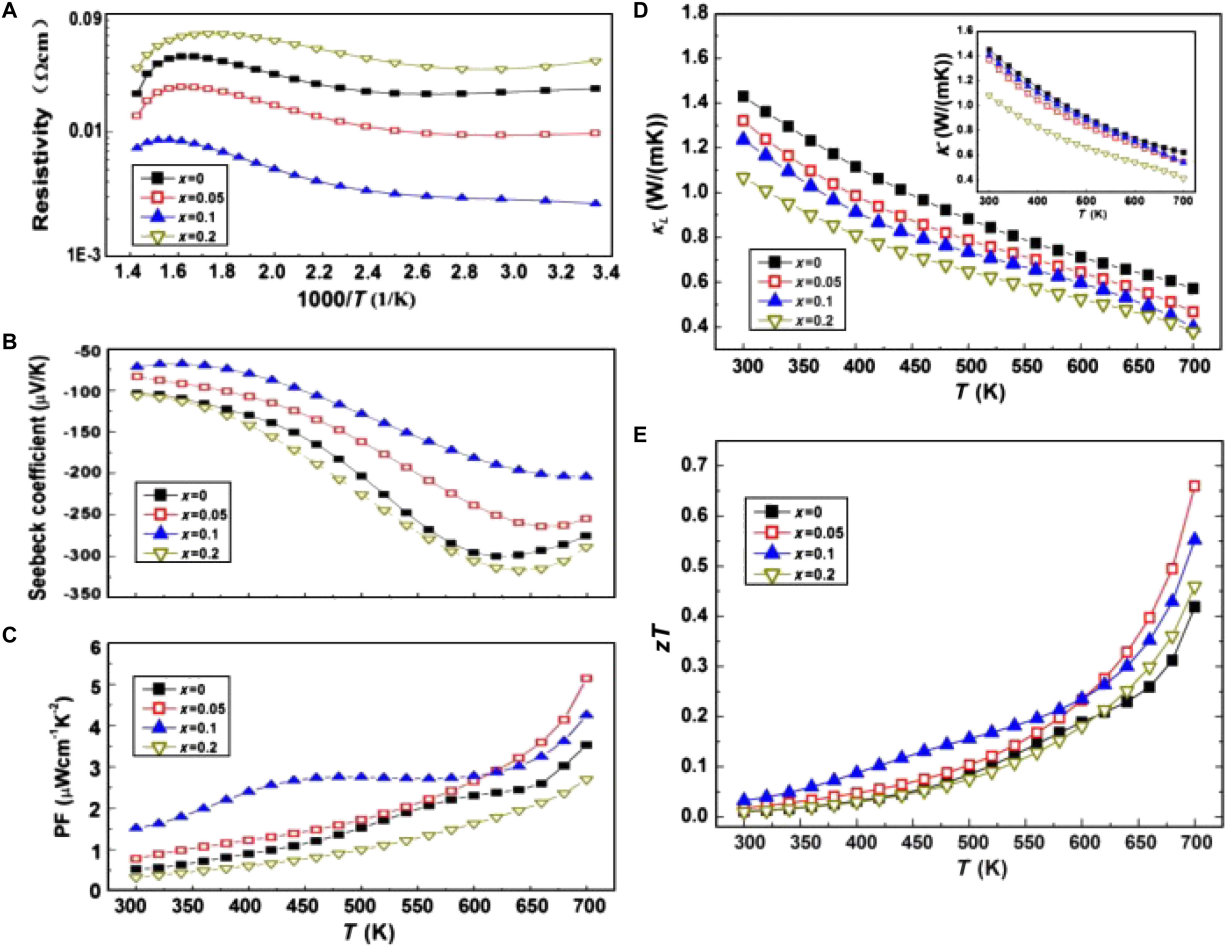
Temperature dependence of (A) *σ*, (B) *S*, (C) PF, (D) *κ*_L_, and (E) *zT* for the In_1.3−*x*_Sn*_x_*Se samples. Reproduced with permission [106]. Copyright 2012 Elsevier B.V.

InSe has a low intrinsic carrier concentration (~10^14^ cm^−3^) due to its relatively large band gap [118], and thus, the electrical conductivity needs to be increased. In 2013, Zhai et al. [106] prepared polycrystalline n-type In_1.3−*x*_Sn*_x_*Se samples (*x* = 0, 0.05, 0.1, and 0.2). By doping Sn into *β*-InSe, a combination of the increased carrier concentration due to a narrowed band gap and the reduced lattice thermal conductivity led to the highest *zT* value of 0.66 at 700 K for the In_1.25_Sn_0.05_Se sample (Fig. 12). This value is 57% higher than that of the undoped sample (*zT* = 0.42). Although doping studies on InSe have continued in recent years, e.g., doping with elements such as Si [118,119] and Cu [120] at the cationic sites, and Cl [121] and Te [122] at the anionic sites, the *zT* value has not been significantly improved.

**Fig. 13.**
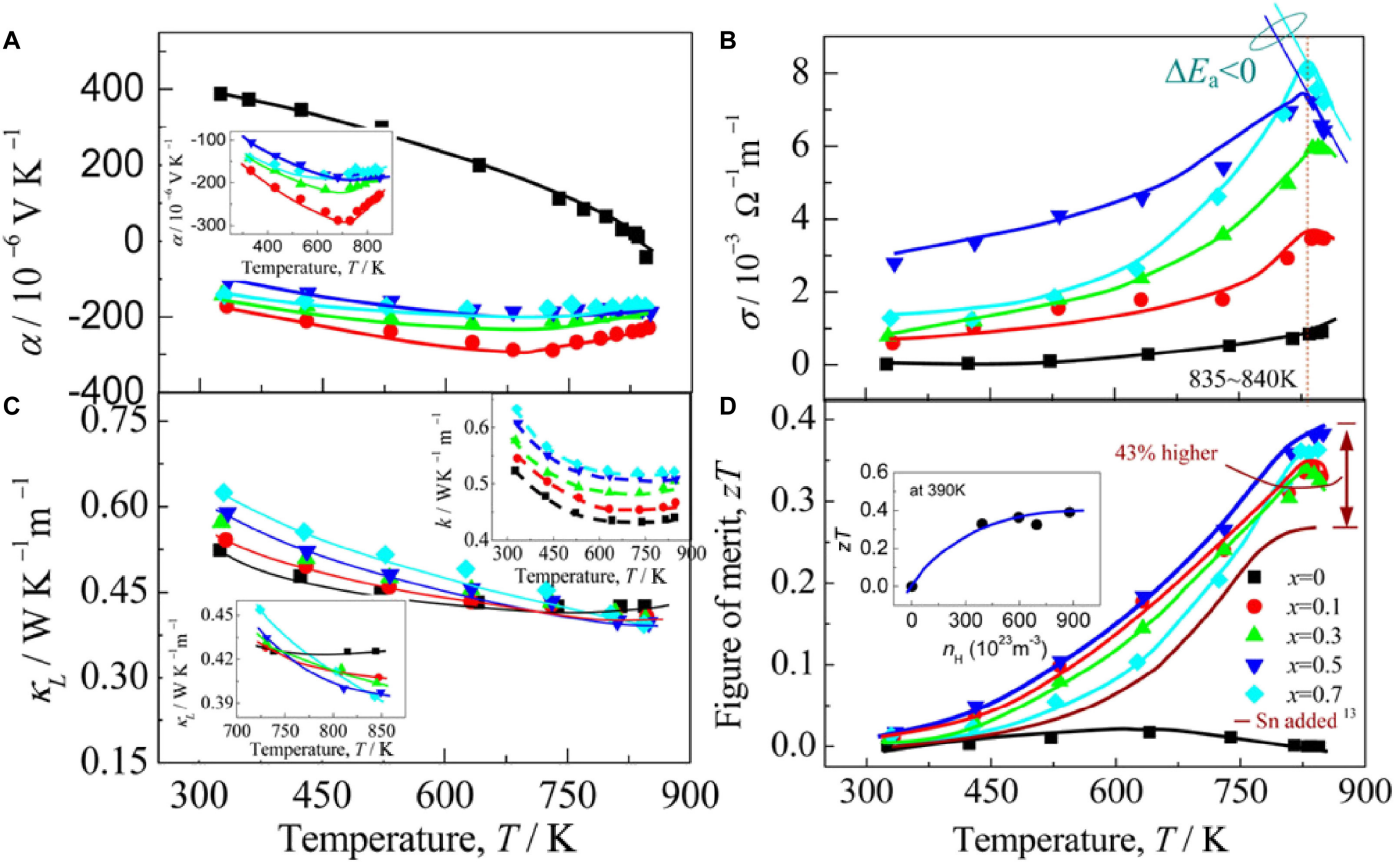
Temperature-dependent (A) *S*, (B) *σ*, where the thermal activation energy Δ*E*_a_ < 0 as *T* ≥ 835 to 840 K, (C) *κ*_L_, where the top right inset is the total thermal conductivity, and (D) *zT*, where the inset shows *zT* versus *n*_H_ (Hall carrier concentration) for the In_6−*x*_Pb*_x_*Se_7_ samples. Reproduced with permission [53]. Copyright 2012 American Chemical Society.

In_6_Se_7_ can undergo a transition from p-type to n-type conduction after Sn or Pb doping [52,53]. In pure In_6_Se_7_, In exhibits multiple valence states (+1, +2, and +3). But Pb^2+^ and Sn^4+^ are inclined to occupy the In^+^ sites, thus resulting in the p–n transition. This p–n transition leads to a significant enhancement in the Hall carrier concentration. Consequently, the Sn-doped sample In_5.9_Sn_0.1_Se_7_ achieved a *zT* value of 0.28 at 833 K, representing an approximately 19-fold enhancement compared to the undoped In_6_Se_7_, which exhibited a *zT* of only 0.015 at 640 K [52]. The In_5.5_Pb_0.5_Se_7_ sample exhibited the highest *zT* value of 0.4 at ~850 K [53] (Fig. 13).

**Fig. 14.**
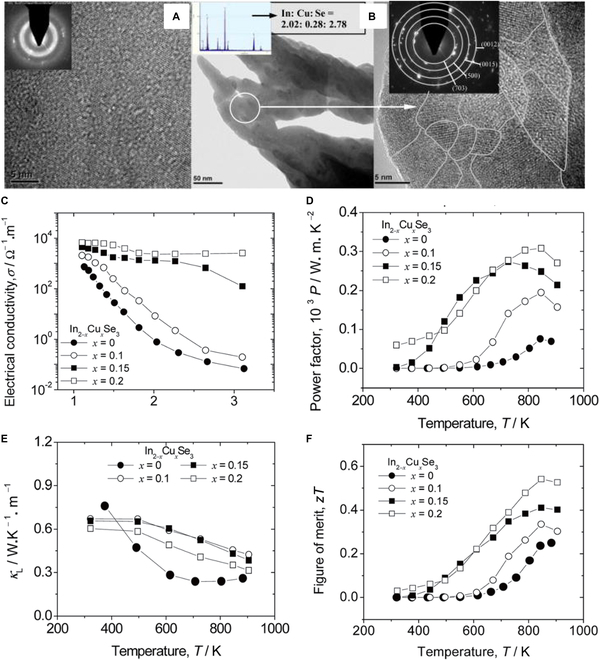
(A) The amorphous-like structure of *α*-In_2_Se_3_ is revealed by the high-resolution TEM image. (B) High-resolution TEM image of In_1.8_Cu_0.2_Se_3_ shows its polycrystalline nature with grain sizes of 5 to 15 nm (gray-lined areas). The upper left inset in (B) shows the energy-dispersive x-ray spectroscopy results with a molar ratio of In:Cu:Se = 2.02:0.28:2.78. The upper right inset in (B) displays the polycrystalline form from electron diffraction. Temperature-dependent (C) *σ*, (D) PF, (E) *κ*_L_, and (F) *zT* values for In_2−*x*_Cu*_x_*Se_3_ bulk materials. Reproduced with permission [123]. Copyright 2011 American Institute of Physics.

Cui et al. [123] studied Cu doping in n-type *α*-In_2_Se_3_, which led to a band gap reduction. Although the transition from an amorphous-like structure to a distinct polycrystalline morphology increased the lattice thermal conductivity (Fig. 14A, B, and E), the PF values increased by 3 to 4 times due to the improved electrical conductivity as a result of the band gap reduction, and eventually achieving a peak *zT* value of approximately 0.55 in the In_1.8_Cu_0.2_Se_3_ sample (*x* = 0.2) at 846 K (Fig. 14). In another study by Cui et al. [124] in 2015, a non-equilibrium fabrication technology (NEFT) was used to achieve Zn/S doping in In_2_Se_3_. This doping improved the electrical conductivity of the material. Zn doping created an anti-site defect Zn_In_ as a donor, resulting in a peak *zT* value of 1.23 (± 0.02) at 916 K in the In_1.99_Zn_0.01_Se_3_ sample. Doping isoelectronic S atoms at the Se site helped avoid the annihilation of donor defects (i.e., V_In_ and In_i_), resulting in a *zT* peak value of 0.67 in the *α*-In_2_S_0.05_Se_2.95_ sample at 923 K. This represents an enhancement of approximately 2.8 times compared to the undoped *α*-In_2_Se_3_, which exhibited a *zT* of only 0.24 [125]. Additionally, doping Si at the In site effectively reduces the thermal conductivity, reaching ~0.35 W m^−1^ K^−1^ for the *β*-phase In_2−*x*_Si*_x_*Se_3_ (*x* = 0.005) at 500 K [126].

For *β*-In_2_S_3_, substituting In atoms with Mg atoms (In_2−*x*_Mg*_x_*S_3_, 0 ≤ *x* ≤ 0.20) can effectively reduce the lattice thermal conductivity [127]. The In_2_S_3_ samples with *x* ≥ 0.30 underwent a phase transition to the cubic *α* phase, which has too high electrical resistivity to obtain meaningful electrical transport properties. For the In_1.95_Mg_0.05_S_3_ sample, a high *zT* value of 0.53 was achieved at 700 K, which represents a 1.4-fold increase compared to the undoped sample.

### Crystal orientation engineering

**Fig. 15.**
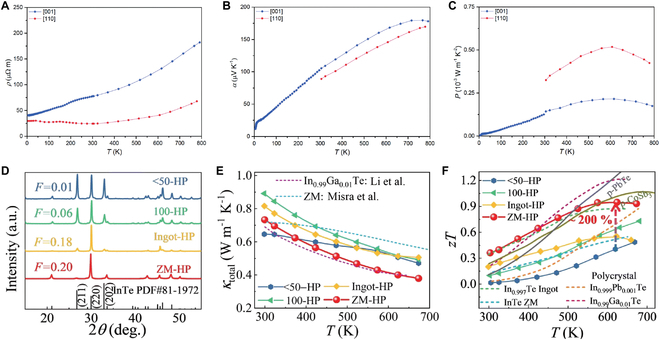
Temperature dependence of (A) *σ*, (B) *S*, and (C) PF along the *c* and [110] direction for single crystals of InTe. Reproduced with permission [108]. Copyright 2021 the Royal Society of Chemistry. (D) PXRD patterns of the <50-HP, 100-HP, Ingot-HP, and ZM-HP InTe samples. Comparison of the temperature-dependent (E) total thermal conductivity and (F) *zT* of these InTe samples as well as the previously reported InTe samples. Reproduced with permission [128]. Copyright 2023 the Royal Society of Chemistry.

Misra et al. [108] grew InTe single crystals using the Bridgman–Stockbarger method and demonstrated a noticeable anisotropy in electrical resistivity and Seebeck coefficient when comparing the *c* axis and [110] directions of the crystal structure (Fig. 15A to C). Furthermore, the lattice thermal conductivity along the [110] direction is exceptionally low, only 0.32 W m^−1^ K^−1^ at 780 K. A combination of the extremely low thermal conductivity and relatively high PF yields a high *zT* value of 0.61 at 780 K.

The TE properties of polycrystalline InTe can be improved through texture modulation [128]. The main step was through the oriented crystal hot-deformation method, which is a process of taking the top part of the InTe single crystal and applying a uniaxial pressure in the direction perpendicular to the (110) plane. The texture degrees were evaluated by analyzing the relative peak intensities from the powder x-ray diffraction (PXRD) patterns of the samples. The results indicated that the ZM-HP sample obtained from the single crystal treated with hot pressing (i.e., hot-deformation method) exhibited the highest texture of (110), as shown in Fig. 15D. It has been previously reported that the lattice thermal conductivity along the [110] direction is lower than that along the [001] direction due to the bonding asymmetry and lattice anharmonicity in InTe [32,46,102]. Consequently, the lattice thermal conductivity decreases with the increase of the [110] texture degree, as shown in Fig. 15E. Finally, the ZM-HP sample exhibited a peak *zT* value of 0.95 at 623 K, as shown in Fig. 15F.

**Fig. 16.**
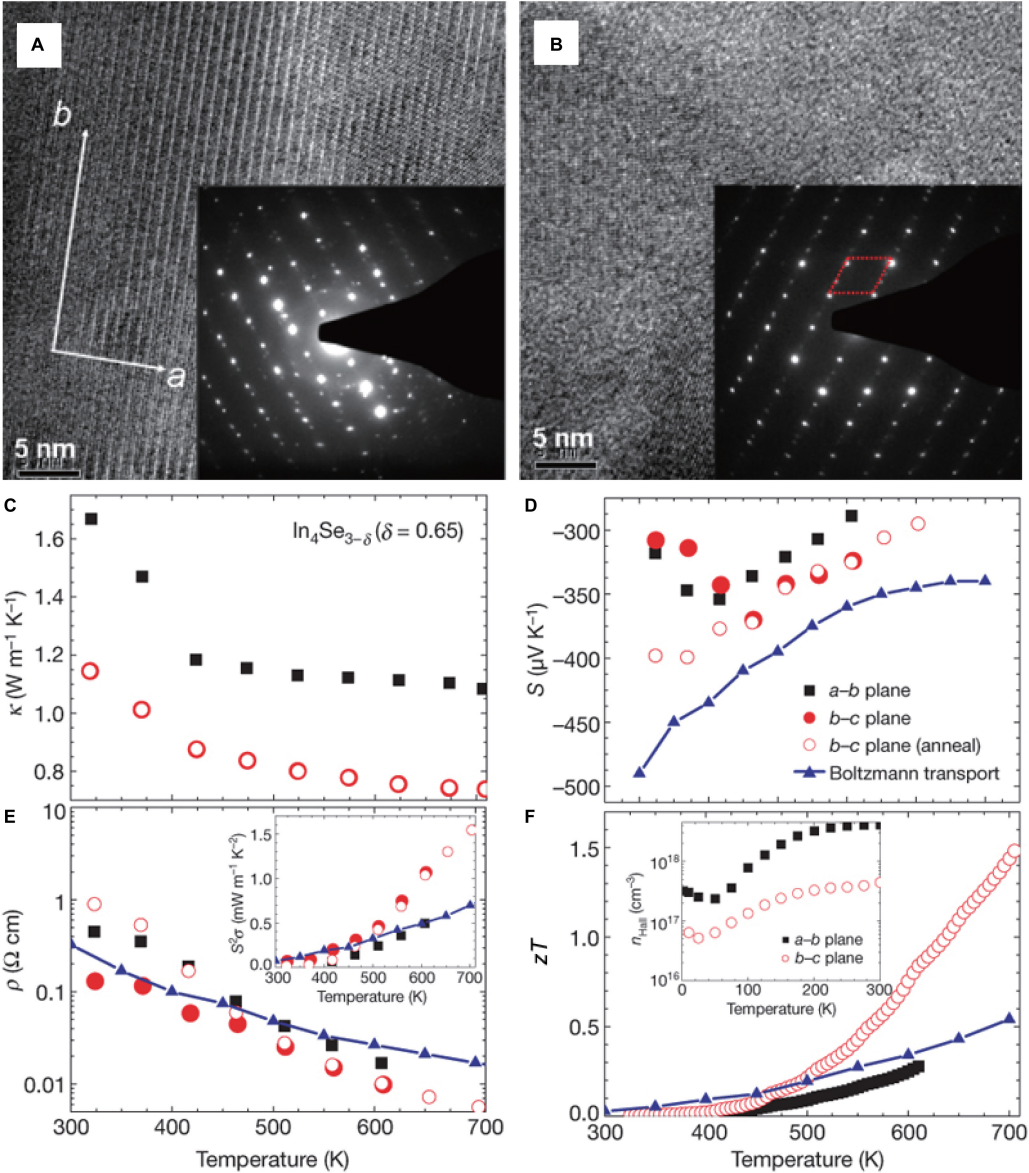
(A and B) High-resolution TEM images and corresponding electron diffraction patterns of In_4_Se_3−*δ*_ (*δ* = 0.22). (A) The *a*-*b* plane. The diffraction patterns exhibited quasi-one-dimensional chains aligned along the *b* axis. The presence of Bragg spots with superstructure peaks along the *b* axis is attributed to commensurate lattice distortions. (B) The cross-sectional plane of the crystal. The red rhomboid highlights the diffraction pattern corresponding to the unit cell. (C to F) Anisotropic TE properties as functions of temperature, and angle-averaged results from Boltzmann transport calculations for In_4_Se_3−*δ*_ (*δ* = 0.65) crystal. Open and filled symbols represent measurements taken before and after the heat treatment (450 °C, 24 h), respectively. Reproduced with permission [42]. Copyright 2009 Macmillan Publishers Limited.

Rhyee et al. [42] discovered that the CDW instability induced a significant anisotropy in the electrical and thermal transport properties of In_4_Se_3−*x*_ crystals (Fig. 16). Over the temperature range of 300 to 700 K, In_4_Se_2.35_ crystals exhibited considerably lower thermal conductivity along the *b*–*c* plane than along the *a*-*b* plane. The thermal conductivity along the *b*–*c* plane is ≤1.2 W m^−1^ K^−1^ at 300 K and 0.74 W m^−1^ K^−1^ at 705 K. This decrease in the thermal conductivity is caused by Peierls lattice distortion in the *b*–*c* plane due to the CDW effect. Furthermore, the Peierls distortion effect causes a decline in carrier concentration along the *b*–*c* plane. Ultimately, an exceptionally high *zT* value of 1.48 at 705 K was obtained along the *b*–*c* plane for In_4_Se_2.35_ crystals.

### Nanostructuring

**Fig. 17.**
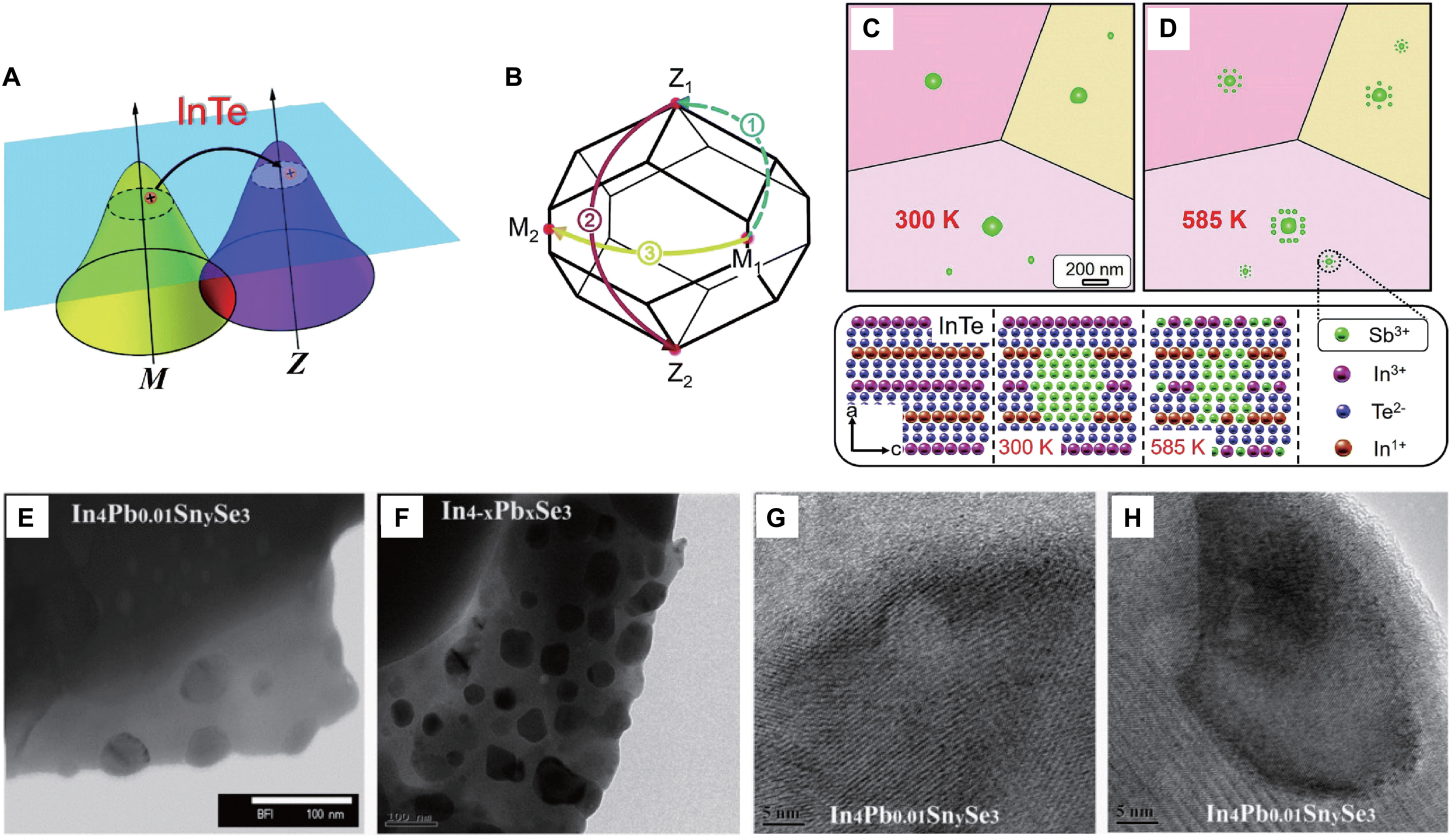
(A) Diagram illustrating nonequivalent intervalley scattering, identified as the primary scattering mechanism for the carriers at elevated temperatures and (B) different forms of intervalley scattering in InTe. Based on the TEM observations, (C) a portion of Sb appears as nanoprecipitates in the matrix at 300 K, and (D) gradually integrates into the lattice at temperatures exceeding 585 K. Reproduced with permission [129]. Copyright 2019 the Royal Society of Chemistry. Nanostructures embedded in In_4_Pb_0.01_Sn_0.03_Se_3_ and In_3.96_Pb_0.04_Se_3_ samples: (E and F) TEM images revealed spherical or ellipsoidal nanoparticles. (G and H) High-resolution TEM images revealed embedded In nanoprecipitates within the host matrix, exhibiting coherent or semi-coherent interfaces. Reproduced with permission [103]. Copyright 2013 Wiley-VCH Verlag GmbH & Co. KGaA, Weinheim.

As shown in Fig. 17A to D, Zhu et al. [129] introduced trace amounts of Sb nanoprecipitates into InTe, successfully converting the dominant scattering mechanism from intervalley scattering to acoustic phonon scattering (APS) at elevated temperatures. As a result, carrier mobility and PF show notable improvement, and ultimately, the InTe-Sb_0.01_ sample achieved a peak *zT* value of 0.8 at 623 K.

Nanostructuring has also been shown to be an effective method for enhancing the TE performance of the In_4_Se_3_ system. When In_4_Se_3−*x*_ samples were synthesized through mechanical alloying and hot-pressing techniques, nanoscale In precipitates were easily formed, which increased phonon scattering and resulted in a reduction in thermal conductivity. Specifically, the thermal conductivity of the In_4_Se_2.2_ sample reaches a minimum value of 0.41 W m^−1^ K^−1^ and a maximum *zT* value of 1.13 at 723 K [104]. In a separate study, Lin et al. [103] observed the In nanoparticles (i.e., 5- to 70-nm particles in the Pb/Sn co-doped In_4_Se_3_ sample and 3- to 100-nm particles in the Pb-doped sample) using TEM (Fig. 17E to H). These nanoparticles significantly enhance phonon scattering, resulting in a decrease in lattice thermal conductivity. The thermal conductivity can reach approximately 0.56 W m^−1^ K^−1^ at 733 K in the Pb/Sn co-doped sample, which is notably lower than those of undoped In_4_Se_3_ (0.65 W m^−1^ K^−1^) and Pb-doped In_4−*x*_Pb*_x_*Se_3_ (0.75 W m^−1^ K^−1^). Other approaches, such as the Cu intercalation and In nanoprecipitation produced by Cu embedding and Br substitution in In_4_Se_2.5_ polycrystals [130], Cu nanoincorporation in In_4_Se_3_ bulk materials through thermal decomposition of Cu(OAc)_2_ [117], and the formation of biphasic structure by combining In_4_Se_3_ and In nanostructures [131], have also been found to effectively promote the reduction of lattice thermal conductivity in In_4_Se_3_.

### Grain size engineering

**Fig. 18.**
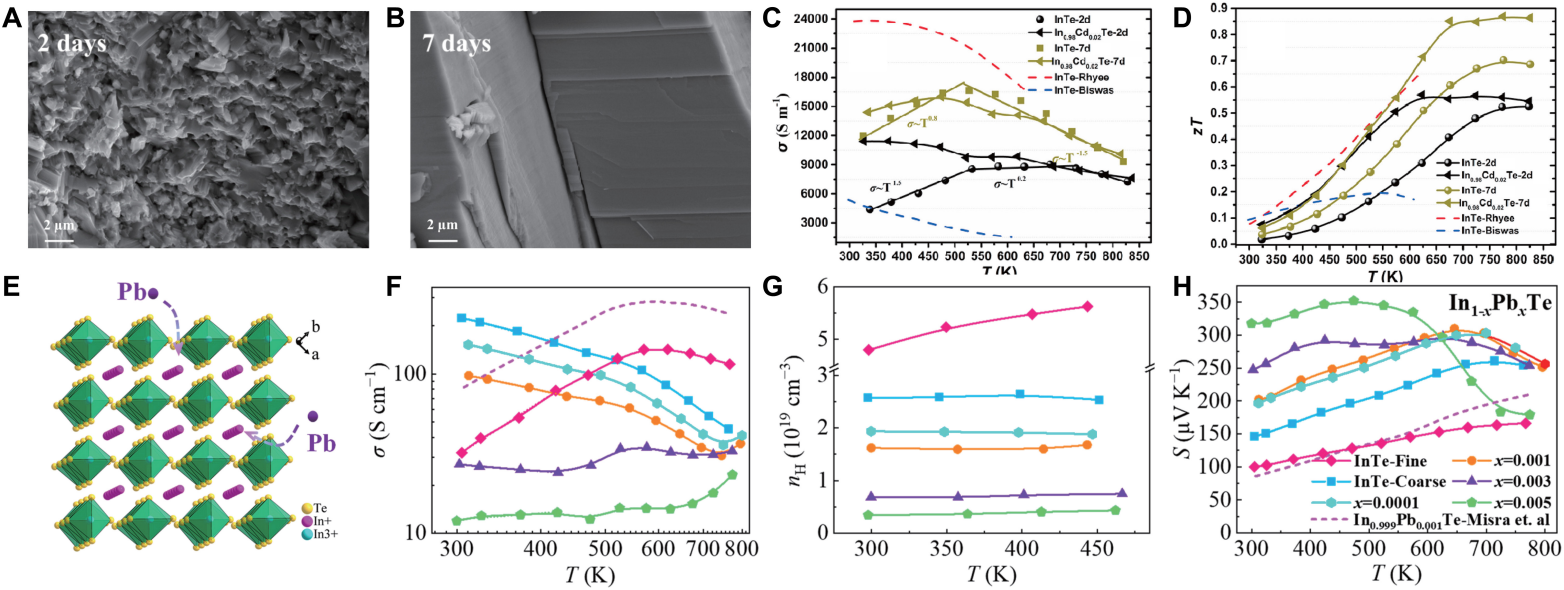
Field emission scanning electron microscopy (FESEM) images of InTe with different annealing times of (A) 2 d and (B) 7 d. Temperature-dependent (C) *σ* and (D) *zT* compared to the previously reported values [32,109]. Reproduced with permission [92]. Copyright 2020 Elsevier B.V. (E) Schematic of Pb substitution at the In^+^ site in InTe. Temperature-dependence of (F) *σ*, (G) *n*_H_, and (H) *S*. [102] Reproduced with permission [105]. Copyright 2023 by Zhou et al.

The grain size of undoped and Cd-doped InTe samples was notably enlarged by extending the annealing time from 2 to 7 d (Fig. 18A and B) [92]. The InTe-7d sample exhibits higher carrier mobility at room temperature (11 cm^−2^ v^−1^ s^−1^), ~110% enhancement compared to the InTe-2d sample. This increase in carrier mobility was attributed to the larger grain size and reduced scattering effect from the ionizing impurities [92], resulting in a substantial improvement in electrical conductivity (Fig. 18C) and PF. Specifically, after annealing for 7 d, the peak *zT* values at 773 K of the undoped InTe sample and the In_0.98_Cd_0.02_Te sample are 0.70 and 0.87, respectively, obviously higher than those of the samples annealed for 2 d (*zT* ~ 0.5 at 773 K) (Fig. 18D). Li et al. [30] also demonstrated that grain coarsening in the InTe material can achieve a transition from dominant GBS to APS, thus improving the Hall mobility and PF. As a consequence, the coarse-grain InTe sample exhibited a peak *zT* of 0.85 at 650 K, outperforming the fine-grain InTe sample (*zT* = ~0.6 at 650 K). Similarly, Feng et al. [128] also found that grain growth not only suppressed GBS, thereby enhancing electrical conductivity, but also exerted negligible influence on the density-of-states effective mass (m*_DOS_). This implies that enhancing electrical conductivity through grain growth has little impact on *S*, which is advantageous for optimizing electrical performance. Furthermore, attenuating GBS through grain enlargement significantly enhances the TE properties of InTe materials, particularly at low temperatures. Moreover, Zhou et al. [105] concluded that both grain size and carrier concentration notably influence the GBS behavior in InTe sample. They tuned the carrier concentration by substituting Pb for In^+^ and found that GBS is predominantly governed by grain size when the carrier concentration exceeds 0.7 × 10^19^ cm^−3^, whereas at lower carrier concentrations, it is primarily influenced by the carrier concentration itself. Therefore, by modulating both grain size and carrier concentration, the TE performance of InTe materials can be optimized across a broad temperature range, leading to enhanced energy conversion efficiency in practical applications.

## Summary and Outlook

### Indium tellurides

**Fig. 19.**
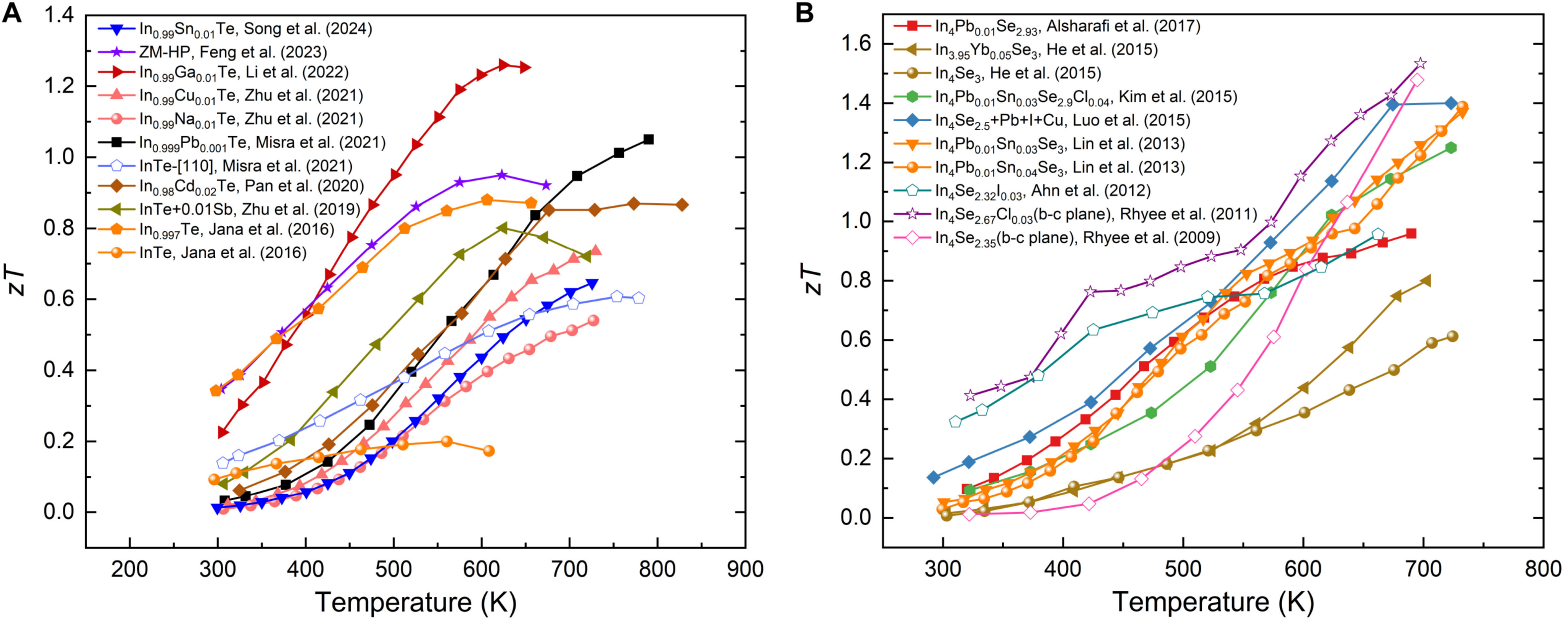
(A) Temperature-dependent *zT* values of the reported InTe-based samples [30,32,45,92,102,108,112,128,129]. (B) Temperature-dependent *zT* values of the reported In_4_Se_3_-based single crystals [29,42,158] and polycrystals [103,107,111,113,159].

The emergence of InTe in the TE field has been attributed to its relatively high electrical conductivity and very low thermal conductivity [108]. The low thermal conductivity is mainly due to the strong anharmonicity resulting from weakly bound In^1+^ ions exhibiting dynamic lone pair expression [46]. Experimental studies have shown that doping InTe with elements such as Pb, Ga, Cd, and Sb significantly improves the TE performance. Figure 19A shows the temperature dependence of the *zT* values for representative InTe-based samples reported in literature. Manipulating the grain size of InTe for the purification of GBS has also been demonstrated to enhance the TE performance. All experimental studies on InTe thus far have exclusively reported p-type behavior, which is primarily attributed to the prevailing In^1+^ vacancy defect that stabilizes the Fermi level near the valence band edge [32,45,46,108]. It is predicted that if InTe can be n-doped, an enhanced PF and *zT* could be achieved in n-type InTe, owing to its high valley degeneracy of the CBM [45]. For future research, exploring new dopants, grain size engineering, or realizing the n-type transition may be considered to manipulate the TE performance of InTe.

There are limited reports on other In–Te systems (i.e., In_2_Te_3_, In_4_Te_3_, In_3_Te_4_, and In_2_Te_5_) in the TE field. The electrical properties of In_2_Te_3_ are generally poor, making it challenging to enhance its TE performance [132,133]. Leveraging its inherent structural vacancy, In_2_Te_3_ has been utilized as an alloying component in various material systems, including Cu_2_SnSe_4_ [134], CuGaTe [135], SnTe [136], GeTe [137], and InSb [138], to form tunable solid solutions. The vacancy-mediated solid solution not only facilitates carrier concentration optimization but also introduces additional scattering centers. This strategic incorporation enables band structure engineering while simultaneously suppressing lattice thermal conductivity via enhanced phonon scattering mechanisms. Similarly, In_2_Te_3_ nanowires, as a relatively underexplored one-dimensional nanomaterial, offer a potential for future scientific exploration [139]. Additionally, while the thermal conductivity of In_4_Te_3_ [89] and In_2_Te_5_ [140] can be reduced to very low levels, their high electrical resistivity limits the increase of the *zT* value. Thus, reducing the electrical resistivity could be the starting point for research with the aim of significantly enhancing the TE performance.

### Indium selenides

In_4_Se_3_ exhibits excellent TE performance in both single crystals and polycrystals. The reported *zT* values as a function of temperature for the representative samples are plotted in Fig. 19B. In_4_Se_3_ shows anisotropy as a result of its distinctive crystal structure. The Peierls distortion of the crystal lattice induced by the CDW along the *b*–*c* plane results in strong electron–phonon coupling. Accordingly, the In_4_Se_2.35_ single crystal demonstrates exceptionally low thermal conductivity along the *b*–*c* plane, achieving a high *zT* value of 1.48 at 705 K [42]. In addition to monocrystalline In_4_Se_2.35_, polycrystalline In_4_Pb_0.01_Sn*_y_*Se_3_ (*y* = 0.03, 0.04) has demonstrated a high *zT* value of 1.4 at 733 K [103], mainly because of the successful doping of Pb/Sn as an electron donor in In_4_Se_3_. The Pb/Sn doping increases the Hall carrier concentration and decreases the thermal conductivity. Previous experimental researches on In_4_Se_3_ have demonstrated that its TE properties can be greatly enhanced via Se deficiency manipulation, doping engineering, nanostructuring, or employing a combination of these strategies. Se deficiency has been extensively investigated as a prominent approach. Nanostructuring, such as generating nanoscale precipitates, has been designed to scatter phonons effectively, resulting in reduced thermal conductivity. Future research could focus on the exploration of Pb-free single-element doping or multi-element co-doping, as well as nanostructuring, with the aim of discovering innovative methods for boosting the TE performance of In_4_Se_3_.

There has been some research on InSe in recent years, but its TE performance has not been significantly improved. Currently, the highest *zT* value of 0.66 was reported in In_1.25_Sn_0.05_Se at 700 K [106]. InSe possesses a relatively wide energy gap of ~1.2 eV, leading to a low intrinsic carrier concentration of around 10^14^ cm^−3^ [118]. Consequently, the lower carrier concentration limits the electrical conductivity and Seebeck coefficient. In contrast with In_4_Se_3_, In_2_Se_3_ has been less studied, but it has shown promise with a maximum *zT* of 1.23 [124]. This opens up more possibilities for studying this material system. Additionally, In_6_Se_7_ has successfully undergone a transition from p-type to n-type conduction, offering further opportunities for regulating its TE properties. Overall, for these materials, methods such as doping, nanostructuring, or achieving p–n transition could be applied to further enhance their TE properties. In addition to their TE performance, the unique plastic deformation capability of InSe warrants comprehensive investigation. Recent studies [101,141] have shown that the interlayer slip mechanism and unusually high ductility offer a robust foundation for the mechanical performance of flexible electronic devices.

### Indium sulfides

Up to now, there has been limited research on TE properties of In_2_S_3_. The lattice thermal conductivity of In_2_S_3_ can be effectively reduced by substituting Mg for In. The optimized composition In_1.95_Mg_0.05_S_3_ achieves a peak *zT* of 0.53 at 700 K, which is primarily attributed to its exceptional PF at this temperature [127]. Additionally, theoretical calculations have shown that the lattice thermal conductivity of 2-dimensional InS is relatively low at room temperature, ~0.6 W m^−1^ K^−1^ [142]. Since both InS and In_2_S_3_ show low thermal conductivity, one can start with optimizing the electrical transport properties through various possible methods (e.g., doping) so as to enhance their TE performance. The exploration of In_6_S_7_ and In_3_S_4_ for TE applications remains largely underexplored, highlighting the need for a comprehensive research strategy that combines cutting-edge experimental techniques with first-principles theoretical investigations.

Despite the substantial progress in optimizing the TE properties of the binary indium-based chalcogenides described above, their practical application still faces several key challenges: (a) regarding material stability, some indium-based chalcogenides exhibit pronounced temperature-dependent phase transitions (e.g., In_2_Te_3_ [10,61], In_2_Se_3_ [64–66], and In_2_S_3_ [74]), which may restrict their operating temperature range in TE applications; (b) regarding scalability, it remains challenging to balance batch preparation and microstructure control using current synthesis methods (e.g., melting combined with spark plasma sintering [30,111] and NEFTs [124]); (c) in terms of cost-effectiveness, the scarcity of high-purity indium and the complexity of the doping process substantially increase manufacturing costs. To address these challenges, future research could focus on several key directions: developing synergistic phase transition modulation strategies [143–146], such as suppressing harmful transitions to expand the operational temperature range of materials; exploring new scalable synthesis routes, such as batch production processes used in Mg–Si–Sn [147], Mg–Si [148], and Bi–Sb–Te [149,150] systems; and reducing material costs by developing recycling techniques for waste materials and investigating low-cost element substitutes, such as Cu and Ag. In addition, it is recommended to integrate advanced computational and experimental approaches within these systems [151–153]. These include first-principles calculations, high-throughput screening, automated synthesis, and characterization techniques. Such integration would enable the systematic optimization of material compositions and processing parameters, thereby accelerating the pace of materials research and development.

Overall, this Review elaborates on the crystal structure, electronic structure, and phonon dispersion for binary indium-based chalcogenides, subsequently summarizing TE optimization strategies, i.e., defect engineering, crystal orientation engineering, nanostructuring, and grain size engineering. The perspectives on the challenges and opportunities in the TE field for binary indium-based chalcogenides are also discussed. Crucially, the comprehensive computational study presented in this Review, integrating electronic structure calculations, orbital and bonding analysis, and phonon dispersion evaluations, provides a robust framework for comprehending the intricate structure–property relationships inherent in these materials and establishes a guide for innovative strategies aimed at boosting the TE performance.

## Data Availability

The data are available from the corresponding author upon reasonable request.

## References

[1] Li D, Gong Y, Chen Y, Lin J, Khan Q, Zhang Y, Li Y, Zhang H, Xie H. Recent progress of two-dimensional thermoelectric materials. Nano-Micro Lett. 2020;12:Article 36. 10.1007/s40820-020-0374-x PMC7770719 34138247

[2] Chen Z-G, Han G, Yang L, Cheng L, Zou J. Nanostructured thermoelectric materials: Current research and future challenge. Prog Nat Sci Mater Int. 2012;22(6):535–549.

[3] Wei T-R, Qin Y, Deng T, Song Q, Jiang B, Liu R, Qiu P, Shi X, Chen L. Copper chalcogenide thermoelectric materials. Sci China Mater. 2018;62(1):8–24.

[4] He J, Tritt TM. Advances in thermoelectric materials research: Looking back and moving forward. Science. 2017;357(6358):Article eaak9997. 28963228 10.1126/science.aak9997

[5] Li A, Fu C, Zhao X, Zhu T. High-performance Mg_3_Sb_2-*x*_Bi*_x_* thermoelectrics: Progress and perspective. Research. 2020;2020:Article 1934848. 33623901 10.34133/2020/1934848 PMC7877388

[6] Snyder GJ, Toberer ES. Complex thermoelectric materials. Nat Mater. 2008;7(2):105–114. 18219332 10.1038/nmat2090

[7] Gong Y, Ying P, Zhang Q, Liu Y, Huang X, Dou W, Zhang Y, Li D, Zhang D, Feng T, et al. Realizing the high thermoelectric performance of highly preferentially oriented SnSe based nanorods via band alignment. Energy Environ Sci. 2024;17(4):1612–1623.

[8] Yang J, Caillat T. Thermoelectric materials for space and automotive power generation. MRS Bull. 2006;31(3):224–229.

[9] Fukutani K, Shakouri A. Design of bulk thermoelectric modules for integrated circuit thermal management. IEEE Trans Compon Packag Manuf Technol. 2006;29(4):750–757.

[10] Vora-ud A, Thanachayanont C, Jugsujinda S, Amornkitbamrung V, Seetawana T. Study on electronic structure of *β*-In_2_Te_3_ thermoelectric material for alternative energy. Procedia Eng. 2011;8:2–7.

[11] Wang Y, Tang Z, Tan S, Kotov NA. Biological assembly of nanocircuit prototypes from protein-modified CdTe nanowires. Nano Lett. 2005;5(2):243–248. 15794604 10.1021/nl0482682

[12] Alsalama MM, Hamoudi H, Abdala A, Ghouri ZK, Youssef KM. Enhancement of thermoelectric properties of layered chalcogenide materials. Rev Adv Mater Sci. 2020;59(1):371–378.

[13] Szczech JR, Higgins JM, Jin S. Enhancement of the thermoelectric properties in nanoscale and nanostructured materials. J Mater Chem. 2011;21(12):4037–4055.

[14] Zhang J, Song L, Iversen BB. Insights into the design of thermoelectric Mg_3_Sb_2_ and its analogs by combining theory and experiment. npj Comput Mater. 2019;5(1):Article 76.

[15] Xu R, Chen Z, Li Q, Yang X, Wan H, Kong M, Bai W, Zhu N, Wang R, Song J, et al. Realizing plain optimization of the thermoelectric properties in BiCuSeO oxide via self-substitution-induced lattice dislocations. Research. 2023;6:Article 0123. 37287891 10.34133/research.0123 PMC10243199

[16] Jia B, Wu D, Xie L, Wang W, Yu T, Li S, Wang Y, Xu Y, Jiang B, Chen Z, et al. Pseudo-nanostructure and trapped-hole release induce high thermoelectric performance in PbTe. Science. 2024;384(6691):81–86. 38574137 10.1126/science.adj8175

[17] Wang Y, Liu W-D, Shi X-L, Hong M, Wang L-J, Li M, Wang H, Zou J, Chen Z-G. Enhanced thermoelectric properties of nanostructured *n*-type Bi_2_Te_3_ by suppressing Te vacancy through non-equilibrium fast reaction. Chem Eng J. 2020;391: Article 123513.

[18] Wu G, Zhang Q, Tan X, Fu Y, Guo Z, Zhang Z, Sun Q, Liu Y, Shi H, Li J, et al. Bi_2_Te_3_-based thermoelectric modules for efficient and reliable low-grade heat recovery. Adv Mater. 2024;36(26):Article 2400285. 10.1002/adma.202400285 38613131

[19] Lu Y, Zhou Y, Wang W, Hu M, Huang X, Mao D, Huang S, Xie L, Lin P, Jiang B, et al. Staggered-layer-boosted flexible Bi_2_Te_3_ films with high thermoelectric performance. Nat Nanotechnol. 2023;18(11):1281–1288. 37500776 10.1038/s41565-023-01457-5

[20] Ma B, Li Y, Zhu L, Zhang F, Li X, Shi Y, Liang P, Peng Z, Chao X, Yang Z, et al. Modulation doping promoted ultrahigh electron mobility and enhanced thermoelectric performance in PbTe. Chem Eng J. 2024;488: Article 150647.

[21] Gao F, Cai J, Li M, Chen Z, Wang Y, Zhang Z, Chen L, Hu D, Tan X, Wu J, et al. Thermoelectric performance optimization of n-type PbTe by In and Cu_2_Te co-doping and anomalous temperature-dependent transport. J Mater Chem A. 2024;12(20):11875–11882.

[22] Liu S, Yu Y, Wu D, Xu X, Chao X, Yang Z, He J. Strained endotaxial PbS nanoprecipitates boosting ultrahigh thermoelectric quality factor in n-type PbTe as-cast ingots. Small. 2021;17(50):Article 2104496. 10.1002/smll.202104496 34658144

[23] Huang S, Yang H, Li Y, Guo Z, Zhang Q, Cai J, Wu J, Tan X, Liu G, Song K, et al. Optimizing GeTe-based thermoelectric generator for low-grade heat recovery. Appl Energy. 2023;349: Article 121584.

[24] Li M, Xu S-D, Hong M, Lyu W-Y, Wang Y, Dargusch M, Zou J, Cheng H-M, Chen Z-G. Roles of anion sites in high-performance GeTe thermoelectrics. Adv Funct Mater. 2022;32(48):Article 2208579.

[25] Bu Z, Zhang X, Hu Y, Chen Z, Lin S, Li W, Pei Y. An over 10% module efficiency obtained using non-Bi_2_Te_3_ thermoelectric materials for recovering heat of <600 K. Energy Environ Sci. 2021;14(12):6506–6513.

[26] Savelli G, Colonna J-P, Coudrain P, Faucherand P, Royer A, Collin L-M, Amnache A, Frechette L. High power 2.5D integrated thermoelectric generators combined with microchannels technology. Energy. 2022;252: Article 123984.

[27] Chen H, Zhang Z, Liang J, Miao L, Zhou Q, Peng Y, Liu C, Chen J, Lai H. Phonon relaxation effect by regeneration of nano-inclusions in SiGe for ultralow thermal conductivity and enhanced thermoelectric performance. Mater Today Phys. 2024;43: Article 101405.

[28] Sojo-Gordillo JM, Sierra CD, Diez GG, Segura-Ruiz J, Bonino V, Eroles MN, Gonzalez-Rosillo JC, Estrada-Wiese D, Salleras M, Fonseca L, et al. Superior thermoelectric performance of SiGe nanowires epitaxially integrated into thermal micro-harvesters. Small. 2023;19(17): Article 2206399. 10.1002/smll.202206399 36720043

[29] Rhyee J-S, Ahn K, Lee KH, Ji HS, Shim JH. Enhancement of the thermoelectric figure-of-merit in a wide temperature range in In_4_Se_3-*x*_Cl_0.03_ bulk crystals. Adv Mater. 2011;23(19):2191–2194. 21469219 10.1002/adma.201004739

[30] Li F, Liu X, Ma N, Chen L, Wu LM. Thermoelectric zintl compound In_1-*x*_Ga*_x_*Te: Pure acoustic phonon scattering and dopant-induced deformation potential reduction and lattice shrink. Angew Chem Int Ed Engl. 2022;61(35): Article e202208216. 35817753 10.1002/anie.202208216

[31] Nishino T, Hamakawa Y. Preparation and properties of InS single crystals. Jpn J Appl Phys. 1977;16(8):1291–1300.

[32] Jana MK, Pal K, Waghmare UV, Biswas K. The origin of ultralow thermal conductivity in InTe: Lone-pair-induced anharmonic rattling. Angew Chem Int Ed Engl. 2016;55(27):7792–7796. 26918541 10.1002/anie.201511737

[33] Sucharitakul S, Goble NJ, Kumar UR, Sankar R, Bogorad ZA, Chou F-C, Chen Y-T, Gao XPA. Intrinsic electron mobility exceeding 10^3^ cm^2^/(V s) in multilayer InSe FETs. Nano Lett. 2015;15(6):3815–3819. 25924062 10.1021/acs.nanolett.5b00493

[34] Yang W, Xu N, Zhang H. Nonlinear absorption properties of indium selenide and its application for demonstrating pulsed Er-doped fiber laser. Laser Phys Lett. 2018;15(10): Article 105101.

[35] Ho C-H, Chen Y-H, Ho J-H. Optical and photodetector properties of stripe-like InS crystal. RSC Adv. 2016;6(99):97445–97448.

[36] Hogg JHC, Sutherland HH, Williams DJ. Crystallographic evidence for the existence of the phases In_4_Se_3_ and In_4_Te_3_ which contain the homonuclear triatomic cation (In_3_)^5+^. J Chem Soc D. 1971;(23):1568–1569.

[37] Chen Y-C, Liu P-F, Chen L, Wu L-M. Thermoelectric properties of Ag-doped In_4_Se_2.95_ polycrystalline compound. Chin J Struct Chem. 2016;35(12):1868–1875.

[38] Yin X, Liu J-Y, Chen L, Wu L-M. High thermoelectric performance of In_4_Se_3_-based materials and the influencing factors. Acc Chem Res. 2018;51(2):240–247. 29313668 10.1021/acs.accounts.7b00480

[39] Sznajder M, Rushchanskii KZ, Kharkhalis LY, Bercha DM. Similarities of the band structure of In_4_Se_3_ and InSe under pressure and peculiarities of the creation of the band gap. Phys Status Solidi. 2006;243(3):592–609.

[40] Lim YS, Jeong M, Seo W-S, Lee J-H, Park C-H, Sznajder M, Kharkhalis LY, Bercha DM, Yang J. Condenson state and its effects on thermoelectric properties in In_4_Se_3_. J Phys D Appl Phys. 2013;46(27): Article 275304.

[41] Han G, Chen Z-G, Drennan J, Zou J. Indium selenides: Structural characteristics, synthesis and their thermoelectric performances. Small. 2014;10(14):2747–2765. 24729463 10.1002/smll.201400104

[42] Rhyee J-S, Lee KH, Lee SM, Cho E, Kim SI, Lee E, Kwon YS, Shim JH, Kotliar G. Peierls distortion as a route to high thermoelectric performance in In_4_Se_3-*δ*_ crystals. Nature. 2009;459(7249):965–968. 19536260 10.1038/nature08088

[43] Das A, Banerji P. Antibonding or nonbonding interaction-driven phonon modes softening and wave-like interband thermal conduction in layered In_4_Te_3_ under the framework of wigner transport formalism. ACS Appl Energy Mater. 2023;6(22):11521–11531.

[44] Hogg JHC, Sutherland HH. Indium telluride. Acta Cryst. 1976;32(9):2689–2690.

[45] Song L, Zhang J, Mamakhel A, Iversen BB. Crystal structure, electronic transport, and improved thermoelectric properties of doped InTe. ACS Appl Electron Mater. 2023;6(5):2925–2934.

[46] Zhang J, Roth N, Tolborg K, Takahashi S, Song L, Bondesgaard M, Nishibori E, Iversen BB. Direct observation of one-dimensional disordered diffusion channel in a chain-like thermoelectric with ultralow thermal conductivity. Nat Commun. 2021;12(1):Article 6709. 34795243 10.1038/s41467-021-27007-y PMC8602660

[47] Popović S, Tonejc A, Čelustka B, Gržeta-Plenković B, Trojko R. Revised and new crystal data for indium selenides. J Appl Crystallogr. 1979;12(4):416–420.

[48] Čelustka B, Popović S. The synthesis of In_5_Se_6_ and In_2_Se from InSe by zone-melting process. J Phys Chem Solids. 1974;35(2):287–289.

[49] Hollingsworth JA, Poojary DM, Clearfield A, Buhro WE. Catalyzed growth of a metastable InS crystal structure as colloidal crystals. J Am Chem Soc. 2000;122(14):3562–3563.

[50] Kushwaha P, Patra A, Anjali E, Surdi H, Singh A, Gurada C, Ramakrishnan S, Prabhu S, Gopal AV, Thamizhavel A. Physical, optical and nonlinear properties of InS single crystal. Opt Mater. 2014;36(3):616–620.

[51] Walther R, Deiseroth HJ. Redetermination of the crystal structure of indium monosulfide, InS. Z Kristallogr Cryst Mater. 1995;210(5):360.

[52] Cheng M, Chen S, Du Z, Liu X, Cui J. Improvement in thermoelectric performance of In_6_Se_7_ by substitution of Sn for In. Phys Status Solidi A. 2016;213(8):2176–2182.

[53] Cui J, Cheng M, Wu W, Du Z, Chao Y. Engineering band structure via the site preference of Pb^2+^ in the In^+^ site for enhanced thermoelectric performance of In_6_Se_7_. ACS Appl Mater Interfaces. 2016;8(35):23175–23180. 27541319 10.1021/acsami.6b07238

[54] Abdallah HB, Bennaceur R. Electronic structure of the hexaindium heptasulfide In_6_S_7_. Phys B. 2006;382(1-2):181–188.

[55] Deiseroth HJ, Pfeifer SA. Strukturchemie und valenz von In_6_S_7_ neubestimmung der kristallstruktur. Z Kristallogr Cryst Mater. 1993;207:45–52.

[56] Han G, Chen Z-G, Sun C, Yang L, Cheng L, Li Z, Lu W, Gibbs ZM, Snyder GJ, Jack K, et al. A new crystal: Layer-structured rhombohedral In_3_Se_4_. CrystEngComm. 2014;16(3):393–398.

[57] Han G, Chen Z-G, Yang L, Cheng L, Drennan J, Zou J. Phase control and formation mechanism of new-phase layer-structured rhombohedral In_3_Se_4_ hierarchical nanostructures. Cryst Growth Des. 2013;13(11):5092–5099.

[58] Geller S, Jayaraman A, Hull GW. Crystal chemistry and superconductivity of pressure-induced phases in the In-Te system. J Phys Chem Solids. 1965;26(2):353–361.

[59] Karakostas BT, Flevaris NF, Vlachavas N, Bleris GL, Economou NA. The ordered state of In_3_Te_4_. Acta Cryst. 1978;34(1):123–126.

[60] Zavrazhnov AY, Naumov AV, Anorov PV, Goncharov EG, Sidei VI, Pervov VS. T-*x* phase diagram of the In-S system. Inorg Mater. 2006;42(12):1294–1298.

[61] Huangfu Y, Qin B, Lu P, Zhang Q, Li W, Liang J, Liang Z, Liu J, Liu M, Lin X, et al. Low temperature synthesis of 2D p-type *α*-In_2_Te_3_ with fast and broadband photodetection. Small. 2024;20(28):Article 2309620. 10.1002/smll.202309620 38294996

[62] Desai RR, Lakshminarayana D, Patel PB, Patel PK, Panchal CJ. Growth and structural properties of indium sesquitelluride (In_2_Te_3_) thin films. Mater Chem Phys. 2005;94(2-3):308–314.

[63] Sowjanya V, Bangera KV, Shivakumar GK. Effect of substrate temperature and film thickness on the thermoelectric properties of In_2_Te_3_ thin films. J Alloys Compd. 2017;715:224–229.

[64] Landuyt JV, Tendeloo GV, Amelinckx S. Phase transitions in In_2_Se_3_ as studied by electron microscopy and electron diffraction. Phys Status Solidi A. 1975;30(1):299–314.

[65] Jasinski J, Swider W, Washburn J, Liliental-Weber Z, Chaiken A, Nauka K, Gibson GA, Yang CC. Crystal structure of *κ*-In_2_Se_3_. Appl Phys Lett. 2002;81(23):4356–4358.

[66] Li J, Li H, Niu X, Wang Z. Low-dimensional In_2_Se_3_ compounds: From material preparations to device applications. ACS Nano. 2021;15(12):18683–18707. 34870407 10.1021/acsnano.1c03836

[67] Popović S, Čelustka B, Bidjin D. X-ray diffraction measurement of lattice parameters of In_2_Se_3_. Phys Status Solidi A. 1971;6(1):301–304.

[68] Küepers M, Konze PM, Meledin A, Mayer J, Englert U, Wuttig M, Dronskowski R. Controlled crystal growth of indium selenide, In_2_Se_3_, and the crystal structures of *α*-In_2_Se_3_. Inorg Chem. 2018;57(18):11775–11781. 30153016 10.1021/acs.inorgchem.8b01950

[69] Li W, Sabino FP, de Lima FC, Wang T, Miwa RH, Janotti A. Large disparity between optical and fundamental band gaps in layered In_2_Se_3_. Phys Rev B. 2018;98(16): Article 165134.

[70] Osamura K, Murakami Y, Tomiie Y. Crystal structures of *α*-and *β*-indium selenide, In_2_Se_3_. J Phys Soc Jpn. 1966;21:Article 1848.

[71] Ye J, Soeda S, Nakamura Y, Nittono O. Crystal structures and phase transformation in In_2_Se_3_ compound semiconductor. Jpn J Appl Phys. 1998;37(8):4264–4271.

[72] Debbichi L, Eriksson O, Lebègue S. Two-dimensional indium selenides compounds: An ab initio study. J Phys Chem Lett. 2015;6(15):3098–3103. 26267208 10.1021/acs.jpclett.5b01356

[73] Zhou Y, Wu D, Zhu Y, Cho Y, He Q, Yang X, Herrera K, Chu Z, Han Y, Downer MC, et al. Out-of-plane piezoelectricity and ferroelectricity in layered *α*-In_2_Se_3_ nanoflakes. Nano Lett. 2017;17(9):5508–5513. 28841328 10.1021/acs.nanolett.7b02198

[74] Pistor P, Merino Álvarez JM, León M, Michiel M, Schorr S, Klenk R, Lehmann S. Structure reinvestigation of *α*-, *β*- and *γ*-In_2_S_3_. Acta Crystallogr Sect B Struct Sci Cryst Eng Mater. 2016;72(Pt 3):410–415. 10.1107/S2052520616007058 PMC4886618 27240773

[75] Sutherland HH, Hogg JHC, Walton PD. Indium polytelluride In_2_Te_5_. Acta Crystallogr Sect B Struct Crystallogr Cryst Chem. 1976;32:2539–2541.

[76] Zhang W, Sato N, Tobita K, Kimura K, Mori T. Unusual lattice dynamics and anisotropic thermal conductivity in In_2_Te_5_ due to a layered structure and planar-coordinated Te-chains. Chem Mater. 2020;32(12):5335–5342.

[77] Sanchela AV, Thakur AD, Tomy CV. Direction-dependent thermoelectric properties of a layered compound In_2_Te_5_ single crystal. J Electron Mater. 2022;51(5):2266–2272.

[78] Kresse G, Fürthmhller J. Efficient iterative schemes for ab initio total-energy calculations using a plane-wave basis set. Phys Rev B. 1996;54(16):11169–11186. 10.1103/physrevb.54.11169 9984901

[79] Blöchl PE. Projector augmented-wave method. Phys Rev B. 1994;50(24):17953–17979. 10.1103/physrevb.50.17953 9976227

[80] Perdew JP, Ruzsinszky A, Csonka GI, Vydrov OA, Scuseria GE, Constantin LA, Zhou X, Burke K. Restoring the density-gradient expansion for exchange in solids and surfaces. Phys Rev Lett. 2008;100(13): Article 136406. 18517979 10.1103/PhysRevLett.100.136406

[81] Grimme S, Ehrlich S, Goerigk L. Effect of the damping function in dispersion corrected density functional theory. J Comput Chem. 2011;32(7):1456–1465. 21370243 10.1002/jcc.21759

[82] Becke AD, Johnson ER. A simple effective potential for exchange. J Chem Phys. 2006;124(22): Article 221101. 16784253 10.1063/1.2213970

[83] Tran F, Blaha P. Accurate band gaps of semiconductors and insulators with a semilocal exchange-correlation potential. Phys Rev Lett. 2009;102(22): Article 226401. 19658882 10.1103/PhysRevLett.102.226401

[84] Dronskowski R, Blöchl PE. Crystal orbital Hamilton populations (COHP). Energy-resolved visualization of chemical bonding in solids based on density-functional calculations. J Phys Chem. 1993;97(33):8617–8624.

[85] Maintz S, Deringer VL, Tchougréeff AL, Dronskowski R. Lobster: A tool to extract chemical bonding from plane-wave based DFT. J Comput Chem. 2016;37(11):1030–1035. 26914535 10.1002/jcc.24300 PMC5067632

[86] Togo A, Tanaka I. First principles phonon calculations in materials science. Scr Mater. 2015;108:1–5.

[87] http://henriquemiranda.github.io/phononwebsite/phonon.html.

[88] Krukau AV, Vydrov OA, Izmaylov AF, Scuseria GE. Influence of the exchange screening parameter on the performance of screened hybrid functionals. J Chem Phys. 2006;125(22): Article 224106. 17176133 10.1063/1.2404663

[89] Shi X, Cho JY, Salvador JR, Yang J, Wang H. Thermoelectric properties of polycrystalline In_4_Se_3_ and In_4_Te_3_. Appl Phys Lett. 2010;96(16): Article 162108.

[90] Xu W, Liu Q, Zhou X, Lin J, Lin S, Lu M, Lin J. Effects of stresses on the thermoelectric properties of In_4_Se_3_. J Mater Chem C. 2024;12(14):5062–5072.

[91] Wu Y, Nan P, Chen Z, Zeng Z, Lin S, Zhang X, Dong H, Chen Z, Gu H, Li W, et al. Manipulation of band degeneracy and lattice strain for extraordinary PbTe thermoelectrics. Research. 2020;2020:Article 8151059. 32025663 10.34133/2020/8151059 PMC7000992

[92] Pan S, Liu H, Li Z, You L, Dai S, Yang J, Guo K, Luo J. Enhancement of the thermoelectric performance of InTe via introducing Cd dopant and regulating the annealing time. J Alloys Compd. 2020;813: Article 152210.

[93] Furness JW, Kaplan AD, Ning J, Perdew JP, Sun J. Accurate and numerically efficient r^2^SCAN meta-generalized gradient approximation. J Phys Chem Lett. 2020;11(19):8208–8215. 32876454 10.1021/acs.jpclett.0c02405

[94] Losovyj YB, Makinistian L, Albanesi EA, Petukhov AG, Liu J, Galiy P, Dveriy OR, Dowben PA. The anisotropic band structure of layered In_4_Se_3_(001). J Appl Phys. 2008;104(8): Article 083713.

[95] Sheng Y, Wu Y, Yang J, Lu W, Villars P, Zhang W. Active learning for the power factor prediction in diamond-like thermoelectric materials. npj Comput Mater. 2020;6(1):Article 171.

[96] Hashemi M, Minbashi M, Ghorashi SMB, Ghobadi A, Ehsani MH, Heidariramsheh M, Hajjiah A. Electrical and optical characterization of sprayed In_2_S_3_ thin films as an electron transporting layer in high efficient perovskite solar cells. Sol Energy. 2021;215:356–366.

[97] Zhang J, Ishikawa D, Koza MM, Nishibori E, Song L, Baron AQR, Iversen BB. Dynamic lone pair expression as chemical bonding origin of giant phonon anharmonicity in thermoelectric InTe. Angew Chem Int Ed Engl. 2023;62(13): Article e202218458. 36696593 10.1002/anie.202218458

[98] Misra S, Barreteau C, Crivello J-C, Giordano VM, Castellan J-P, Sidis Y, Levinsky P, Hejtmánek J, Malaman B, Dauscher A, et al. Reduced phase space of heat-carrying acoustic phonons in single-crystalline InTe. Phys Rev Res. 2020;2(4): Article 043371.

[99] Duan B, Yang J, Salvador JR, He Y, Zhao B, Wang S, Wei P, Ohuchi FS, Zhang W, Hermann RP, et al. Electronegative guests in CoSb_3_. Energy Environ Sci. 2016;9(6):2090–2098.

[100] Ji J, Tang Q, Yao M, Yang H, Jin Y, Zhang Y, Xi J, Singh DJ, Yang J, Zhang W. Functional-unit-based material design: Ultralow thermal conductivity in thermoelectrics with linear triatomic resonant bonds. J Am Chem Soc. 2022;144(40):18552–18561. 36136764 10.1021/jacs.2c08062

[101] Wu J, Lin Y, Shu M, Liu Y, Ma Y, Lin G, Zhang C, Jiao P, Zhu F, Wu Y, et al. Uncovering the phonon spectra and lattice dynamics of plastically deformable InSe van der Waals crystals. Nat Commun. 2024;15(1):Article 6248. 39048583 10.1038/s41467-024-50249-5 PMC11269642

[102] Misra S, Léon A, Levinský P, Hejtmánek J, Lenoir B, Candolfi C. Enhanced thermoelectric performance of InTe through Pb doping. J Mater Chem C. 2021;9(40):14490–14496.

[103] Lin Z-S, Chen L, Wang L-M, Zhao J-T, Wu L-M. A promising mid-temperature thermoelectric material candidate: Pb/Sn-codoped In_4_Pb*_x_*Sn*_y_*Se_3_. Adv Mater. 2013;25(34):4800–4806. 23847133 10.1002/adma.201302038

[104] Rawat PK, Park H, Hwang J, Kim W. Low thermal conductivity and high thermoelectric performance in In_4_Se_3-*x*_ with phase-separated indium inclusions. J Electron Mater. 2016;46(3):1444–1450.

[105] Zhou M, Li J, Dong G, Gao S, Feng J, Liu R. Enhancement of thermoelectric performance for InTe by selective substitution and grain size modulation. Crystals. 2023;13(4):Article 601.

[106] Zhai Y, Zhang Q, Jiang J, Zhang T, Xiao Y, Yang S, Xu G. Thermoelectric properties of In_1.3-*x*_Sn*_x_*Se prepared by spark plasma sintering method. J Alloys Compd. 2013;553:270–272.

[107] Luo Y, Yang J, Liu M, Xiao Y, Fu L, Li W, Zhang D, Zhang M, Cheng Y. Multiple heteroatom induced carrier engineering and hierarchical nanostructures for high thermoelectric performance of polycrystalline In_4_Se_2.5_. J Mater Chem A. 2015;3(3):1251–1257.

[108] Misra S, Levinský P, Dauscher A, Medjahdi G, Hejtmánek J, Malaman B, Snyder GJ, Lenoir B, Candolfi C. Synthesis and physical properties of single-crystalline InTe: Towards high thermoelectric performance. J Mater Chem C. 2021;9(15):5250–5260.

[109] Back SY, Cho H, Kim Y-K, Byeon S, Jin H, Koumoto K, Rhyee J-S. Enhancement of thermoelectric properties by lattice softening and energy band gap control in Te-deficient InTe_1-*δ*_. AIP Adv. 2018;8(11): Article 115227.

[110] Rhyee J-S, Cho E, Lee KH, Lee SM, Kim SI, Kim H-S, Kwon YS, Kim SJ. Thermoelectric properties and anisotropic electronic band structure on the In_4_Se_3-*x*_ compounds. Appl Phys Lett. 2009;95(21): Article 212106.

[111] Alsharafi R, Zhan H, Shaheen N, Lu X, Wang G, Sun X, Zhou X. Se vacancy effect on the thermoelectric performance of Pb-doped In_4_Pb_0.01_Se_3-*x*_ polycrystalline. J Electron Mater. 2017;46(5):3131–3136.

[112] Zhu H, Wang G, Wang G, Zhou X, Lu X. The role of electronic affinity for dopants in thermoelectric transport properties of InTe. J Alloys Compd. 2021;869: Article 159224.

[113] He S-H, Liu P-F, Lin Z-X, Wang G-Q, Chen L, Wang Z-X, Wu L-M. The roles of Yb-substitution on thermoelectric properties of In_4-*x*_Yb*_x_*Se_3_. Acta Mater. 2015;101:16–21.

[114] Chen Y-C, Lin H, Wu L-M. High thermoelectric performance of polycrystalline In_4_Se_3-*δ*_(CuI)*_x_*: Synergistic effects of the Se-deficiency and CuI-doping. Inorg Chem Front. 2016;3(12):1566–1571.

[115] Rhyee J-S, Choi D. Thermoelectric properties of chlorine doped compounds of In_4_Se_2.7_Cl*_x_*. J Appl Phys. 2011;110(8): Article 083706.

[116] Abhari AS, Abdellahi M, Bahmanpour M. The effects of Sn-substitution on thermoelectric properties of In_4-*x*_Sn*_x_*Se_3_ ceramic. Ceram Int. 2016;42(5):5593–5599.

[117] Li G, Yang J, Luo Y, Xiao Y, Fu L, Liu M, Peng J. Improvement of thermoelectric properties of In_4_Se_3_ bulk materials with Cu nanoinclusions. J Am Ceram Soc. 2013;96(9):2703–2705.

[118] Yoo J, Kim J-i, Cho H-j, Choo S-s, Kim S-i, Lee K, Shin WH, Kim H-S, Roh JW. Electronic and thermal properties of si-doped InSe layered chalcogenides. J Korean Phys Soc. 2018;72(7):775–779.

[119] Lee KH, Oh M-W, Kim H-S, Shin WH, Lee K, Lim J-H, Kim J-i, Kim S-i. Enhanced thermoelectric transport properties of n-type InSe due to the emergence of the flat band by Si doping. Inorg Chem Front. 2019;6(6):1475–1481.

[120] Cho H, Roh JW, Park S, Kang SM, Park J, Kim S-i. Comparison of influence of intercalation and substitution of Cu on electrical and thermoelectric transport properties of InSe alloys. Phys Chem Chem Phys. 2024;26(9):7515–7521. 38357850 10.1039/d3cp05586h

[121] Lee SW, Kim T, Kim H-S, Park O, Kim DH, Kim S-i. Enhanced thermoelectric properties of InSe through simultaneous increase in electrical conductivity and Seebeck coefficient by Cl doping. J Mater Res Technol. 2022;19:2077–2083.

[122] Kim J-i, Kim H-S, Kim S-i. Electrical and thermal transport properties of S- and Te-doped InSe alloys. J Phys D Appl Phys. 2019;52(29): Article 295501.

[123] Cui J, Liu X, Zhang X, Li Y, Deng Y. Bandgap reduction responsible for the improved thermoelectric performance of bulk polycrystalline In_2-*x*_Cu*_x_*Se_3_ (*x* = 0-0.2). J Appl Phys. 2011;110(2): Article 023708.

[124] Cui J, Wang L, Du Z, Ying P, Deng Y. High thermoelectric performance of a defect in *α*-In_2_Se_3_-based solid solution upon substitution of Zn for In. J Mater Chem C. 2015;3(35):9069–9075.

[125] Song Z, Liu H, Du Z, Liu X, Cui J. Improvement of thermoelectric performance of *α*-In_2_Se_3_ upon S incorporation. Phys Status Solidi A. 2016;213(4):986–993.

[126] Park O, Lee SW, Kim S-i. Thermoelectric properties of Si-doped In_2_Se_3_ polycrystalline alloys. Ceramics. 2022;5(3):281–287.

[127] Chen YX, Yamamoto A, Takeuchi T. Doping effects of Mg for In on the thermoelectric properties of *β*-In_2_S_3_ bulk samples. J Alloys Compd. 2017;695:1631–1636.

[128] Feng J, Zhou M, Li J, Dong G, Gao S, Min E, Zhang C, He J, Sun R, Liu R. A boost of thermoelectric generation performance for polycrystalline InTe by texture modulation. Mater Horiz. 2023;10(8):3082–3089. 37218449 10.1039/d3mh00292f

[129] Zhu H, Zhang B, Wang G, Peng K, Yan Y, Zhang Q, Han X, Wang G, Lu X, Zhou X. Promoted high temperature carrier mobility and thermoelectric performance of InTe enabled by altering scattering mechanism. J Mater Chem A. 2019;7(19):11690–11698.

[130] Luo Y, Yang J, Li G, Liu M, Xiao Y, Fu L, Li W, Zhu P, Peng J, Gao S, et al. Enhancement of the thermoelectric performance of polycrystalline In_4_Se_2.5_ by copper intercalation and bromine substitution. Adv Energy Mater. 2013;4(2):Article 1300599.

[131] Zhai Y, Zhang Q, Jiang J, Zhang T, Xiao Y, Yang S, Xu G. Thermoelectric performance of the ordered In_4_Se_3_-In composite constructed by monotectic solidification. J Mater Chem A. 2013;1(31):8844–8847.

[132] Mannu P, Palanisamy M, Bangaru G, Ramakrishnan S, Ramcharan M, Kandasami A. Structural and thermoelectric properties of Se doped In_2_Te_3_ thin films. AIP Adv. 2018;8(11): Article 115015.

[133] Sowjanya V. A significant enhancement in thermoelectric power factor of In_2_Te_3_ thin films by Se doping and Te composition tuning. Curr Appl Phys. 2022;39:56–61.

[134] Qu L, Yang C, Luo Y, Du Z, Li C, Cui J. Band structure and phonon transport engineering realizing remarkable improvement in thermoelectric performance of Cu_2_SnSe_4_ incorporated with In_2_Te_3_. ACS Appl Mater Interfaces. 2022;14(40):45628–45635. 36190823 10.1021/acsami.2c14688

[135] Shen J, Zhang X, Lin S, Li J, Chen Z, Li W, Pei Y. Vacancy scattering for enhancing the thermoelectric performance of CuGaTe_2_ solid solutions. J Mater Chem A. 2016;4(40):15464–15470.

[136] Tan G, Zeier WG, Shi F, Wang P, Snyder GJ, Dravid VP, Kanatzidis MG. High thermoelectric performance SnTe-In_2_Te_3_ solid solutions enabled by resonant levels and strong vacancy phonon scattering. Chem Mater. 2015;27(22):7801–7811.

[137] Sun H, Lu X, Chi H, Morelli DT, Uher C. Highly efficient (In_2_Te_3_)*_x_*(GeTe)_3−3*x*_ thermoelectric materials: A substitute for TAGS. Phys Chem Chem Phys. 2014;16(29):15570–15575. 24953478 10.1039/c4cp01294a

[138] Pei Y, Morelli DT. Vacancy phonon scattering in thermoelectric In_2_Te_3_-InSb solid solutions. Appl Phys Lett. 2009;94(12): Article 122112.

[139] Hsin C-L, Huang C-W, Wu M-H, Cheng S-Y, Pan R-C. Synthesis and thermoelectric properties of indium telluride nanowires. Mater Res Bull. 2019;112:61–65.

[140] Sanchela AV, Thakur AD, Tomy CV. Improvement in thermoelectric properties by tailoring at In and Te site in In_2_Te_5_. J Electron Mater. 2016;45(11):5540–5545.

[141] Wei T-R, Jin M, Wang Y, Chen H, Gao Z, Zhao K, Qiu P, Shan Z, Jiang J, Li R, et al. Exceptional plasticity in the bulk single-crystalline van der Waals semiconductor InSe. Science. 2020;369(6503):542–545. 32732421 10.1126/science.aba9778

[142] Andharia E, Alqurashi H, Pandit A, Hamad B. Self-consistent phonon calculations and quartic anharmonic lattice thermal conductivity in 2D InS monolayer. AIP Adv. 2024;14(1): Article 015223.

[143] Li L, Peng C, Chen J, Ma Z, Chen Y, Li S, Wang J, Wang C. Study the effect of alloying on the phase transition behavior and thermoelectric properties of Ag_2_S. J Alloys Compd. 2021;886: Article 161241.

[144] Zhang F, Ma B, Luo Y, Zhu L, Wang W, Shi Y, Jia B, Ge Z-H, Yang Z, Wu D, et al. Promoted thermoelectric performance in cubic-phase GeTe via grain-boundary phase elimination under phase diagram guidance. Energy Environ Sci. 2024;17(22):8691–8701.

[145] Chae S, Chae SS, Choi M, Park HM, Chang H, Lee J-O, Lee TI. Blocking of the 1T-to-2H phase transformation of chemically exfoliated transition metal disulfides by using a “lattice lock”. Nano Energy. 2019;56:65–73.

[146] Luo C, Dong Z, Xu T, Yang X, Zhang H, Bi H, Wang C, Sun L, Chu J, Wu X. Tailoring the phase transition of silver selenide at the atomistic scale. Nanoscale. 2022;14(43):16077–16084. 36124640 10.1039/d2nr04248g

[147] Ramirez DC, Macario LR, Cheng X, Cino M, Walsh D, Tseng Y-C, Kleinke H. Large scale solid state synthetic technique for high performance thermoelectric materials: Magnesium-silicide-stannide. ACS Appl Energy Mater. 2020;3(3):2130–2136.

[148] Kim G, Lee H, Kim J, Roh JW, Lyo I, Kim B-W, Lee KH, Lee W. Up-scaled solid state reaction for synthesis of doped Mg_2_Si. Scr Mater. 2017;128:53–56.

[149] Wang W, Sun Y, Feng Y, Qin H, Zhu J, Guo F, Cai W, Sui J. High thermoelectric performance bismuth telluride prepared by cold pressing and annealing facilitating large scale application. Mater Today Phys. 2021;21: Article 100522.

[150] Madavali B, Kim H-S, Lee K-H, Isoda Y, Gascoin F, Hong S-J. Large scale production of high efficient and robust p-type Bi-Sb-Te based thermoelectric materials by powder metallurgy. Mater Des. 2016;112:485–494.

[151] Song K, Tanvir ANM, Bappy MO, Zhang Y. New directions for thermoelectrics: A roadmap from high-throughput materials discovery to advanced device manufacturing. Small Sci. 2024;5(3):Article 2300359. 10.1002/smsc.202300359 PMC12245080 40655129

[152] Deng T, Qiu P, Yin T, Li Z, Yang J, Wei T, Shi X. High-throughput strategies in the discovery of thermoelectric materials. Adv Mater. 2024;36(13):Article 2311278. 10.1002/adma.202311278 38176395

[153] Liu L, Yao M, Wang Y, Jin Y, Ji J, Luo H, Cao Y, Xiong Y, Sheng Y, Li X, et al. The MatHub-3d first-principles repository and the applications on thermoelectrics. Mater Genome Eng Adv. 2024;2(1): Article e21.

[154] Back SY, Kim Y-K, Cho H, Han M-K, Kim S-J, Rhyee JS. Temperature-induced Lifshitz transition and charge density wave in InTe_1-*δ*_ thermoelectric materials. ACS Appl Energy Mater. 2020;3(4):3628–3636.

[155] Wang S, Qiu Y, Chang C, Zhao L-D. A route to high thermoelectric performance of lead chalcogenides: Enhancing carrier mobility. Sci China Mater. 2024;68(3):780–784.

[156] Wang H, He Q, Gao X, Shang Y, Zhu W, Zhao W, Chen Z, Gong H, Yang Y. Multifunctional high entropy alloys enabled by severe lattice distortion. Adv Mater. 2023;36(17):Article 2305453. 10.1002/adma.202305453 37561587

[157] Guan X, Ouyang J. Enhancement of the Seebeck coefficient of organic thermoelectric materials via energy filtering of charge carriers. CCS Chem. 2021;3(10):2415–2427.

[158] Ahn K, Cho E, Rhyee J-S, Kim SI, Hwang S, Kim H-S, Lee SM, Lee KH. Improvement in the thermoelectric performance of the crystals of halogen-substituted In_4_Se_3-*x*_H_0.03_ (H = F, Cl, Br, I): Effect of halogen-substitution on the thermoelectric properties in In_4_Se_3-*x*_. J Mater Chem. 2012;22(12):5730–5736.

[159] Kim JH, Kim MJ, Oh S, Rhyee J-S, Park S-D, Ahn D. Thermoelectric properties and chlorine doping effect of In_4_Pb_0.01_Sn_0.03_Se_2.9_Cl*_x_* polycrystalline compounds. Dalton Trans. 2015;44(7):3185–3189. 25579326 10.1039/c4dt03432e

[160] Sanchela AV, Thakur AD, Tomy CV. Anisotropic thermal conductivity and thermopower of In_2_Te_5_ single crystal. AIP Conf Proc. 2014;1591(1):1392–1394.

[161] Nasr TB, Abdallah HB, Bennaceur R. First-principles study of the electronic and the optical properties of In_6_Se_7_ compound. Physica B Condens Matter. 2010;405(16):3427–3432.

[162] Abdallah HB, Bennaceur R. First-principles calculations of the electronic and optical properties of In_6_S_7_ compound. Physica B Condens Matter. 2009;404(2):194–198.

[163] Nagesha DK, Liang X, Mamedov AA, Gainer G, Eastman MA, Giersig M, Song JJ, Ni T, Kotov NA. In_2_S_3_ nanocolloids with excitonic emission: In_2_S_3_ vs CdS comparative study of optical and structural characteristics. J Phys Chem B. 2001;105(31):7490–7498.

