# Supplementary material for: Structural Characteristics and Recent Advances in Thermoelectric Binary Indium Chalcogenides
Source: Research (Wash D C). 2025 Jun 10;8:0727. doi: 10.34133/research.0727 (PMC12150782; doi:10.34133/research.0727)
Supplement: Supplementary 1 — Figs. S1 to S10 [file research.0727.f1.docx]

**Supplementary Materials:**

**Structural characteristics and recent advances in thermoelectric binary indium chalcogenides**

**Yasong Wu^1, #^, Binjie Zhou^1, #^, Lu Liu^1^, Shengnan Dai^1,^** ^*^**, Lirong Song^1,^** ^*^**, Jiong Yang^1,^** ^*^

^1^Materials Genome Institute, Shanghai Engineering Research Center for Integrated Circuits and Advanced Display Materials, Shanghai University, Shanghai 200444, China.

^#^These authors contributed equally to this work.

^*^**Address Correspondence to:** Dr. Shengnan Dai, [musenc@shu.edu.cn](mailto:musenc@shu.edu.cn); Dr. Lirong Song, [lirongsong@shu.edu.cn](mailto:lirongsong@shu.edu.cn) and Prof. Jiong Yang, [jiongy@t.shu.edu.cn](mailto:jiongy@t.shu.edu.cn).

**
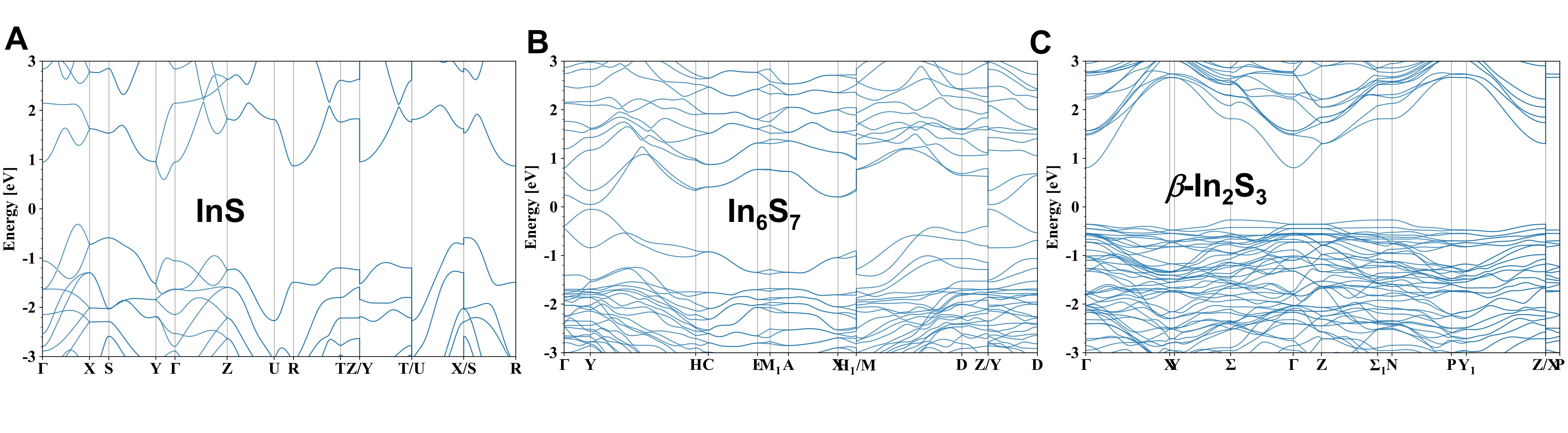
**

**Fig. S1.** Electronic band structures of In-S compounds calculated with PBEsol+HSE06 functionals.


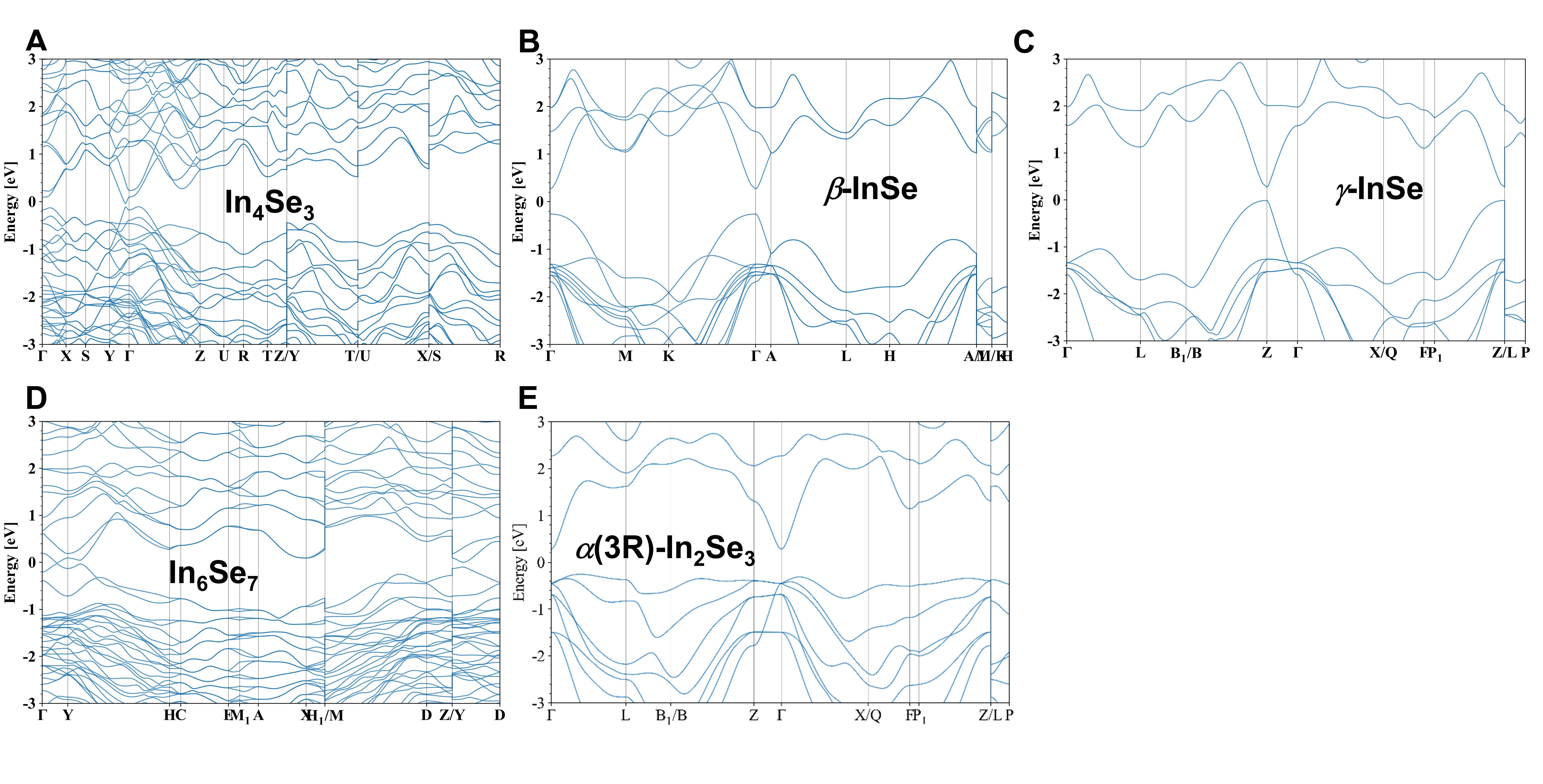


**Fig. S2.** Electronic band structures of In-Se compounds calculated with PBEsol+HSE06 functionals.


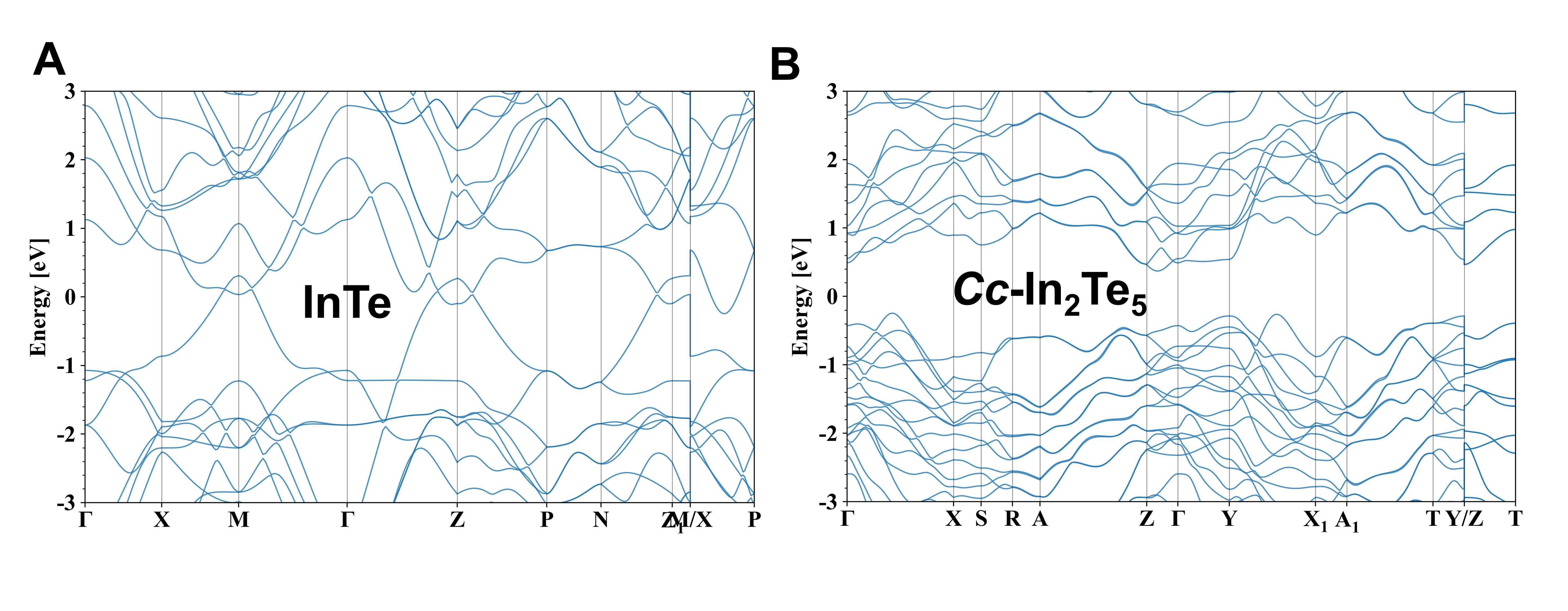


**Fig. S3.** Electronic band structures of In-Te compounds calculated with PBEsol+HSE06 functionals.





**Fig. S4.** Electronic band structure of InTe calculated using r^2^SCAN functional.


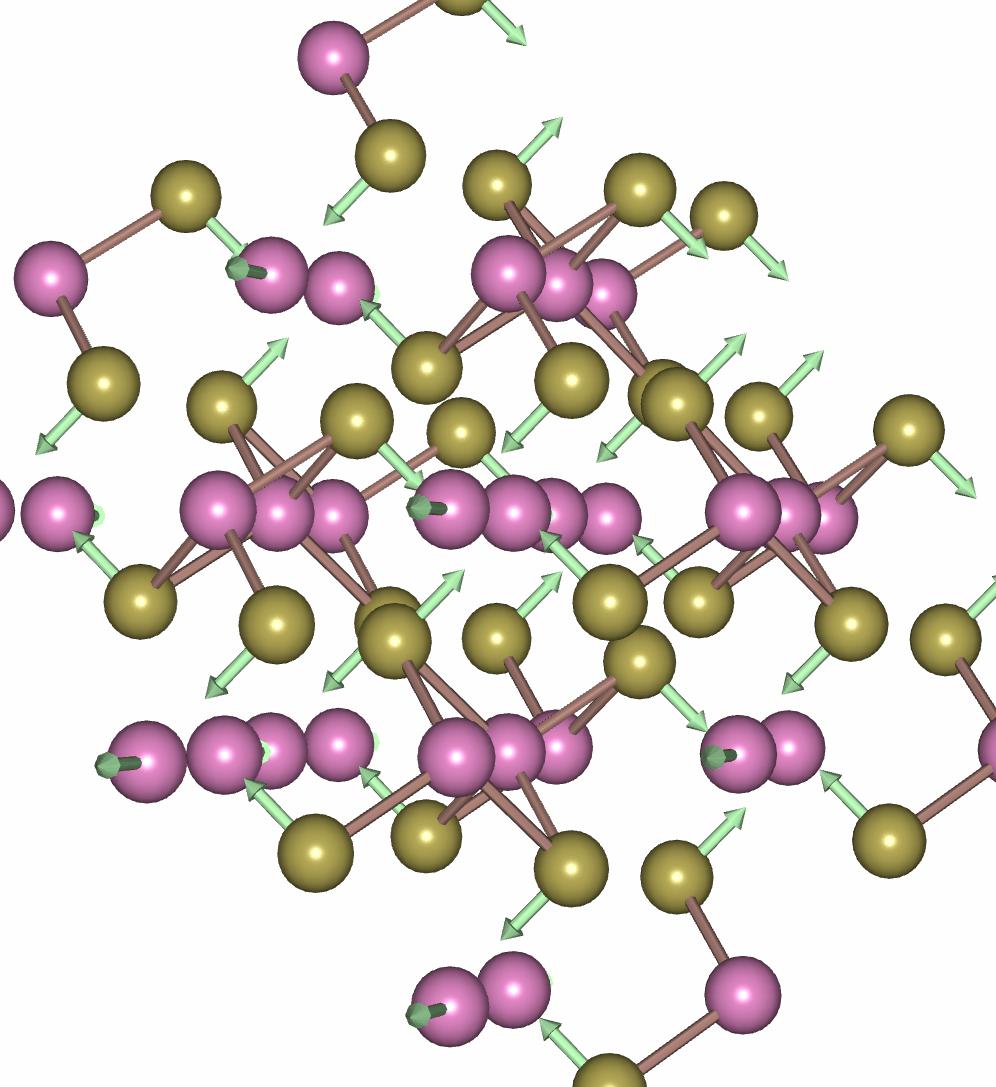

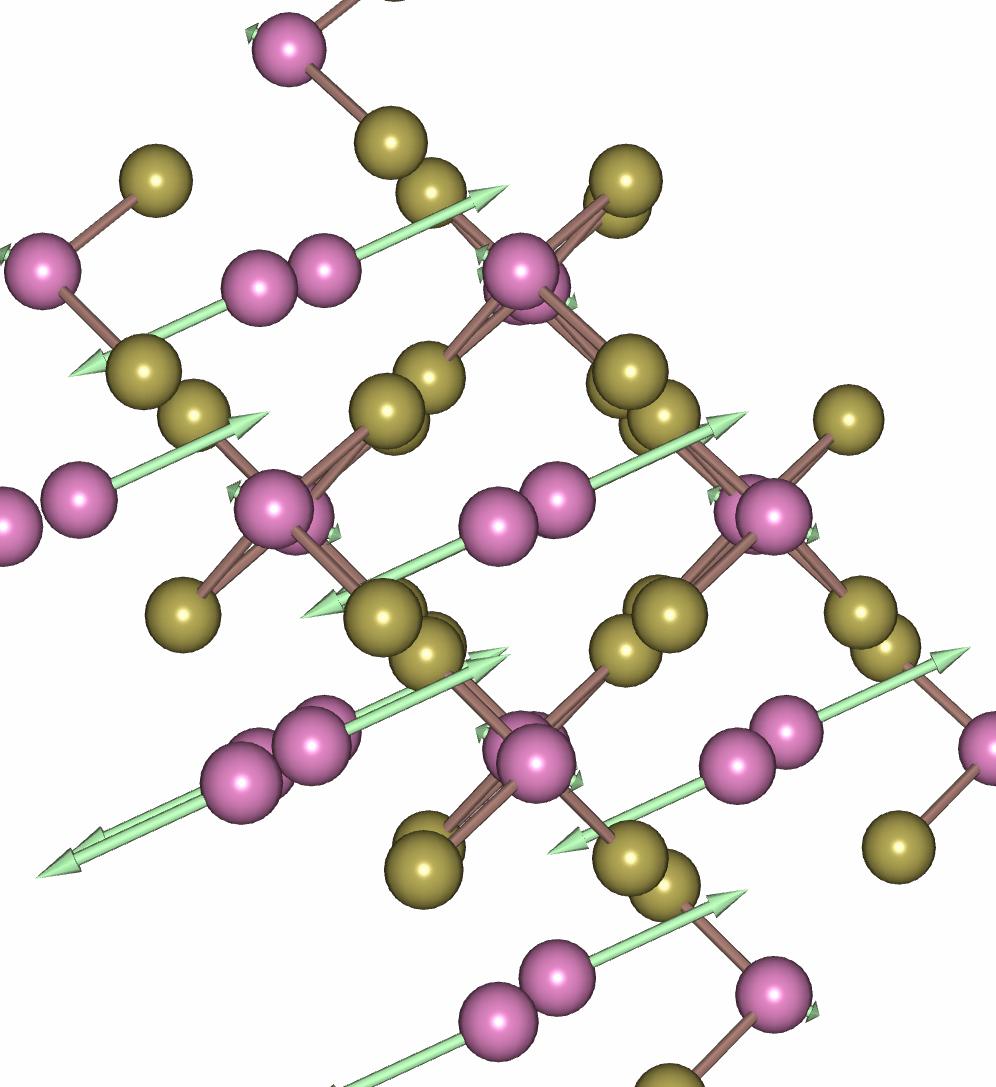


**Fig. S5.** Vibrational modes of 1.78 and 2.04 THz in InTe at Γ point. In and Te atoms are marked in purple and dark yellow, respectively.

**
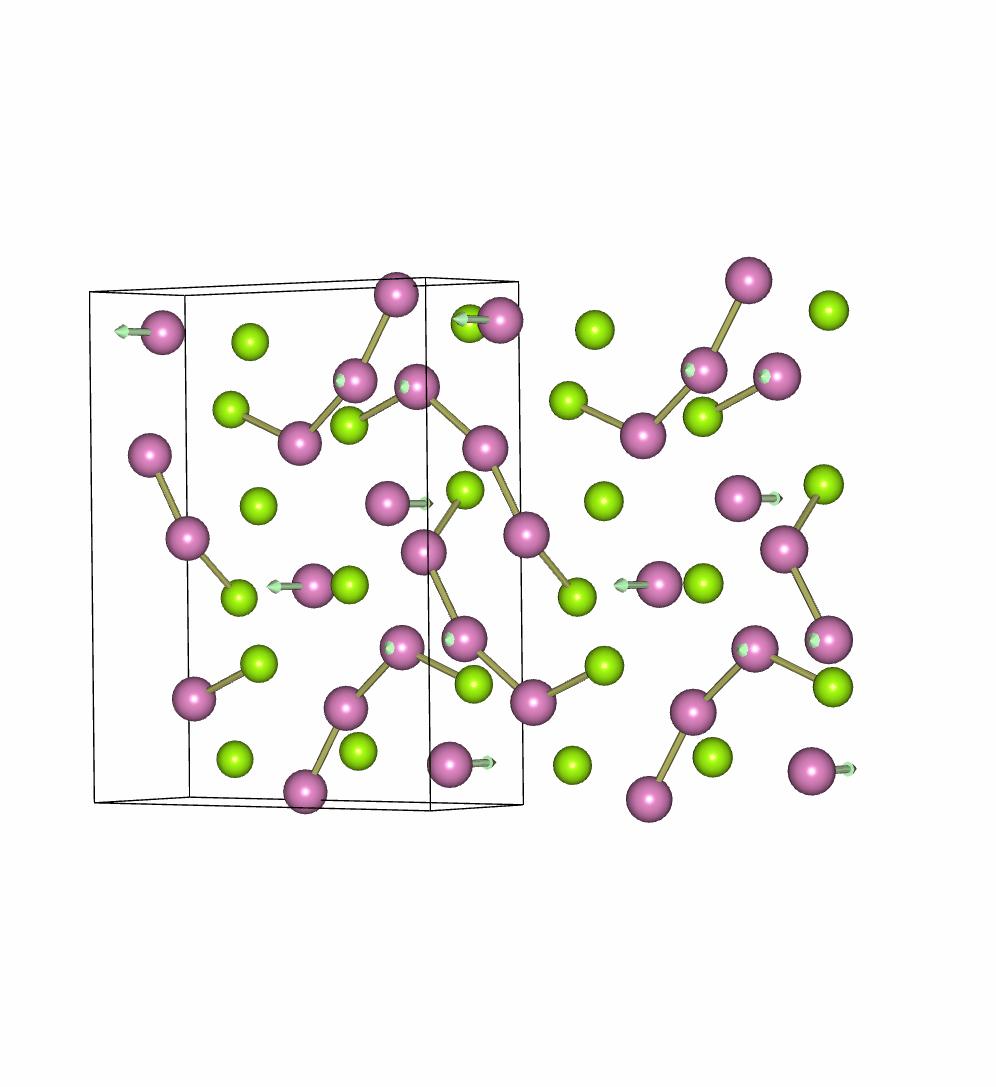

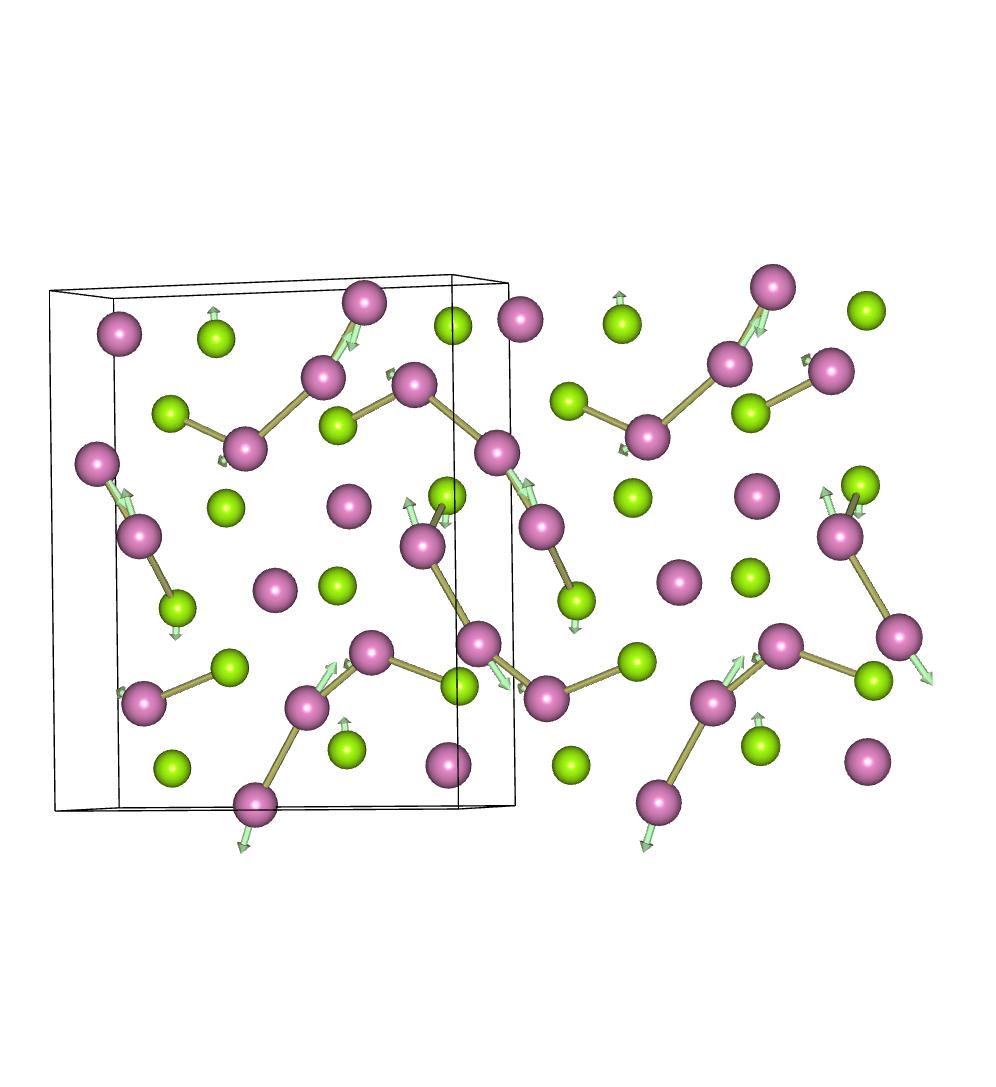
**

**Fig. S6.** Vibrational modes of 2.08 and 7.04 THz in In_4_Se_3_ at Γ point. In and Se atoms are marked in purple and green, respectively.


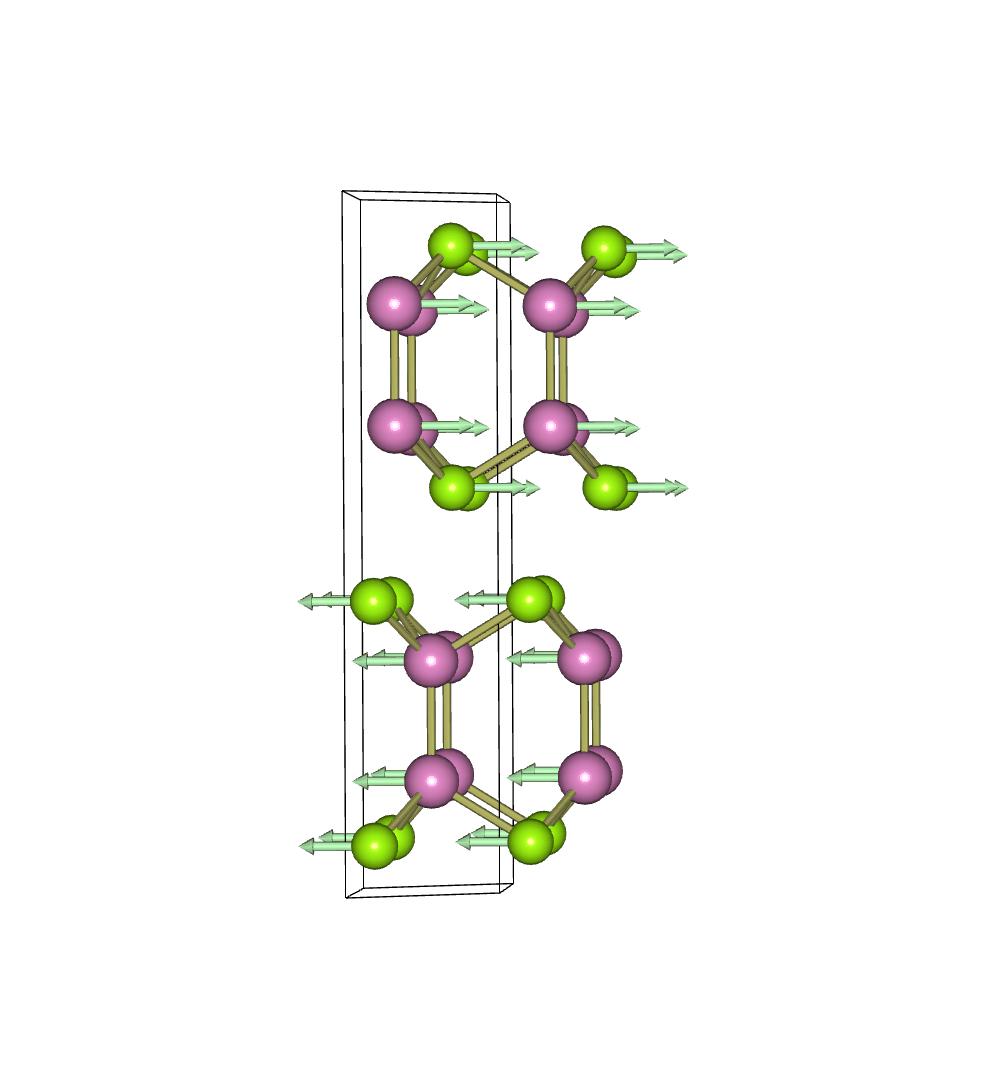

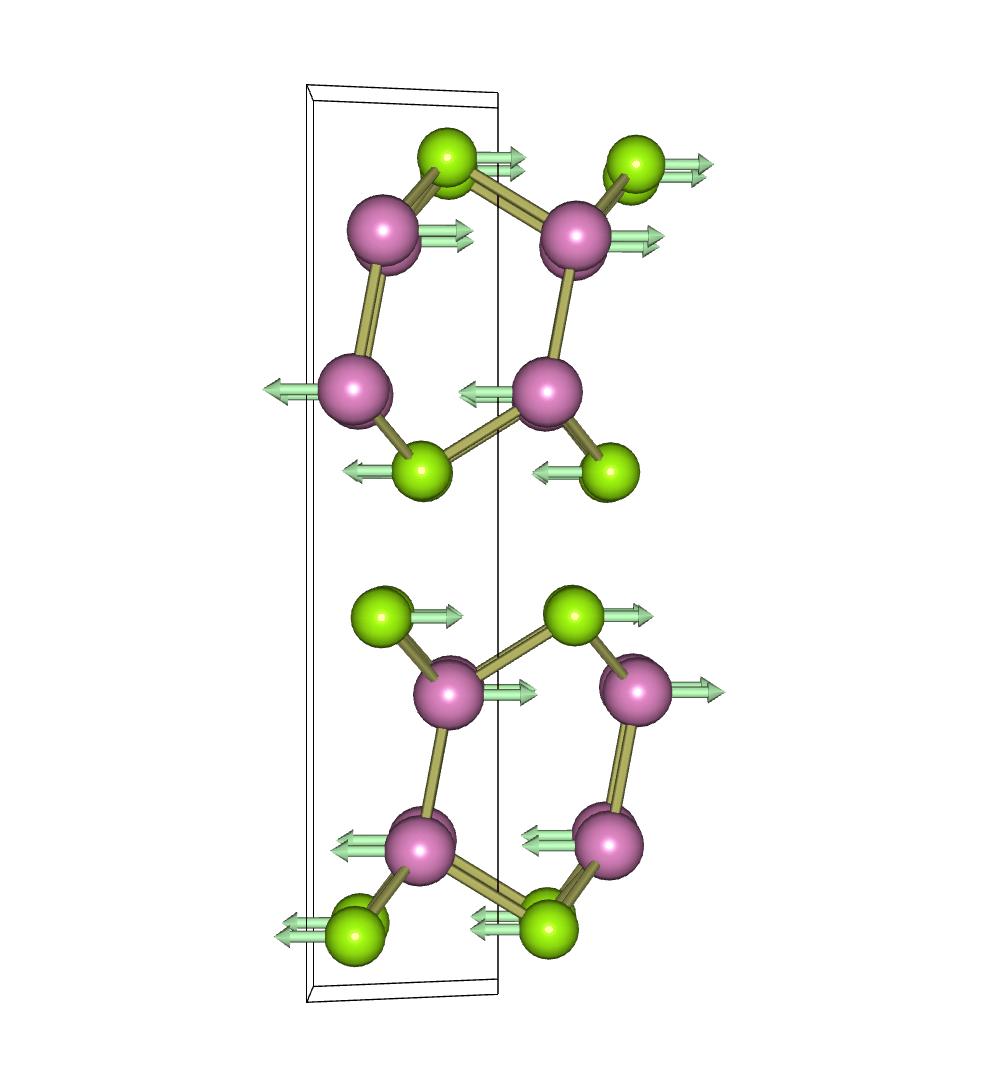

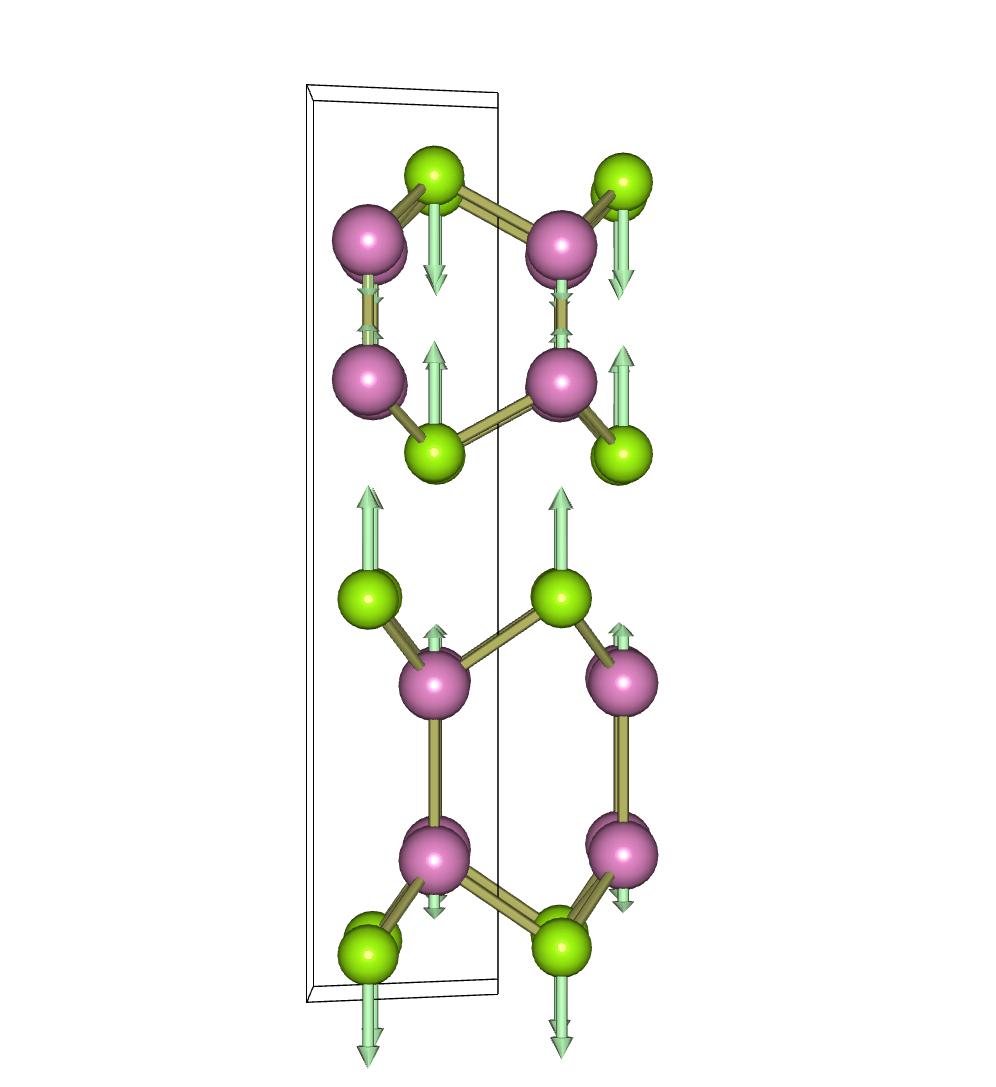

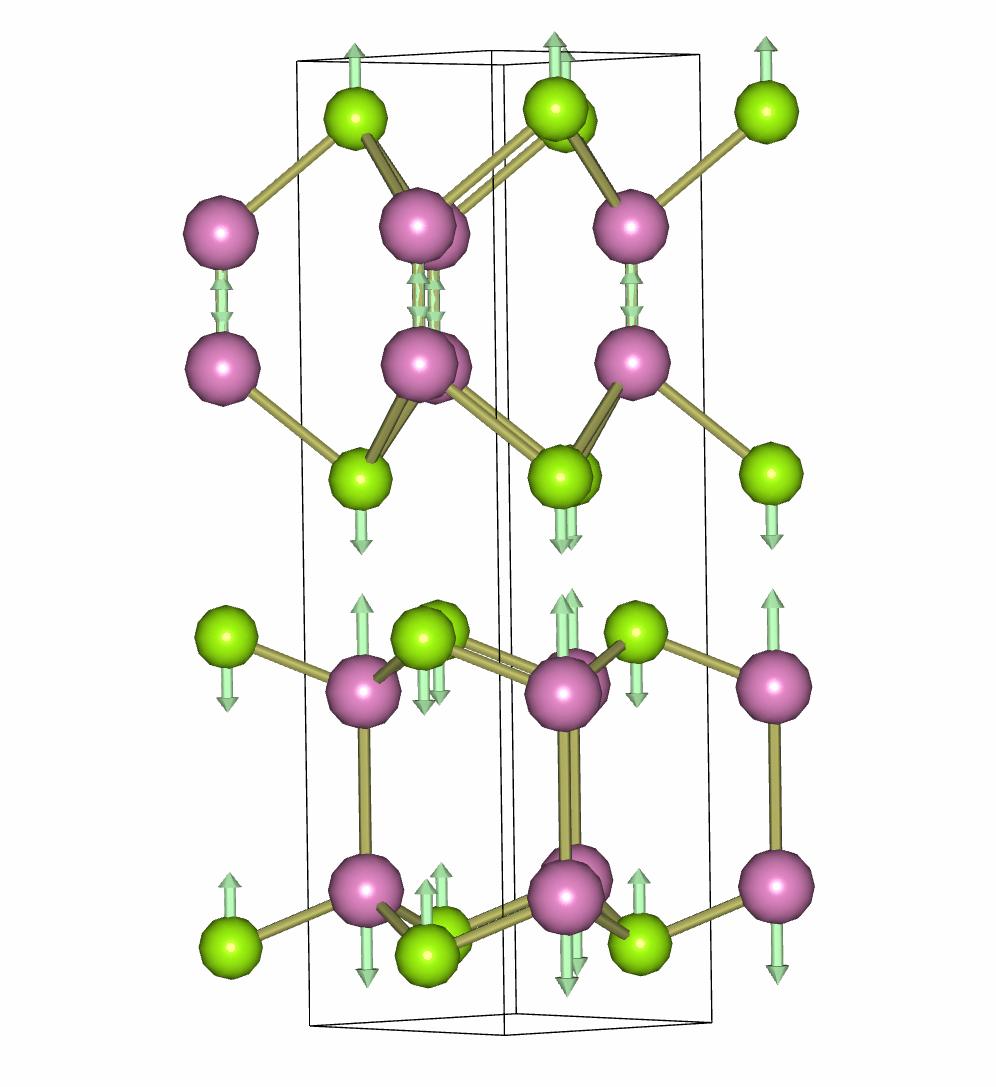


**Fig. S7.** Vibrational modes of 0.96, 1.23, 3.54, and 7.52 THz in *β*-InSe at Γ point. In and Se atoms are marked in purple and green, respectively.


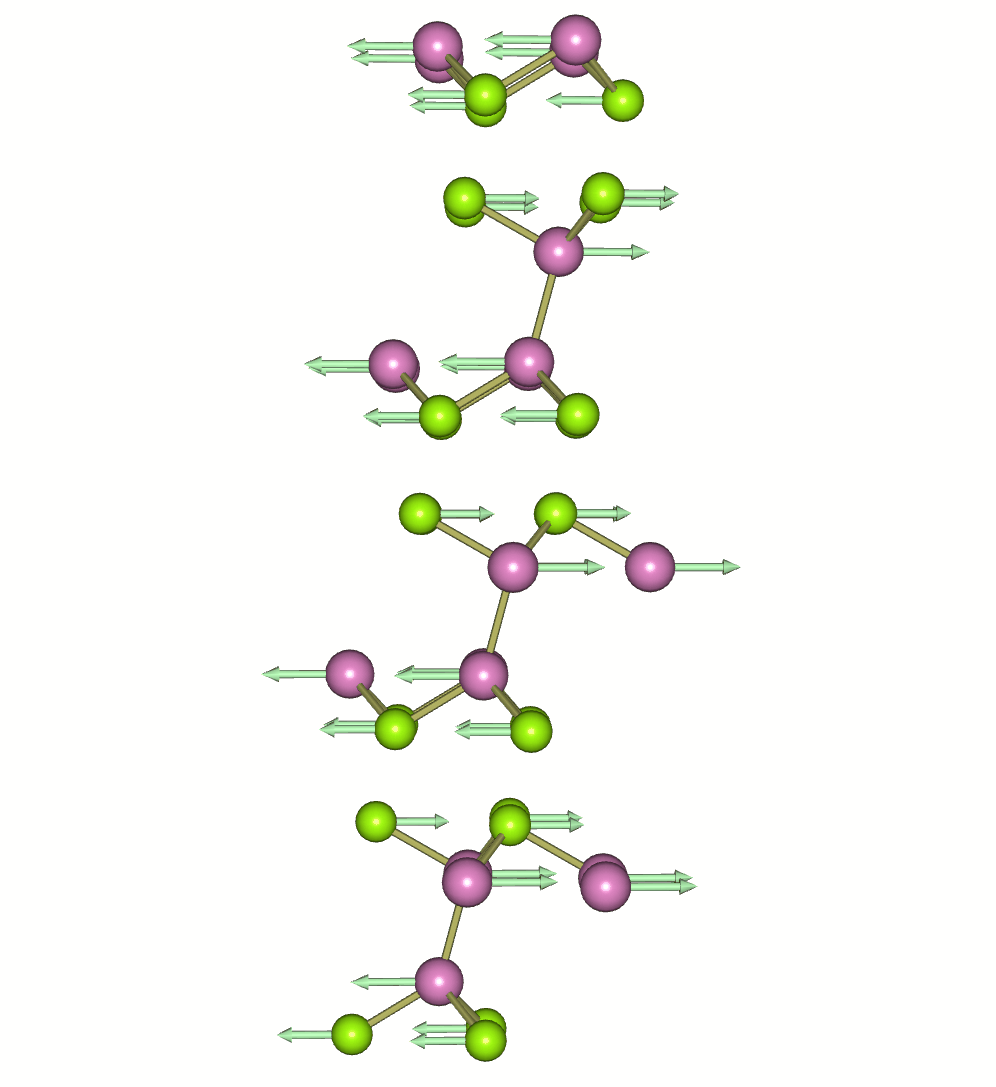

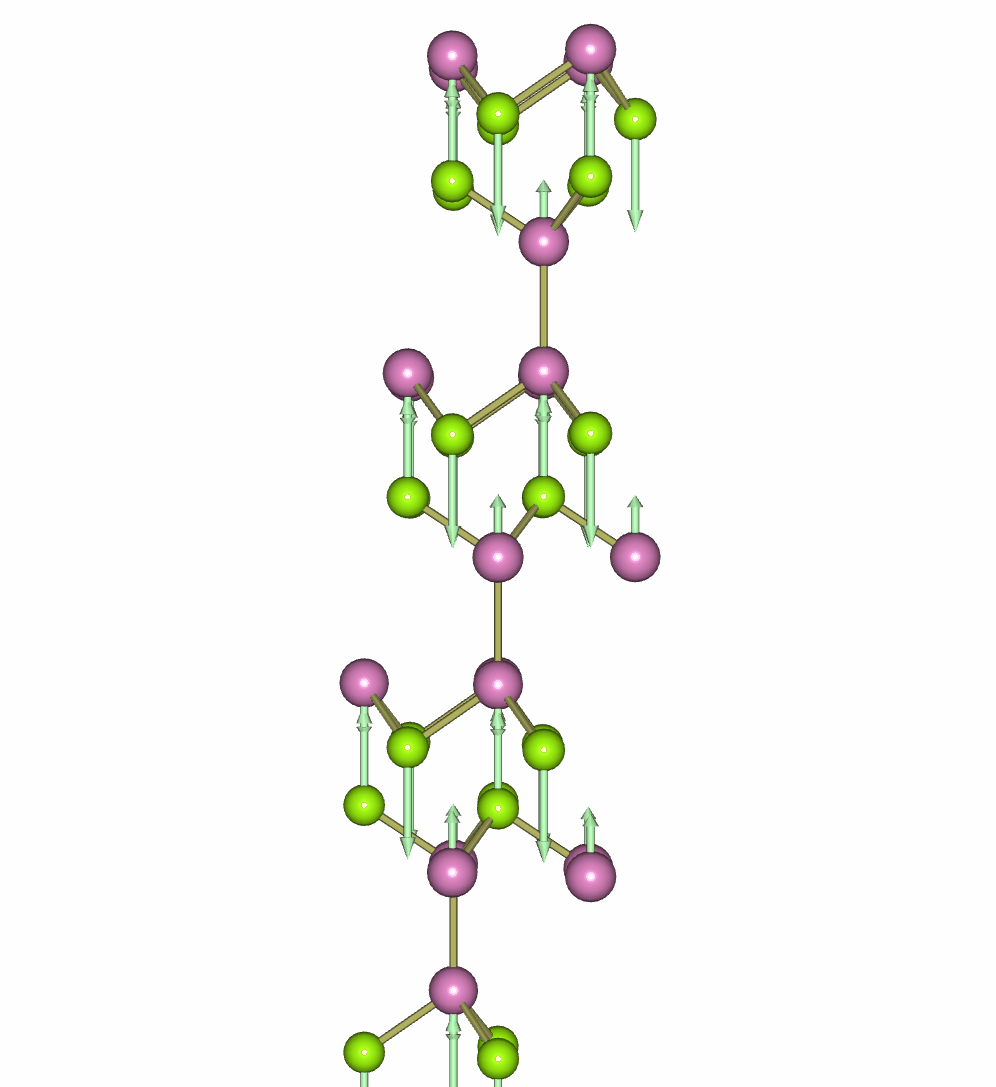

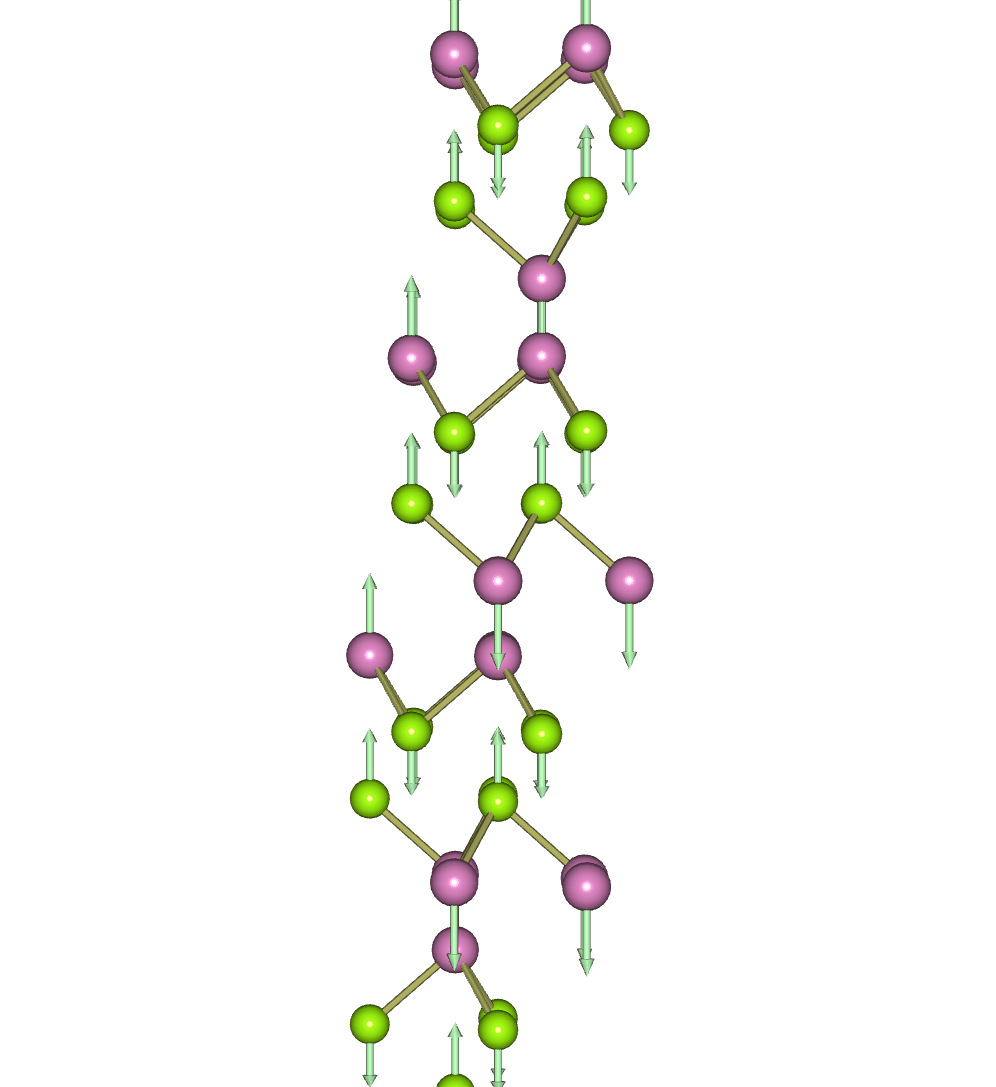


**Fig. S8.** Vibrational modes of 1.34, 4.30, and 7.38 THz in *γ*-InSe at Γ point. In and Se atoms are marked in purple and green, respectively.


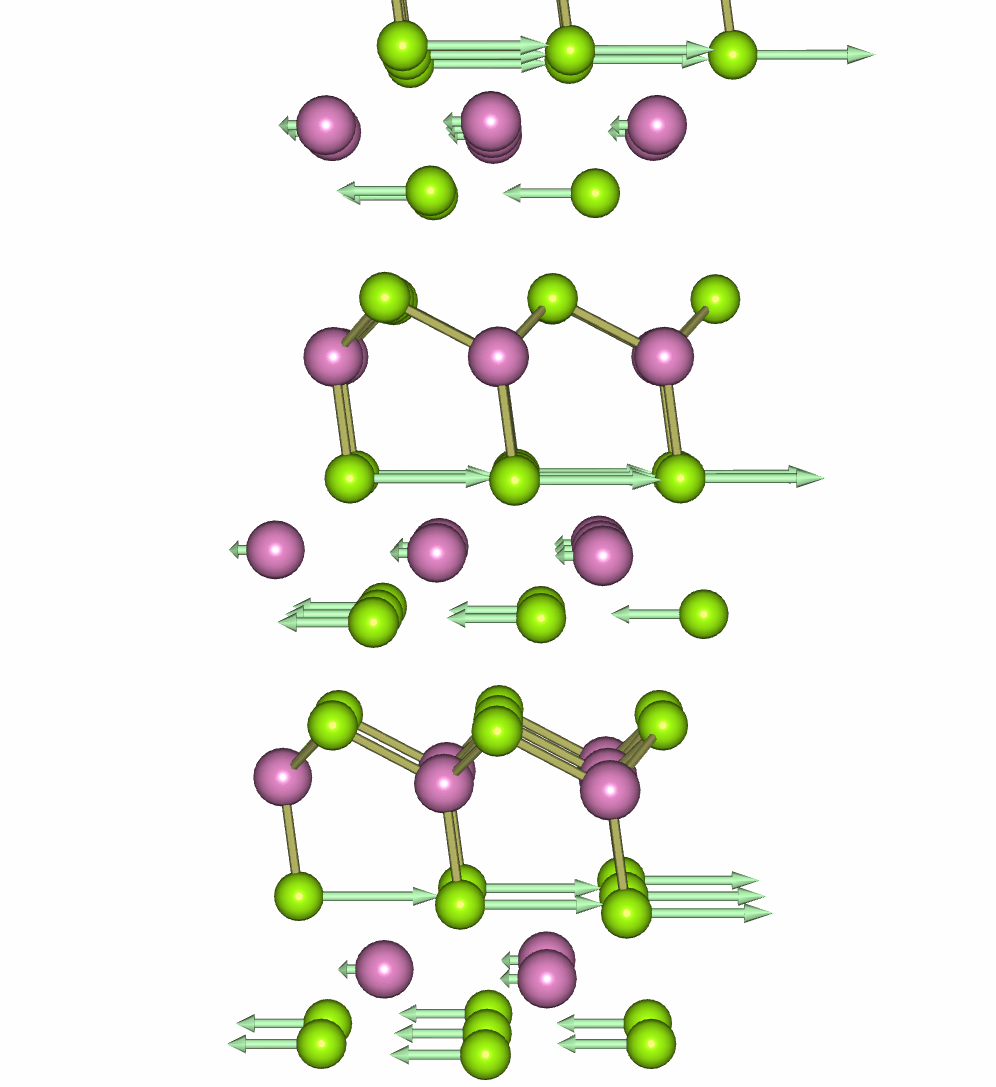

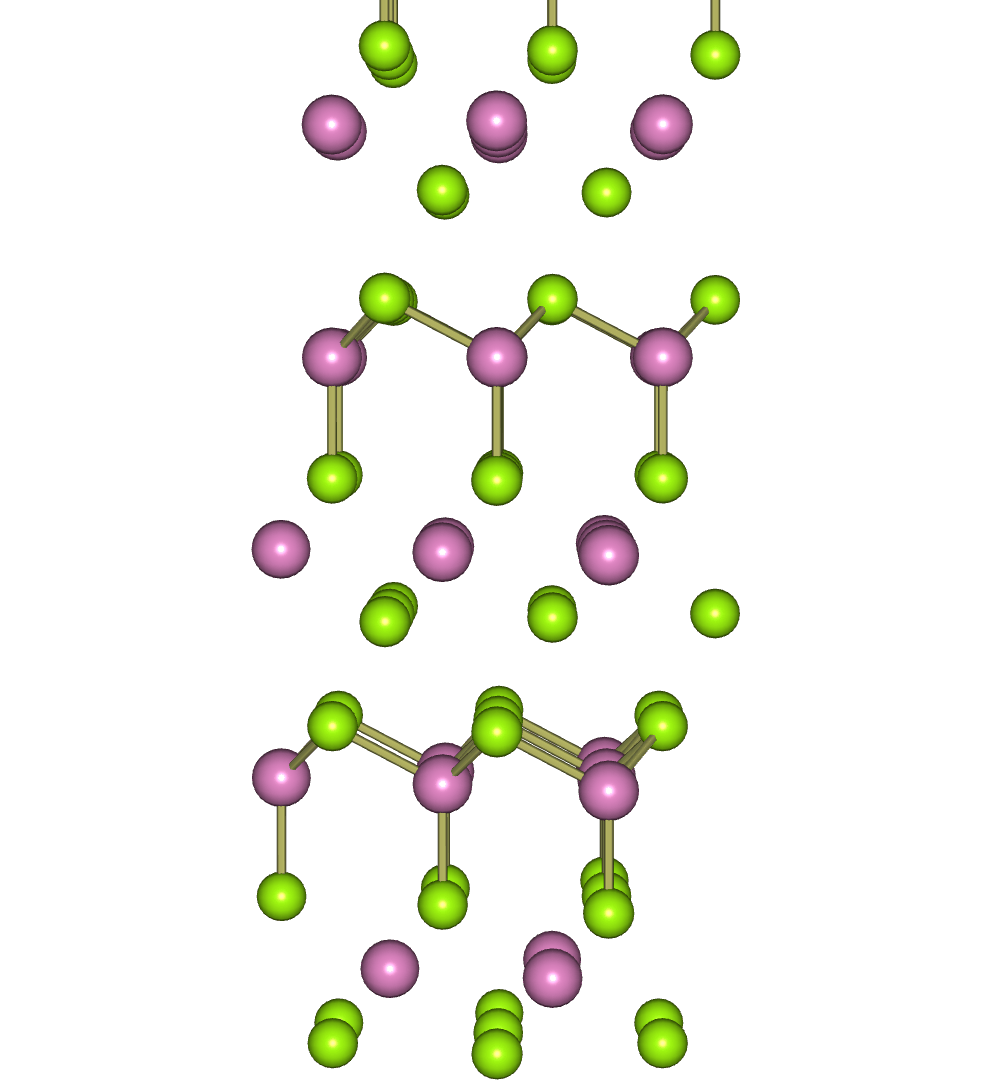

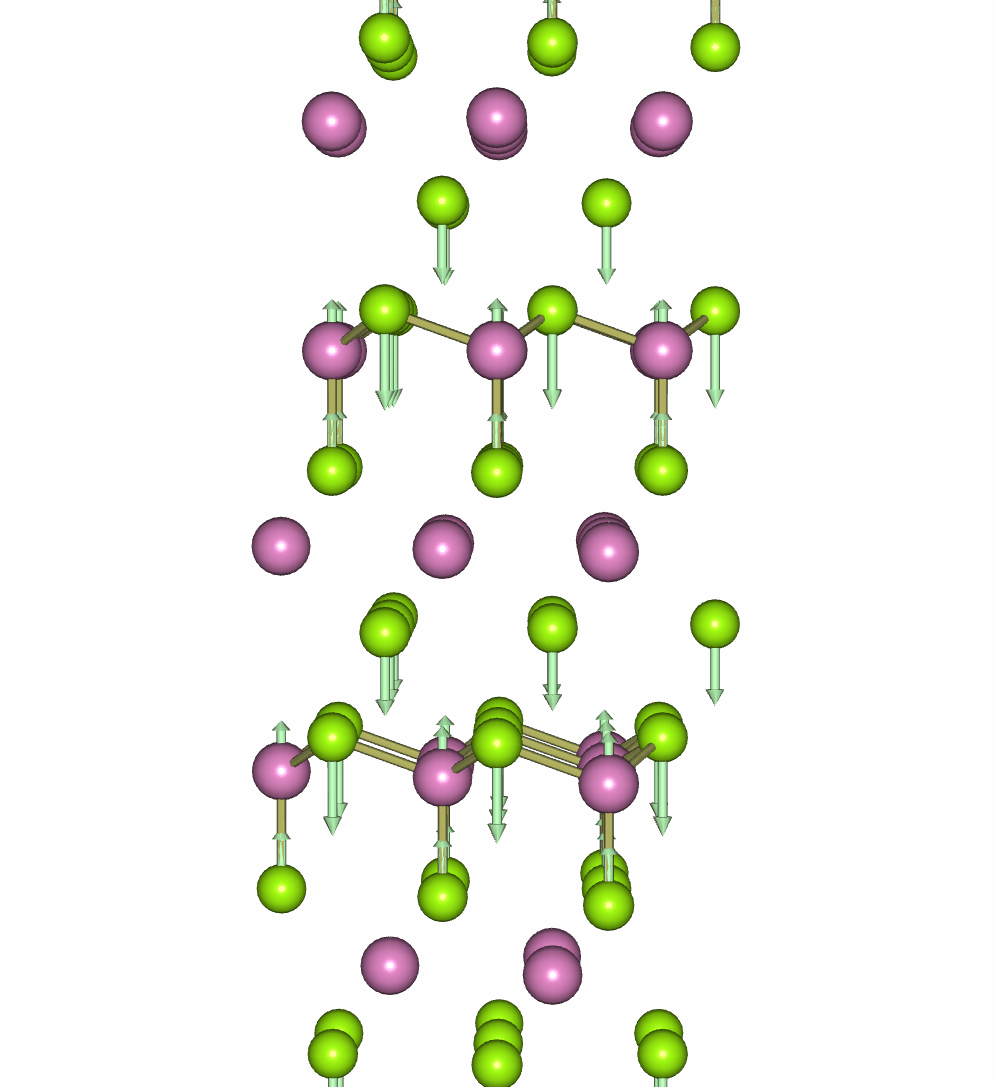

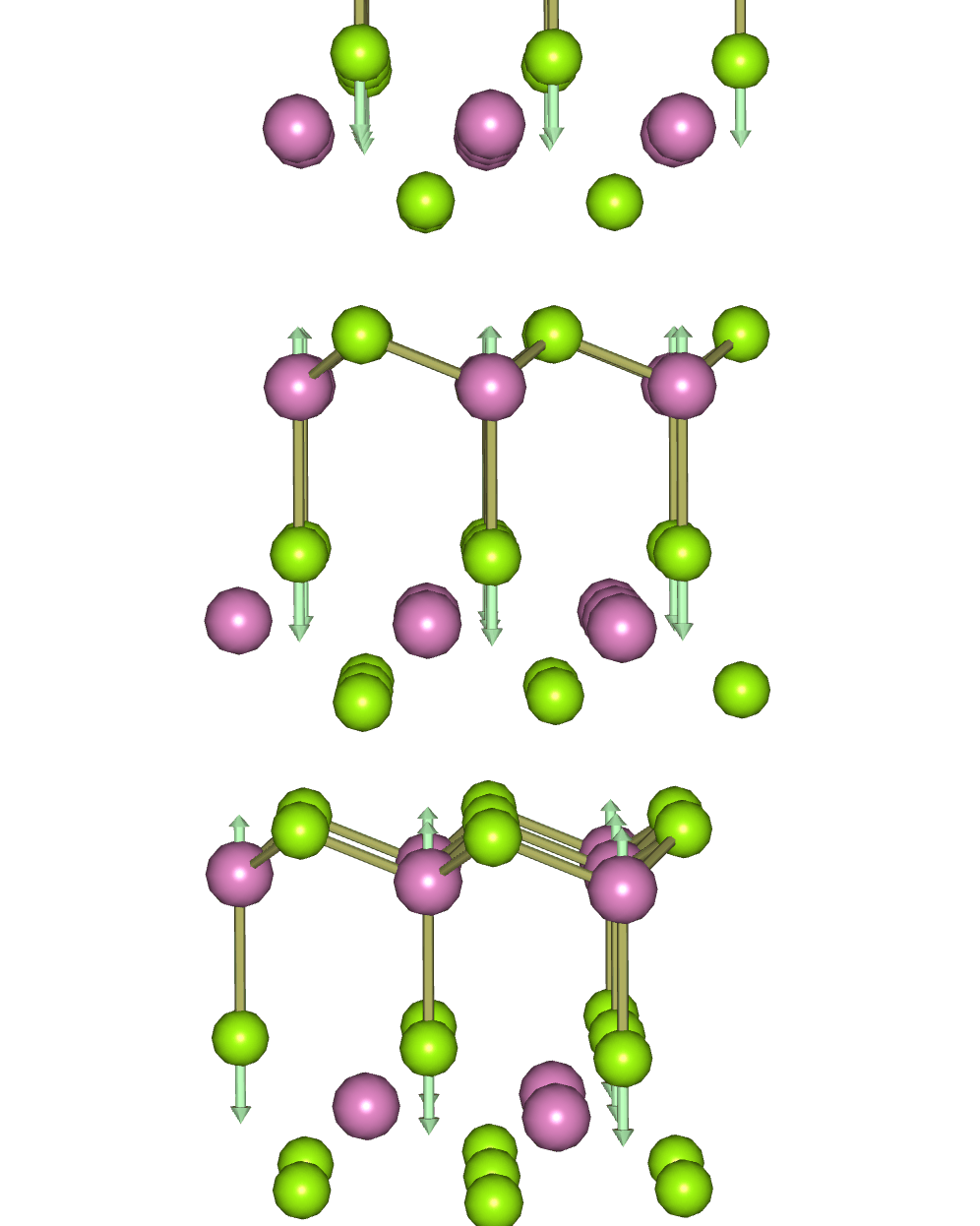


**Fig. S9.** Vibrational modes of 3.52, 3.78, 5.60, and 7.68 THz in *α*(3R)-In_2_Se_3_ at Γ point. In and Se atoms are marked in purple and green, respectively.


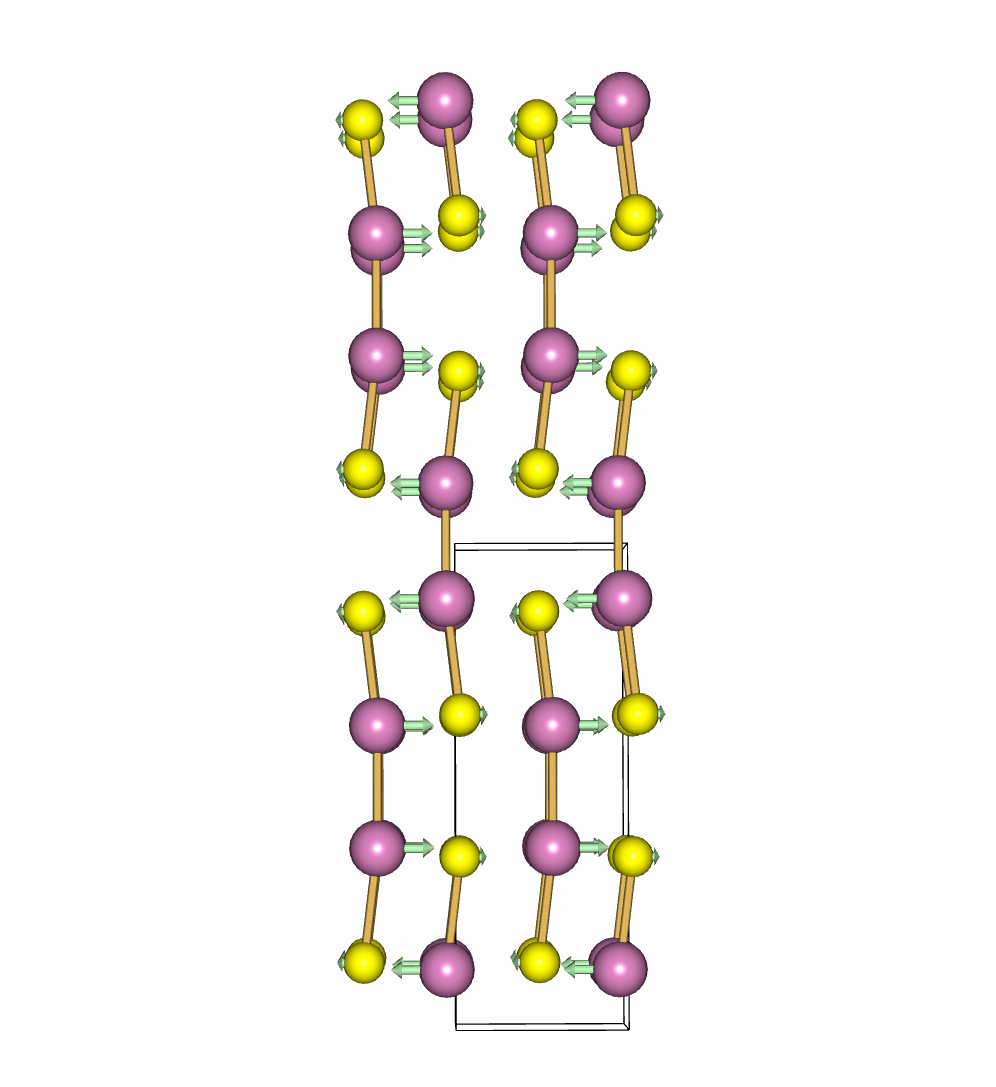

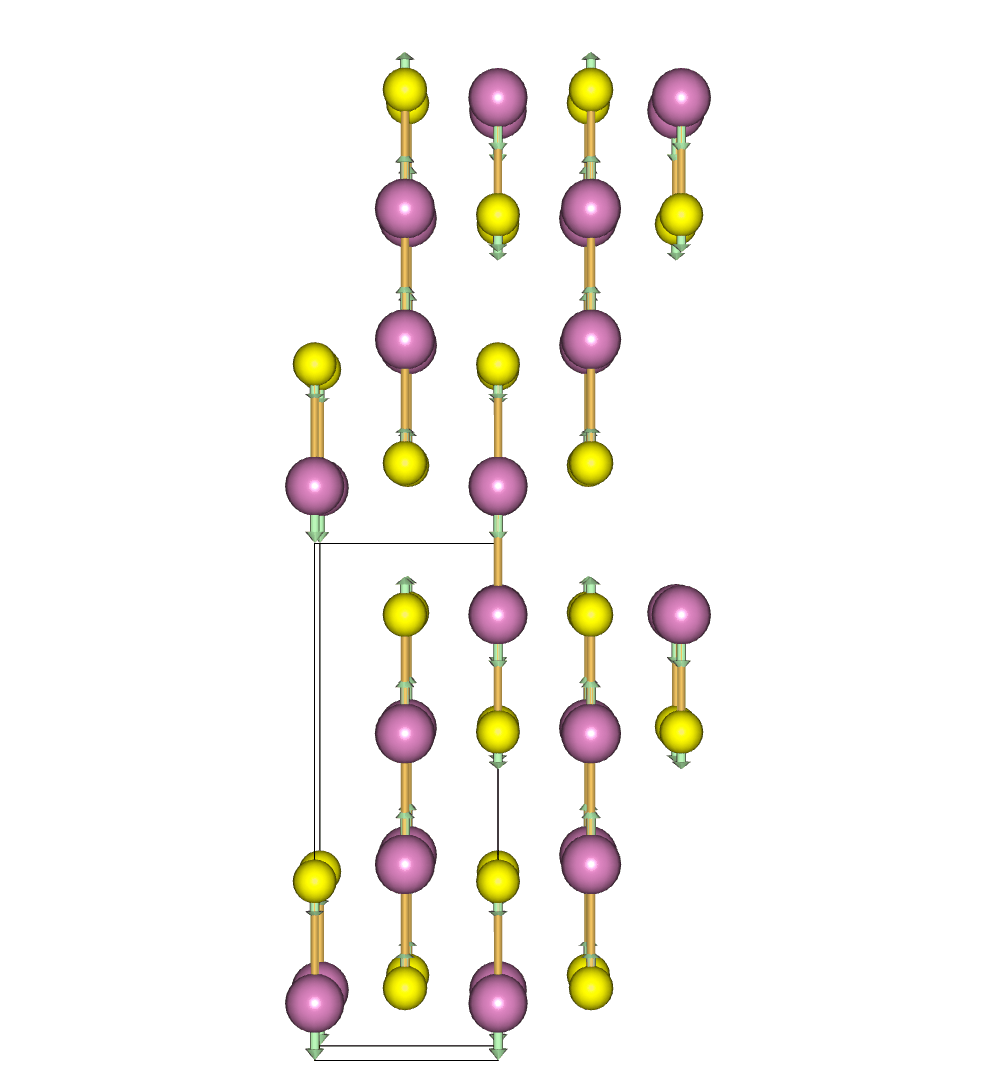

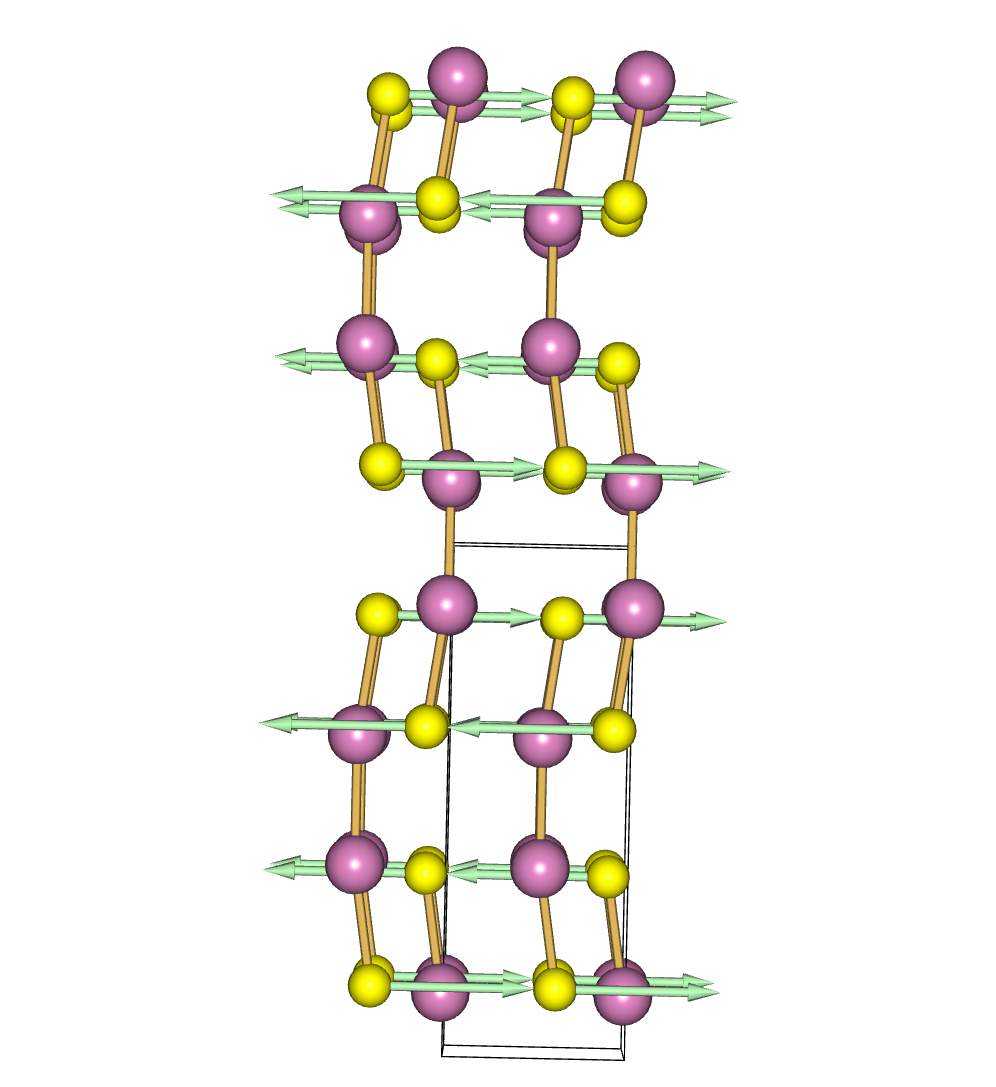

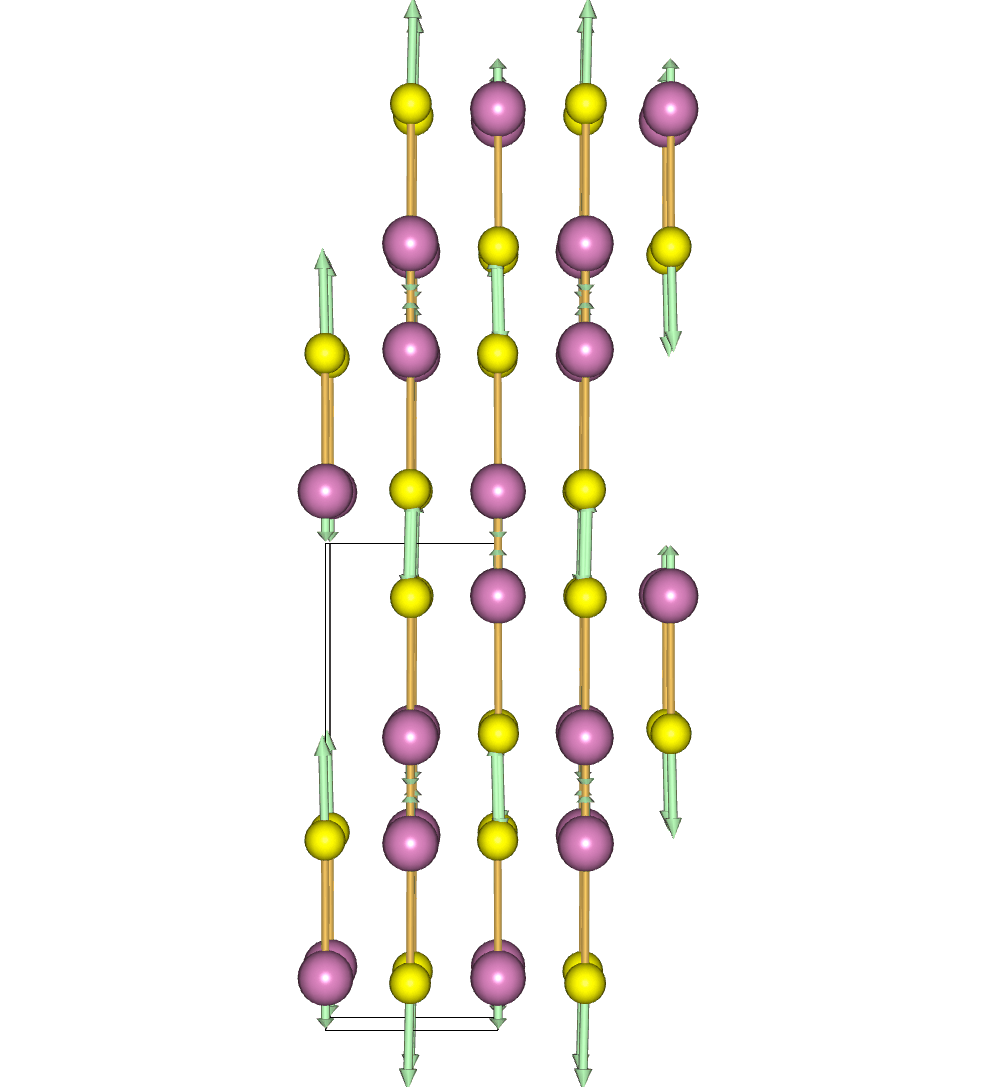


**Fig. S10.** Vibrational modes of 1.64, 3.13, 7.34, and 9.03 THz in InS at Γ point. In and S atoms are marked in purple and yellow, respectively.
